# Nanoscale Dodecahedral and Fullerene-Type Organoboroxine and Borazine Cages from Planar Building Units

**DOI:** 10.1021/acs.nanolett.4c01024

**Published:** 2024-05-07

**Authors:** Mario Sánchez, Jonas Baltrusaitis, María G. Vasquez-Ríos, Gonzalo Campillo-Alvarado, Leonard R. MacGillivray, Herbert Höpfl

**Affiliations:** † Centro de Investigación en Materiales Avanzados, S.C., Alianza Norte 202, Parque de Investigación en Innovación Tecnológica (PIIT), Carretera Monterrey-Aeropuerto Km 11, Apodaca 66628, Nuevo León, México; ‡ Department of Chemical and Biomolecular Engineering, Lehigh University, Research Drive 111, Bethlehem, Pennsylvania 18015, United States; § Département de Chimie, Université de Sherbrooke, Sherbrooke J1K 2R1, Québec, Canada; ∥ Department of Chemistry, Reed College, Portland, Oregon 97202, United States; ⊥ Centro de Investigaciones Químicas, Instituto de Investigación en Ciencias Básicas y Aplicadas, Universidad Autónoma del Estado de Morelos, Av. Universidad 1001, Chamilpa, Cuernavaca 62209, Morelos, México

**Keywords:** boron cages, boroxines, self-assembly, polyhedra, fullerene-type structures

## Abstract

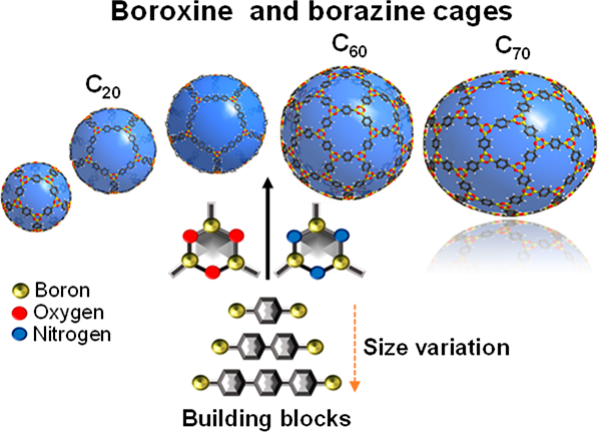

Boroxine- and borazine-cage analogs to C_20_, C_60_, and C_70_ were calculated and compared in terms of structure, strain indicators, and physical properties relevant to nanoscale applications. The results show C_60_ and C_70_ type cages are less strained than the smaller congener, primarily due to minimized bending in the B-arylene-B segments. The smallest cage calculated has a diameter of 2.4 nm, which increases up to 4.9 nm by either variation of the polyhedron (C_20_ < C_60_ < C_70_-type cage) or organic spacer elongation between boron centers. All calculated cages are porous (apertures ranging from 0.6 to 1.9 nm). Molecular electrostatic potential and Hirshfeld population analysis revealed both nucleophilic and electrophilic sites in the interior and exterior cage surfaces. HOMO–LUMO gaps range from 3.98 to 4.89 eV and 5.10–5.18 eV for the boroxine- and borazine-cages, respectively. Our findings provide insights into the design and properties of highly porous boroxine and borazine cages for nanoscience.

The rapidly growing field of organic and metal–organic cage compounds and nanomaterials has garnered significant attention owing to numerous applications.^1−4^ These systems are commonly formed through self-assembly driven by reversible noncovalent^5^ and/or covalent^6^ bond formation. Major inspirations for construction originate from similar processes in Nature that lead to viral capsids and fullerenes. While significant progress has been made in cage design, there remains an ongoing need to identify new abiotic building blocks to generate cage compounds and materials.

In this context, the generation of organic cages through dynamic covalent bond formation can be achieved from building blocks, or tectons,^7,8^ with chemical functions in precise geometric arrangements.^9,10^ Most organic cages to date are derived from reactions of components that *de facto* provide curvature (e.g., bowl-shaped calixarenes).^9,11−13^ From a topological standpoint, a tecton used to generate a cage needs to meet two basic design criteria: (i) multitopic bonding that adheres to symmetry demands to link the components in 3D space (i.e., tetrahedral, cubic, icosahedral) and (ii) sufficient curvature for space to be enclosed. Accordingly, the identification of tectons that provide such chemical information remains a challenge.

Here, we report on tectons based on organoboroxines that while largely planar support the generation of large self-assembled B-based cages. We use computational chemistry and evidence from the literature to support B_3_O_3_-rings bridged by flat linear spacers to generate the spherical cages **COBOC-*n*-BDBA** (where: **COBOC** = **C**ovalent **O**rganic **B**or**O**xine **C**age; ***n*** = 20, 60, or 70 B_3_O_3_-rings; **BDBA** = 1,4-benzenediboronic acid). The structures of the cages are realized using geometry-optimization by DFT-calculations at the B-P86/def-SV(P) level.^14,15^ The sizes of the cages range from 2 to 5 nm and, we show, can provide a roadmap to isomorphous assemblies with organoborazine linkers.

A prominent class of compounds derived from organoboronic acids is organoboroxines, which are based on trinuclear cyclic structures (Figure 1a).^16−20^ The reversibility of boroxine formation and accessibility of the boron p-orbital by Lewis bases^8,16−18,21−23^ has led to significant interest in multicomponent covalent-organic frameworks (COFs)^19,24^ where planar di- and/or multitopic boronic acids link the B_3_O_3_-ring systems in two (2D) and three (3D) dimensions (Scheme S1, Supporting Information).

**Figure 1:**
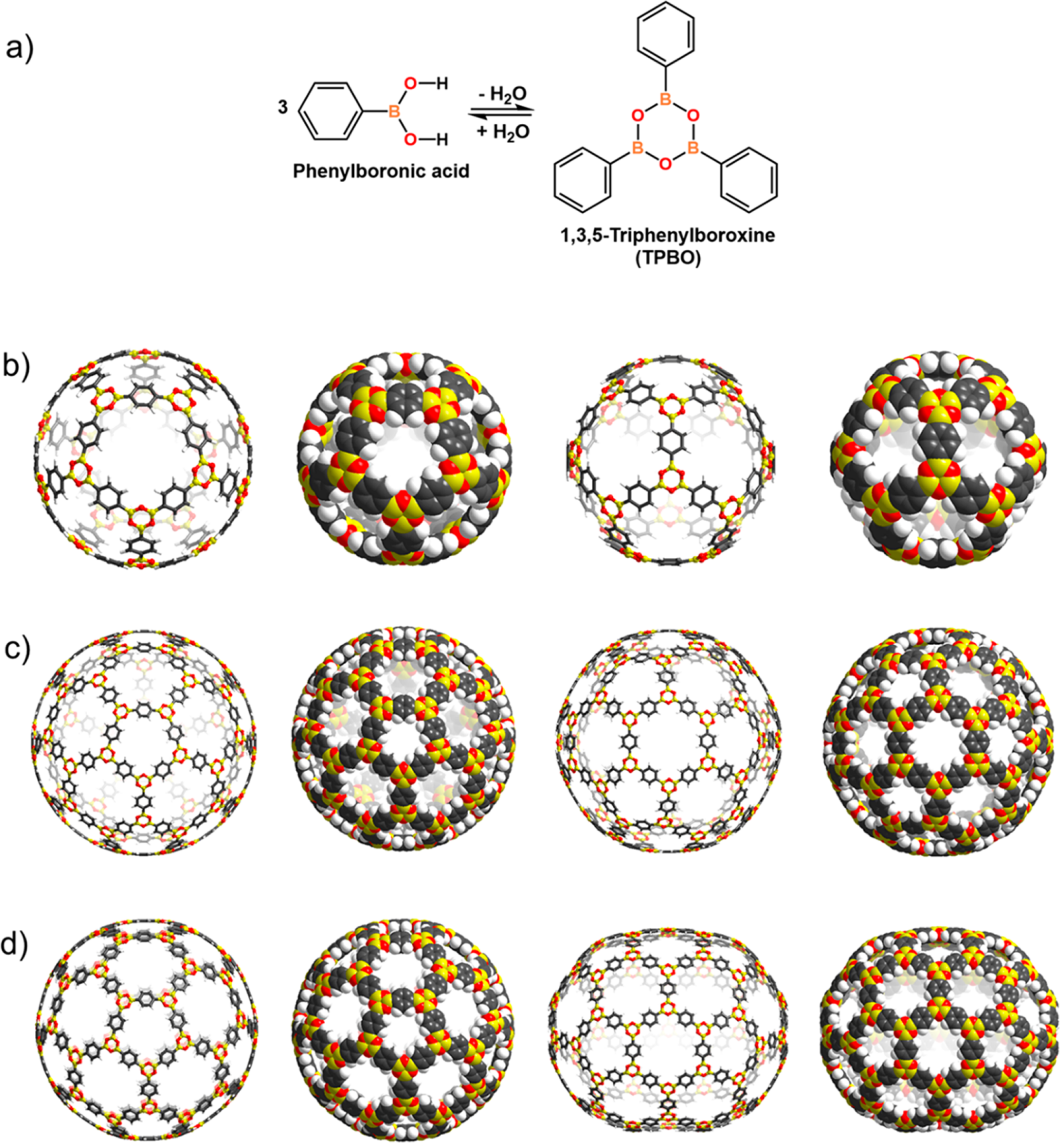
(a) Chemical drawing of a representative boronic acid, i.e., phenylboronic acid, and 1,3,5-triphenylboroxine resulting from the condensation reaction, and perspectives in stick- and space-filling of the calculated boroxine structures: (b) **COBOC-20-BDBA**, (c) **COBOC-60-BDBA**, and (d) **COBOC-70-BDBA**.

While there has been an upsurge to develop COFs based on boronic esters and boroxines,^25−27^ there is markedly less attention to develop discrete 3D cages and, more specifically, spherical cages (i.e., cubic symmetry). B-based cages^11,28^ have been reported wherein bent (i.e., nonlinear) diboronic acid linkers provide the necessary curvature to generate convex surfaces along the cage structures. The boroxine structure itself exhibits susceptibility to curvature owing to weak π-bonding of the B–O bonds. Boronic acid based 2D COFs on metal surfaces have also been shown to exhibit defects consisting in five- and seven-membered B_3_O_3_-rings that provide curvature (Scheme S2, Supporting Information).^29^ Related nanoaggregates and nanotubes have been reported to form via condensations involving boron monoxide linkages,^30,31^ while spherical hydrogen-bonded assemblies of boric and metaboric acid have also been explored.^32−36^ In a minimalist case, a cage with organoboroxines would utilize the flexible *D*_3*h*_ symmetric B_3_O_3_-ring system to supply curvature that propagates the organic linkers at angles approximating 120° akin to the role of C atoms of C_60_ (Scheme S3, Supporting Information). Such a design, however, has not been addressed and can be considered counterintuitive given the planar nature of the B_3_O_3_-ring building blocks. In general, the formation of such cages is predicated on design strategies that adhere to tetrahedral, cubic, and icosahedral symmetries, with Platonic and Archimedean solids serving as a model for spheroid designs.^37,38^ Given that Platonic and Archimedean solids are built from planar building blocks in the form of polygons, we hypothesized that the flexibility of the B_3_O_3_-ring system while largely planar could support the formation of 3D cages. The aromatic ring systems of the organic linkers that we study here while linear (i.e., not bent) would also be sufficiently flexible to provide curvature.

Structures of three initial cages **COBOC-*n*-BDBA** (where: ***n*** = 20, 60, 70) were determined using DFT calculations (Figure 1b-d). For the cages, the B–O bond lengths in the range of 1.389 to 1.391 Å are the same as in solid 1,3,5-triphenylboroxine (TPBO), for which a range of (1.384(9)–1.386(9) Å) was determined by SCXRD analysis.^39^ The p_π_–p_π_ bonding is, thus, preserved despite the curvature. The O–B–O and B–O–B bond angles are in the range of 118.50°–118.99° and 119.30°–122.05°, respectively, being also in good agreement with TPBO [117.3(6)°–118.8(5)° and 121.3(5)°–122.0(5)°]. The B–C bond lengths (1.563–1.564 Å) of the cages are close to the upper limit of the bond distance range [1.535(8)–1.544(8) Å] established by experiment (d ± 3σ).

The B-cages exhibit diameters of nanoscale dimensions. The diameters as defined by opposite B_3_O_3_-rings for the C_20_ and C_60_ analogues **COBOC-20-BDBA** (2.40 nm) and **COBOC-60-BDBA** (4.32 nm) correspond to approximate 3.5- and 6.0-fold increases compared to C_60_ (0.70 nm).^40^ The sizes compare favorably to a tetrahedral tetraboroxine cage (3.2 nm).^11^**COBOC-70-BDBA** is approximately spheroidal being defined by transversal (4.18 nm) and longitudinal (4.92 nm) diameters. The windows comprised by the 5- and 6-membered boroxine macrocyclic rings along the surfaces exhibit appreciable sized apertures to the cage interiors (0.66 and 0.89 nm, respectively). The sizes of the macrocycles are in good agreement with reported sites in defects in layers of a 2D boroxine COF derived from **BDBA**.^29^

The B_3_O_3_-ring system in combination with the organic linkers provides a convex surface for each cage. The curvatures for **COBOC-20-BDBA**, **COBOC-60-BDBA**, and **COBOC-70-BDBA** are realized by bending of the B–C_6_H_4_–B segments in relation to the B_3_O_3_-rings, which are practically planar. Bending is defined by the angle between straight lines involving calculated centroids of adjacent boroxine units and C_6_H_4_-fragments. We define a system labeled α_55_ for fragments located in adjacent pentagonal polygons, α_56_ for junctions of pentagonal and hexagonal polygons, and α_66_ for junctions of two hexagonal polygons (Figure 2).

**Figure 2:**
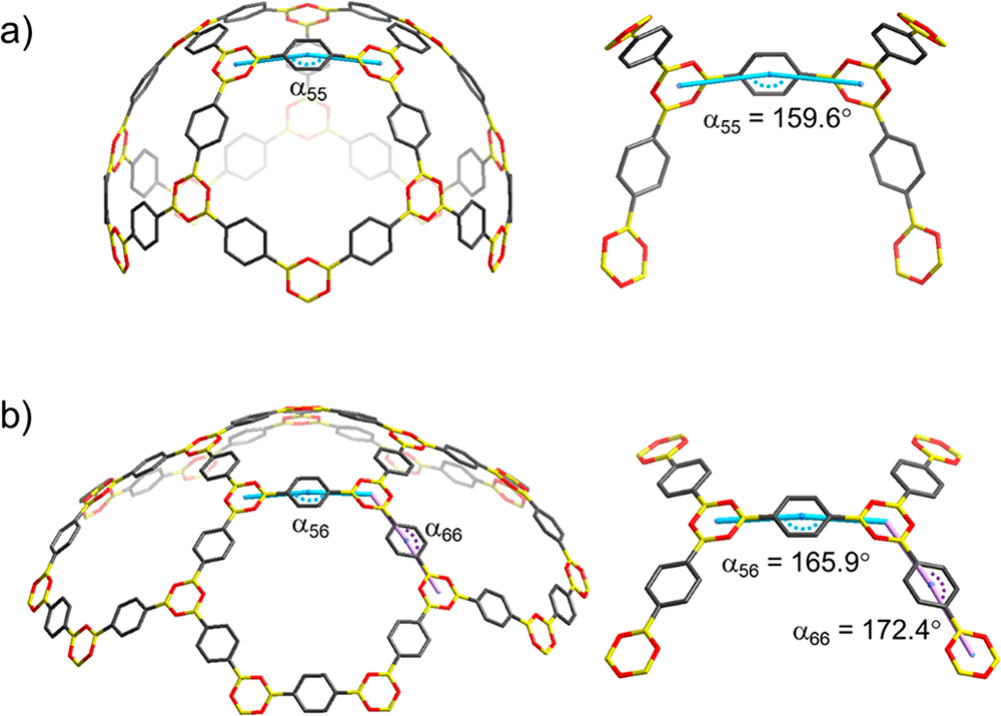
Bending in B_3_O_3_–C_6_H_4_–B_3_O_3_: (a) **COBOC-20-BDBA** and (b) **COBOC-60-BDBA**. Note: α_55_, α_56_, and α_66_ correspond to angles using calculated centroids in adjacent boroxine units and the C_6_H_4_-fragments.

Bending in smaller **COBOC-20-BDBA** (α_55_ = 159.6°) is, thus, significantly larger than in **COBOC-60-BDBA** (α_56_ = 165.9°; α_66_ = 172.4°) and **COBOC-70-BDBA** (α_56_ = 165.74°–166.35°; α_66_ = 171.79°–173.93°). The corresponding bending angles for TPBO molecules in its solid-state structure range from 173.3° to 179.0°.^39^ Overall, bending can be considered to diminish enthalpy, although the cage formation is entropically favored compared to a 2D boroxine layer structure. We calculate the relative stabilities of the cages as C_70_ ≈ C_60_ > C_20_, with relative strain energies per minimum formula unit (B_3_O_3_C_9_H_6_) versus **COBOC-70-BDBA** being 0.17 kcal mol^–1^ (**COBOC-60-BDBA**) and 2.54 kcal mol^–1^ (**COBOC-20-BDBA**). For comparison, the enthalpy of formation for the transformation of C_60_ per formula unit C atom to C_graphite_ is 9.26 kcal mol^–1^.^40^

Organic cages generally exhibit high symmetries, with spheroids conforming to structures of the Platonic and Archimedean solids.^37,38,41^ The structures of **COBOC-20-BDBA** and **COBOC-60-BDBA** conform to a dodecahedron (Platonic) and truncated icosahedron (Archimedean), respectively. The structure of **COBOC-70-BDBA** is approximately spheroidal, being elongated similar to C_70_ and exhibiting a shape of a rugby ball.^40^

The Platonic and Archimedean solids are also synthetic roadmaps to cages. Given the structural similarities of boroxine and borazines R_3_B_3_N_3_R_3_′, isomorphous cages of **COBNC-20-BDBA** and **COBNC-60-BDBA** (COBNC = **C**ovalent **O**rganic **B**orazi**N**e **C**age) were calculated (Figure 3).^42^ For reference, the phenyl rings in 2,4,6-triphenylborazine (Scheme S4, Supporting Information) twist (27.7°) give a propeller-like structure, which originates from H···H repulsions of C–H and N–H hydrogen atoms. The B–N distances for **COBNC-20-BDBA** and **COBNC-60-BDBA** are comparably elongated (1.442–1.446 Å) in reference to the experimental data reported for 2,4,6-triphenylborazine (1.424(4) Å), which indicates reduced p_π_–p_π_ bonding.^43^ The phenylene ring twists result in borazine cages differing from the boroxine analogues (Figure 3). Both the N–B–N and B–N–B angles are moderately decreased (2°) while the mutual twists of the phenylene and borazine rings are larger versus 2,4,6-triphenylborazine (**COBNC-20-BDBA** 26°–30°; **COBNC-60-BDBA** 29°–32°). The diameters of **COBNC-20-BDBA** and **COBNC-60-BDBA** are also of nanoscale dimensions, with bending indicating higher strain in the smaller cage (α_55_ ≈ 158°–164° in **COBNC-20-BDBA**; α_56_ ≈ 166°–167° and α_66_ ≈ 170°–171° in **COBNC-60-BDBA**, Figure S1, Supporting Information). The relative strain energy for **COBNC-20-BDBA** per minimum formula unit (B_3_N_3_C_9_H_9_) is 3.58 kcal mol^–1^, which is larger compared to the boroxine assemblies and can be attributed to elevated π-bonding character of B–N bonds.

**Figure 3:**
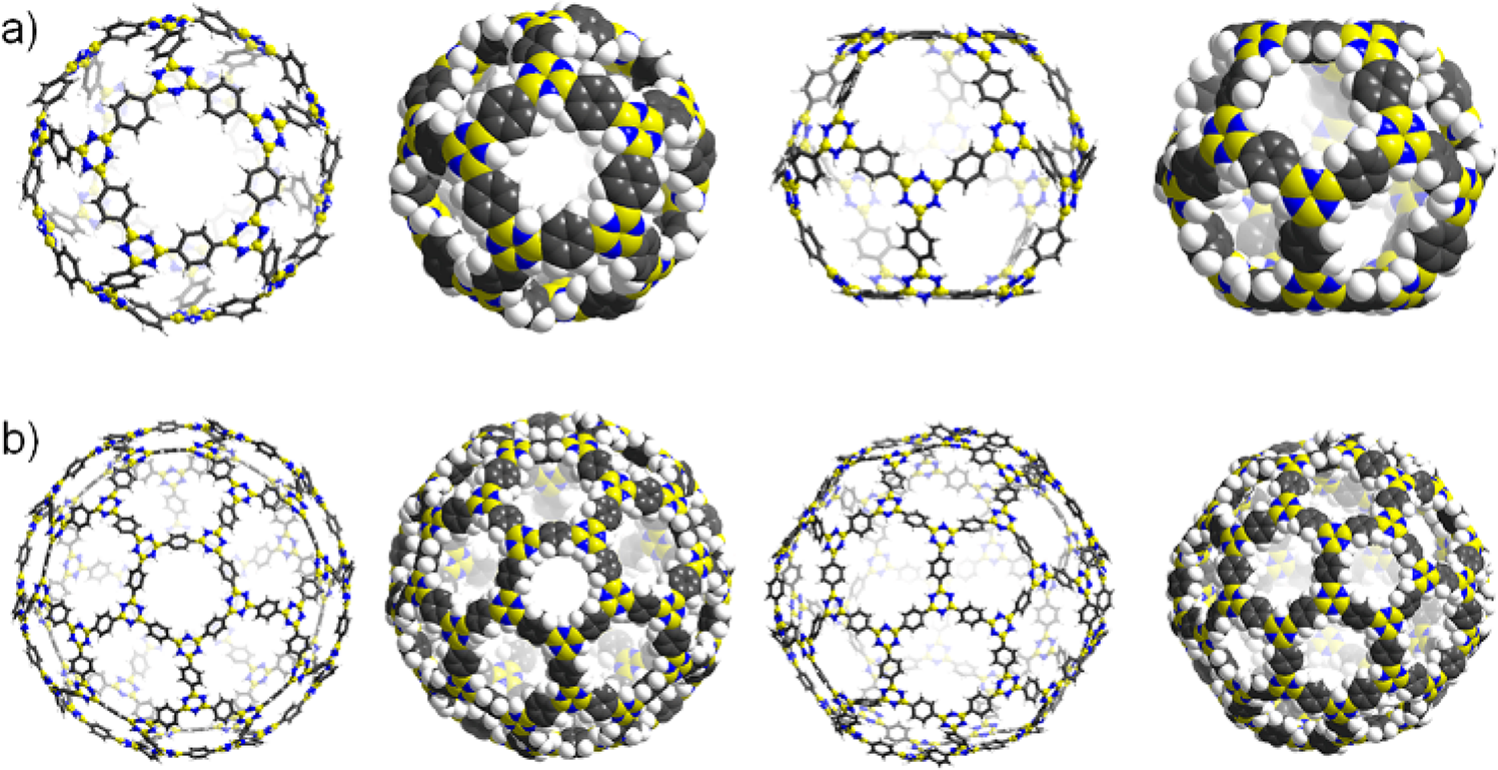
Perspectives of calculated borazine cages: (a) **COBNC-20-BDBA** and (b) **COBNC-60-BDBA**.

Elongated bi- and terphenyl congeners were calculated as the very large cages **COBOC-20-BPDBA** and **COBOC-20-TPDBA** (BPDBA = 4,4′-biphenyldiboronic acid; TPDBA = 4,4″-*para*-terphenyldiboronic acid) of diameters 3.59 and 4.77 nm, respectively (Figure 4). The bending angles α_55_ are diminished by approximately 1° and 2° for **COBOC-20-BPDBA** (α_55_ = 160.6°) and **COBOC-20-TPDBA** (α_55_ = 160.62°–163.39°), respectively, compared to **COBOC-20-BDBA** (α_55_ = 159.6°) (Figure S2, Supporting Information).

**Figure 4:**
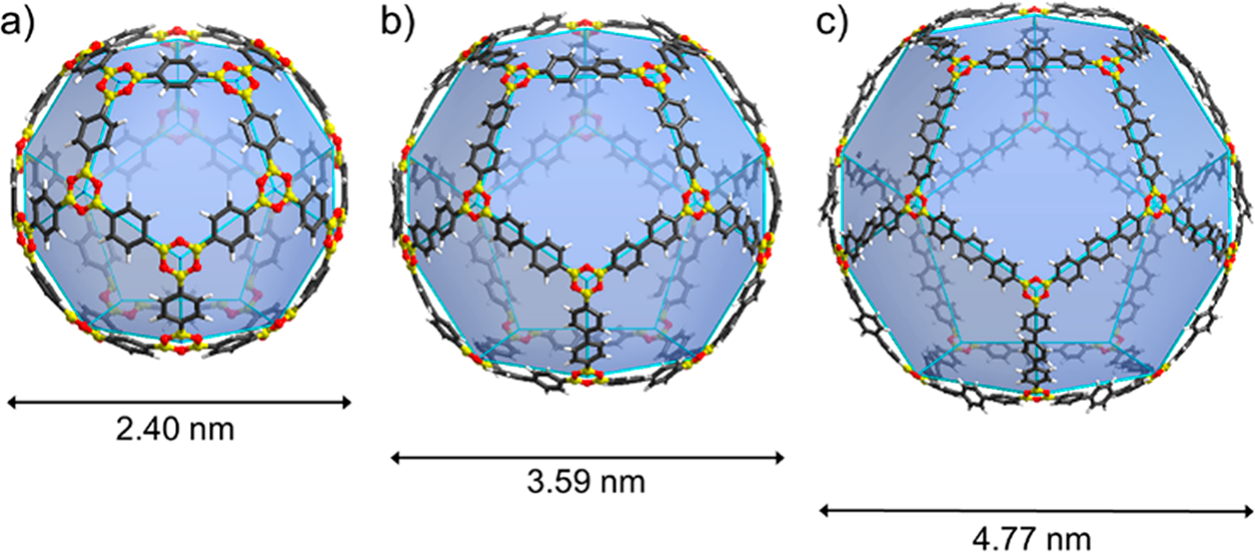
Perspectives of calculated boroxine structures: (a) **COBOC-20-BDBA**, (b) **COBOC-20-BPDBA**, and (c) **COBOC-20-TPDBA**.

The inner and outer surfaces of the B-cages were studied using molecular electrostatic potential (MESP) maps (Figure 5). Negative potentials (orange and yellow) are concentrated at the π-clouds, as well as the O and N atoms. The regions are expected to interact with electrophiles, including those for hydrogen bonding. Positive potentials (blue) are concentrated at the H and B atoms, which are expected to act as targets for nucleophilic attack or donors for hydrogen bonding. The windows for access to cage interiors exhibit positive potentials, with the O atom lone pairs shielded by the aromatic C–H hydrogens. The MESP map is confirmed by the distribution of the Hirshfeld atomic charges (Table S1, Supporting Information). We note that the MESP map contrasts C_60_, which is entirely positive in the cage.^44^ For the B-cages, positive and negative regions are concentrated at the inner and outer surfaces. The B-cages can, thus, be expected to serve as hosts of positively and negatively charged guests, with negative species favored in bypassing positive surfaces of the entrance windows.

**Figure 5:**
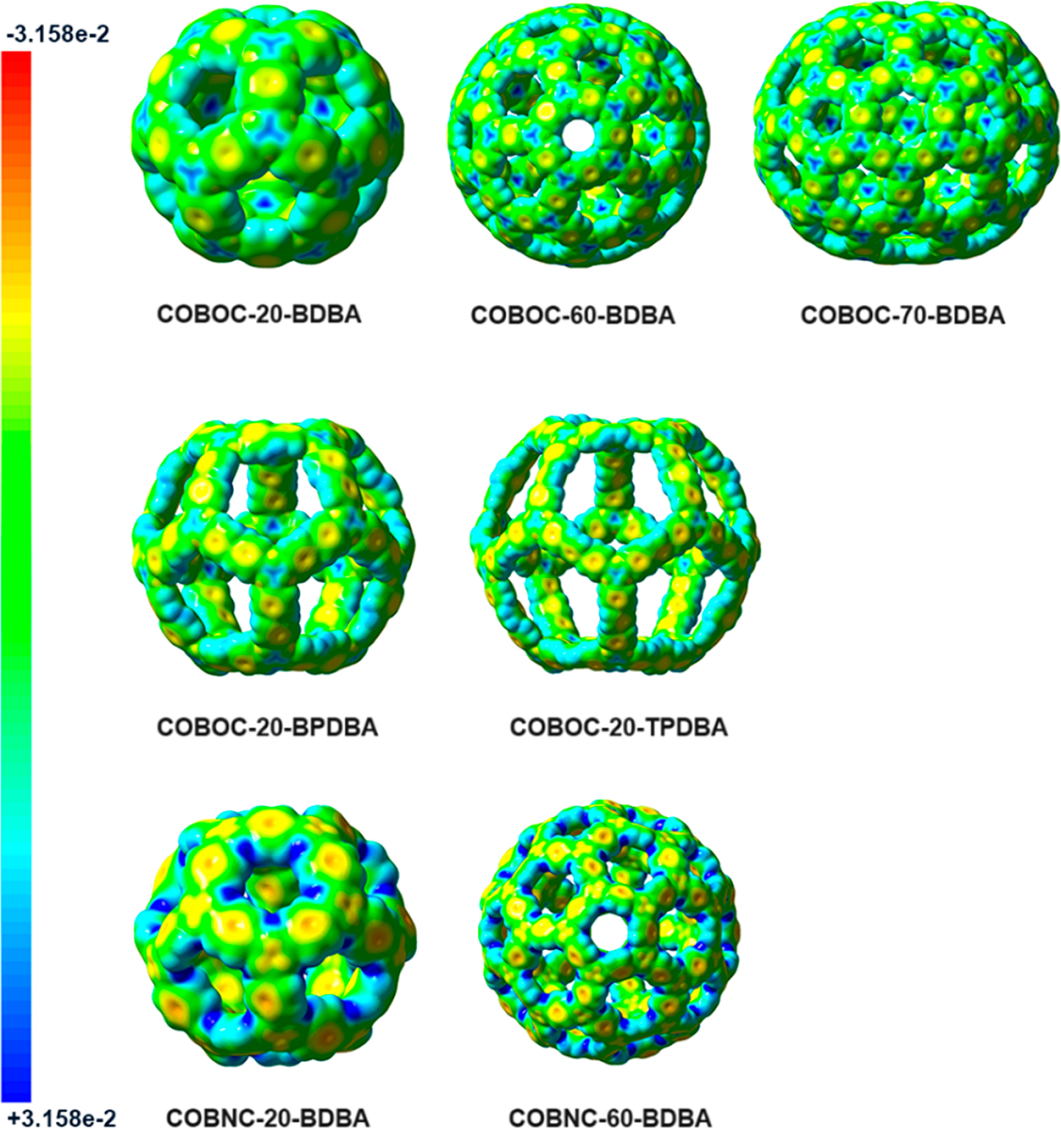
Molecular electrostatic potential maps (isoval = 0.0004) for the boroxine and borazine cages analyzed herein.

The DFT calculations show the HOMO–LUMO gaps (RI-B3LYP/def2-TZVP theory) (Table S1, see Supporting Information) for the B-cages to be size-dependent, being largest (4.89 eV) for **COBOC-20-BDBA** and smallest for the elongated congener **COBOC-20-TPDBA** (3.98 eV). The HOMO-MOs are localized mainly on the phenylene C atoms, while the LUMO-MOs include the B atoms. The gaps for the borazine cages, **COBNC-20-BDBA** (5.10 eV) and **COBNC-60-BDBA** (5.18 eV), are greater than those for the boroxines.

In summary, diboronic acids are single-tecton candidates to generate spherical cage assemblies through boroxine and borazine formation. Such cages offer high surface area, a tunable cavity, accessible window sizes, and low weight. We regard the cages accessible from a synthetic standpoint. 2D and 3D COFs are of high thermal stabilities (i.e., 500 °C), which are generally expected for the spherical cages.^29,45^ Boroxine-based materials are robust in dry organic solvents and can be stabilized using substituents (e.g., bulky groups, electron donation).^16,46^ B–O bonds can also “heal” in assembly reactions (i.e., repairing defects).^18,29^ We also note that borazines are relatively less stable against hydrolysis. We believe that our findings lay groundwork to prepare the large cages. The ubiquity of the boronic acids in Suzuki couplings means that a large diversity of boronic acids is commercially available, with synthetic routes to diboronic acids being well-established (**BDBA**, **BPDBA**, and **TPDBA**).^47−49^ Diverse synthetic strategies (e.g., solvothermal, microwave-assisted) are also available to transform boronic acids to boroxines. There will, moreover, be a challenge to establish strategies to preorganize the boronic acid precursors (e.g., soft templates).^50−52^ It is likely that the cages can be tailored by facile substitution of the boronic acid linkers, allowing for the diversification of the architectures, work on host–guest chemistry, and surface studies. The insights can pave the way to advance nanoconfinement and explore applications of the versatile structures. Boroxine-based materials have also a promising profile environmentally, with boroxines being easily reconverted and used in the context of the many applications of boronic acids.^20,53−55^

## Supporting Information Available

Experimental section, additional schemes (Schemes S1–S4), additional figures (Figures S1 and S2), tables (Table S1), and Cartesian coordinates for the atoms in the calculated structures. (PDF)
