# Supplementary material for: Nanoscale Dodecahedral and Fullerene-Type Organoboroxine and Borazine Cages from Planar Building Units
Source: Nano Lett. 2024 May 7;24(19):5824–30. doi: 10.1021/acs.nanolett.4c01024 (PMC11100284; doi:10.1021/acs.nanolett.4c01024)
Supplement: Supplementary file 1 — nl4c01024_si_001.pdf [file nl4c01024_si_001.pdf]

Nanoscale Dodecahedral and Fullerene-type Organoboroxine and Borazine Cages from Planar Building Units

Mario Sánchez,[a] Jonas Baltrusaitis,*[b] María G. Vasquez-Ríos,[c] Gonzalo Campillo-Alvarado,[d] Leonard R. MacGillivray,*[c] Herbert Höpfl,*[e]*

[a] Centro de Investigación en Materiales Avanzados, S.C., Alianza Norte 202, Parque de Investigación en Innovación Tecnológica (PIIT), Carretera Monterrey-Aeropuerto Km 11, Apodaca 66628, Nuevo León, México.

[b] Department of Chemical and Biomolecular Engineering, Lehigh University, Research Drive 111, Bethlehem, PA 18015, USA.

[c] Département de Chimie, Université de Sherbrooke, Sherbrooke, Québec, Canada.

[d] Department of Chemistry, Reed College, Portland, OR 97202, USA.

[e] Centro de Investigaciones Químicas, Instituto de Investigación en Ciencias Básicas y Aplicadas, Universidad Autónoma del Estado de Morelos, Av. Universidad 1001, Chamilpa, Cuernavaca 62209, Morelos, México.

*E-mail: mario.sanchez@cimav.edu.mx, job314@lehigh.edu, Leonard.MacGillivray2@usherbrooke.ca and hhopfl@uaem.mx

Supporting Information

Table of Contents

1. Experimental section
2. Additional schemes
3. Additional figures
4. Tables
5. Cartesian coordinates for the atoms in the calculated structures

1. Experimental section

Quantum chemical calculations to obtain the geometry-optimized minimum energy structures for the compounds studied herein were performed using the B-P86/def-SV(P)[1,2] method implemented in the Turbomole V7.6 2022 package[3] and m4 grid.[4] Multipole accelerated RI-J5[5] and symmetry restraints were used to speed up calculation and auxiliary basis sets[4,6] were employed to approximate Coulomb potentials. Optimization convergence was signaled when the energy change was less than 0.1E-6 and the geometry gradient was less than 0.1E-3 atomic units. Harmonic vibrational modes were calculated at the same level of theory using numerical differentiation to characterize the structures as true minima on the potential energy surface. Single-point energies were calculated at the RI-B3LYP/def2-TZVP[7,8] level of theory. Molecular electrostatic potential (MESP)[9,10] maps and Hirshfeld charges[11] were calculated with the B-P86/def2-SVP[1,12] method implemented in the Gaussian 16 program.[13] In the MESP maps, negative potential is represented by orange/yellow color and positive potential is given in blue color.

References

(1) Becke, A. D. Density-Functional Exchange-Energy Approximation with Correct Asymptotic Behavior. *Phys. Rev. A* **1988**, *38* (6), 3098–3100.
(2) Schäfer, A.; Horn, H.; Ahlrichs, R. Fully Optimized Contracted Gaussian Basis Sets for Atoms Li to Kr. *J. Chem. Phys.* **1992**, *97* (4), 2571–2577.
(3) TURBOMOLE V7.6 2022, a Development of University of Karlsruhe and Forschungszentrum Karlsruhe GmbH, **1989-2007**, TURBOMOLE GmbH; Available from https://www.turbomole.org
(4) Eichkorn, K.; Weigend, F.; Treutler, O.; Ahlrichs, R. Auxiliary Basis Sets for Main Row Atoms and Transition Metals and Their Use to Approximate Coulomb Potentials. *Theor. Chem. Acc. Theory Comput. Model. Theor. Chim. Acta* **1997**, *97* (1–4), 119–124.
(5) Sierka, M.; Hogekamp, A.; Ahlrichs, R. Fast Evaluation of the Coulomb Potential for Electron Densities Using Multipole Accelerated Resolution of Identity Approximation. *J. Chem. Phys.* **2003**, *118* (20), 9136–9148.
(6) Eichkorn, K.; Treutler, O.; Öhm, H.; Häser, M.; Ahlrichs, R. Auxiliary Basis Sets to Approximate Coulomb Potentials. *Chem. Phys. Lett.* **1995**, *242* (6), 652–660.
(7) Lee, C.; Yang, W.; Parr, R. G. Development of the Colle-Salvetti Correlation-Energy Formula into a Functional of the Electron Density. *Phys. Rev. B* **1988**, *37* (2), 785–789.
(8) Weigend, F.; Ahlrichs, R. Balanced Basis Sets of Split Valence, Triple Zeta Valence and Quadruple Zeta Valence Quality for H to Rn: Design and Assessment of Accuracy. *Phys. Chem. Chem. Phys.* **2005**, *7* (18), 3297.
(9) Murray, J. S.; Sen, K. (Eds.). Molecular Electrostatic Potentials: Concepts and Applications; Elsevier: Amsterdam, 1996.

(10) Gadre S. R.; Shirsat, R. N. Electrostatics of Atoms and Molecules. Universities Press: Hyderabad, India, 2000.
(11) Ritchie, J. P.; Bachrach, S. M. Some Methods and Applications of Electron Density Distribution Analysis. *J. Comput. Chem.* **1987**, *8* (4), 499–509.
(12) Weigend, F. Accurate Coulomb-Fitting Basis Sets for H to Rn. *Phys. Chem. Chem. Phys.* **2006**, *8* (9), 1057.
(13) Gaussian 16, Revision **C.01**, Frisch, M. J.; Trucks, G. W.; Schlegel, H. B.; Scuseria, G. E.; Robb, M. A.; Cheeseman, J. R.; Scalmani, G.; Barone, V.; Petersson, G. A.; Nakatsuji, H.; Li, X.; Caricato, M.; Marenich, A. V.; Bloino, J.; Janesko, B. G.; Gomperts, R.; Mennucci, B.; Hratchian, H. P.; Ortiz, J. V.; Izmaylov, A. F.; Sonnenberg, J. L.; Williams-Young, D.; Ding, F.; Lipparini, F.; Egidi, F.; Goings, J.; Peng, B.; Petrone, A.; Henderson, T.; Ranasinghe, D.; Zakrzewski, V. G.; Gao, J.; Rega, N.; Zheng, G.; Liang, W.; Hada, M.; Ehara, M.; Toyota, K.; Fukuda, R.; Hasegawa, J.; Ishida, M.; Nakajima, T.; Honda, Y.; Kitao, O.; Nakai, H.; Vreven, T.; Throssell, K.; Montgomery, J. A., Jr.; Peralta, J. E.; Ogliaro, F.; Bearpark, M. J.; Heyd, J. J.; Brothers, E. N.; Kudin, K. N.; Staroverov, V. N.; Keith, T. A.; Kobayashi, R.; Normand, J.; Raghavachari, K.; Rendell, A. P.; Burant, J. C.; Iyengar, S. S.; Tomasi, J.; Cossi, M.; Millam, J. M.; Klene, M.; Adamo, C.; Cammi, R.; Ochterski, J. W.; Martin, R. L.; Morokuma, K.; Farkas, O.; Foresman, J. B.; Fox, D. J. Gaussian, Inc., Wallingford CT, **2**016.

2. Additional schemes

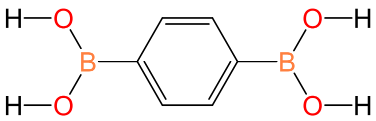

1,4-Benzenediboronic acid (BDBA)

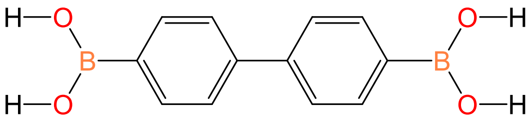

4,4'-Biphenyldiboronic acid (BPDBA)

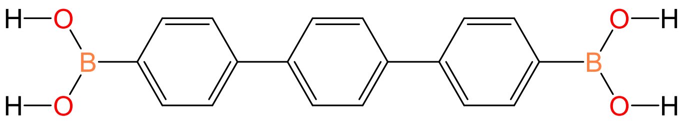

4,4''-para-Terphenyldiboronic acid (TPDBA)

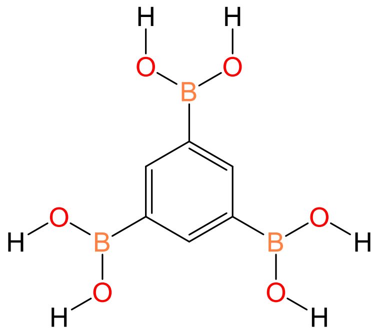

1,3,5-Benzenetriboronic acid (BTBA)

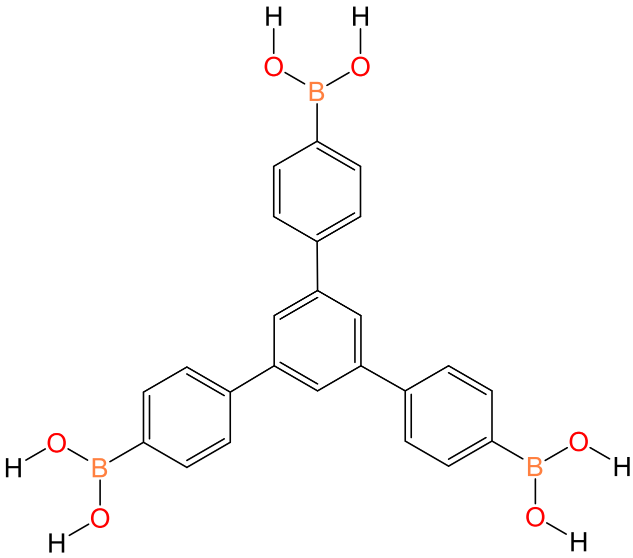

1,3,5-Tris(4-phenylboronic acid) (TPBA)

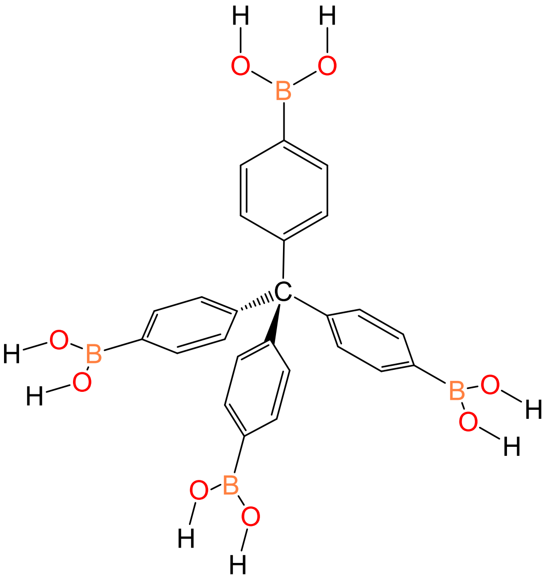

Methane-tetrakis(4-phenylboronic acid) (MTPBA)

Scheme S1. Chemical drawings of representative di-, tri- or tetranuclear boronic acids.

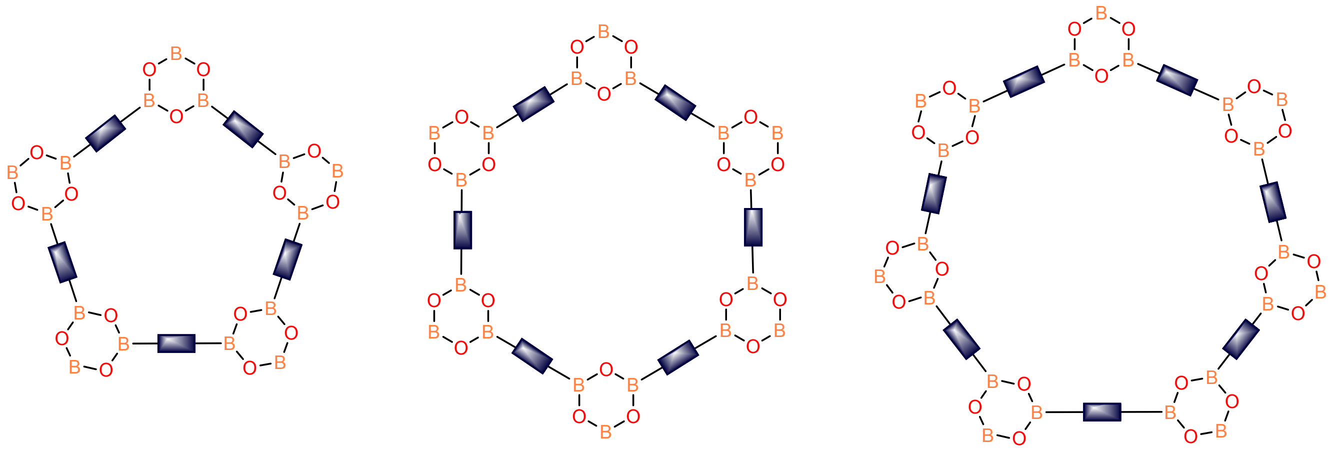

Scheme S2. 2D layers based on boroxines derived from BDBA can include defects in form of macrocycles having five (left) and seven (right) B_3O_3-rings, in addition to the commonly observed assembly (middle).

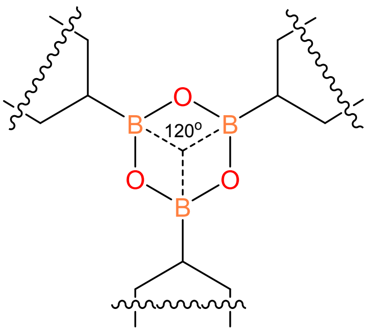

Scheme S3. Schematic illustration of the geometric particularities of boroxines, enabling the generation of 2D nets analogous to graphite.

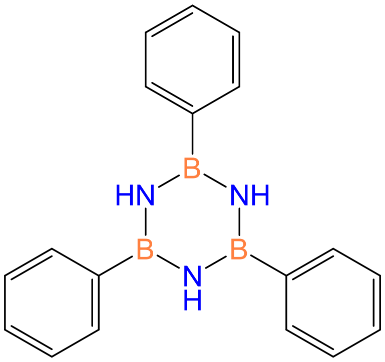

2,4,6-Triphenylborazine

Scheme S4. Chemical drawing of 2,4,6-triphenylborazine.

3. Additional figures

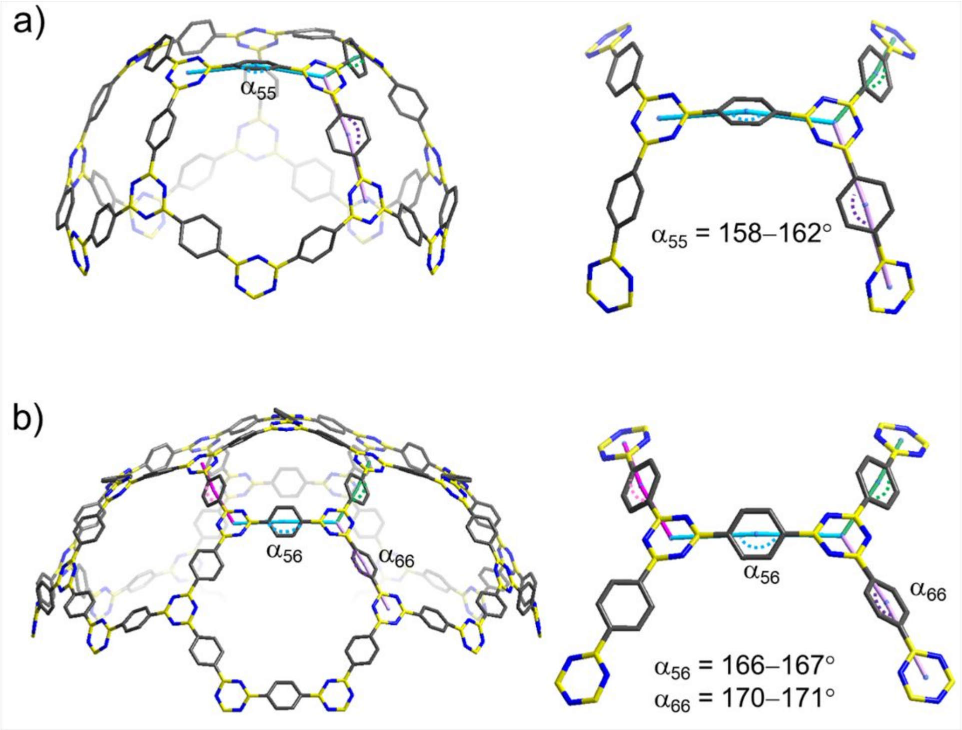

Figure S1. Geometric features of the bending in the B_3N_3–C_6H_4–B_3N_3 segments observed in cages (a) **COBNC-20-BDBA** and (b) **COBNC-60-BDBA**. Note: The bending values α_{55}, α_{56} and α_{66} correspond to the angles formed between straight lines originated from connection of the calculated centroids in adjacent borazine units and the C_6H_4-fragments.

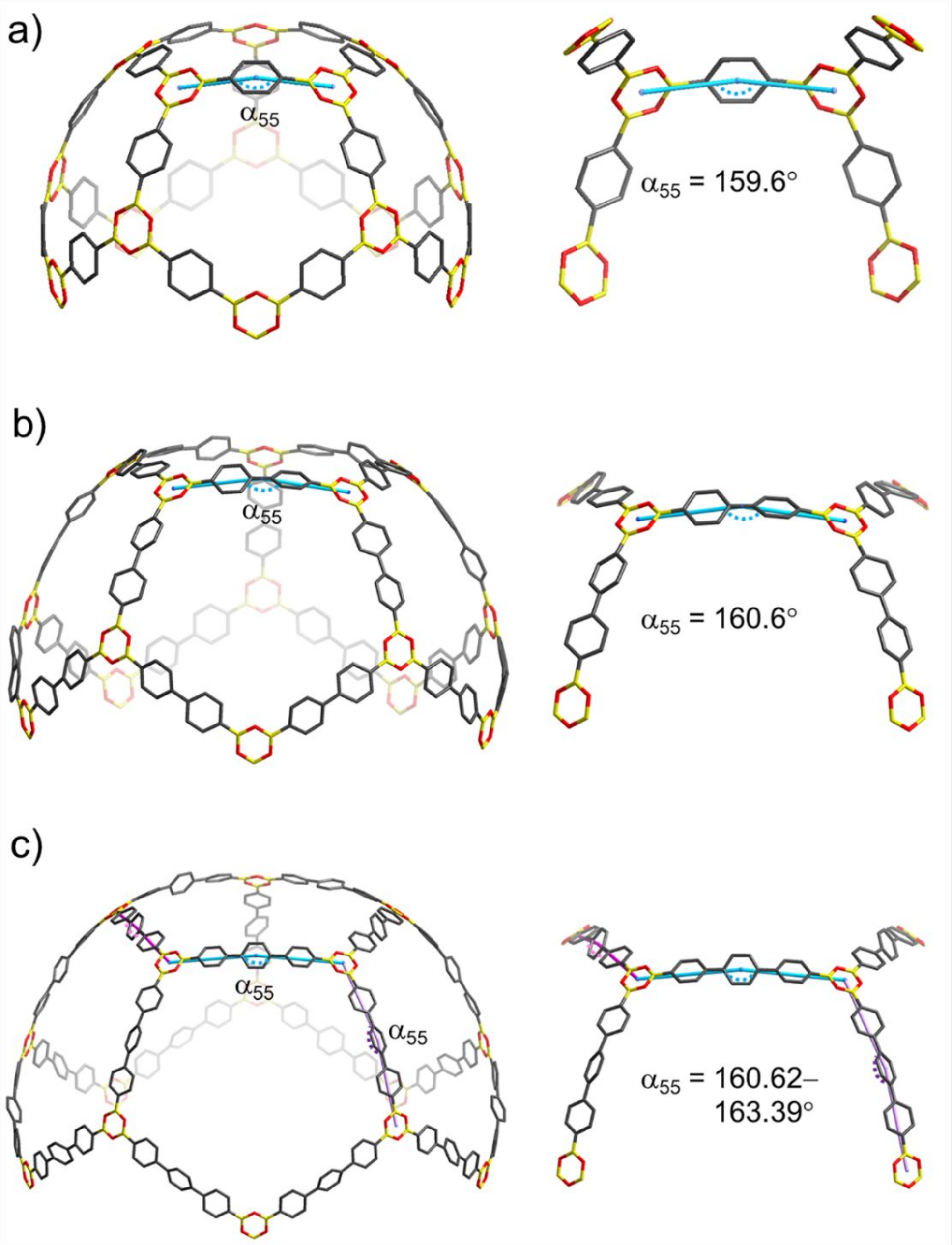

Figure S2. Geometric features of the bending in the B_3O_3–C_6H_4–B_3O_3 segments observed in cages **COBOC-20-BDBA** (a), **COBOC-20-BPDBA** (b) and **COBOC-20-TPDBA** (c). Note: The bending value α_{55} corresponds to the angle formed between straight lines straight lines originated from connection of the calculated centroids in adjacent boroxine units and the organic connector.

4. Tables

Table S1. HOMO-LUMO band gap value (eV)[a] and selected Hirshfeld charges (Q, e)[b] for the boroxine and borazine-cages studied herein.

	Gap	Q(B)	Q(O)	Q(N)	Q(H_N)	Q(C_B)
**COBOC-20-BDBA**	4.89	+0.234	-0.193	-	-	-0.070
**COBOC-60-BDBA**	4.89	+0.233<br>+0.234	[-0.192] – [-0.194]	-	-	-0.070
**COBOC-70-BDBA**	4.89	+0.233<br>+0.234	[-0.191] – [-0.194]	-	-	-0.070
**COBOC-20-BPDBA**	4.19	[c]	[c]	-	-	[c]
**COBOC-20-TPDBA**	3.98	[c]	[c]	-	-	[c]
**COBNC-20-BDBA**	5.10	+0.139<br>+0.140	-	-0.179	[+0.113] – [+0.115]	-0.069<br>-0.070
**COBNC-60-BDBA**	5.18	+0.139	-	-0.178	[+0.114] – [+0.116]	-0.069

[a] Band gaps were computed at the RI-B3LYP/def2-TZVP level of theory.
[b] Mulliken charges were computed at the B-P86/def2-SVP level of theory.
[c] Charge values for **COBOC-20-BPDBA** and **COBOC-20-TPDBA** could not be obtained due to convergence failure because of the large number of atoms in the structures.

5. Cartesian coordinates for the atoms in the calculated structures

Cartesian coordinates for COBOC-20-BDBA

6	-2.912580000	-8.963999000	-7.506793000
6	-1.885529000	-9.733424000	-6.904914000
6	-4.195739000	-8.982791000	-6.904914000
6	-4.423389000	-9.683428000	-5.712921000
6	-2.113179000	-10.434060000	-5.712921000
6	-3.377372000	-10.394482000	-5.073110000
6	8.016825000	-8.963999000	-0.752049000
6	8.052335000	-8.982791000	0.664811000
6	7.019177000	-9.733424000	-1.401504000
6	6.054834000	-10.434060000	-0.664811000
6	6.047934000	-10.394482000	0.752049000
6	7.087992000	-9.683428000	1.401504000
5	4.826707000	-10.941742000	1.561467000
8	3.733734000	-11.491252000	0.901892000
8	4.750896000	-10.752241000	2.936215000
5	3.555187000	-10.941742000	3.618829000
8	2.476453000	-11.491252000	2.936215000
5	2.526506000	-11.689122000	1.561467000
6	1.216841000	-11.964199000	0.752049000
6	1.234523000	-12.000274000	-0.664811000
6	-0.042530000	-12.000274000	1.401504000
6	-1.234523000	-12.000274000	0.664811000
6	-1.216841000	-11.964199000	-0.752049000
6	0.042530000	-12.000274000	-1.401504000
5	-2.526506000	-11.689122000	-1.561467000
8	-2.476453000	-11.491252000	-2.936215000
5	-3.555187000	-10.941742000	-3.618829000
8	-4.750896000	-10.752241000	-2.936215000
5	-4.826707000	-10.941742000	-1.561467000
8	-3.733734000	-11.491252000	-0.901892000
1	-0.881592000	-9.740350000	-7.361109000
1	-5.012011000	-8.398296000	-7.361109000
1	-5.417620000	-9.646631000	-5.237315000
1	-1.287201000	-10.988685000	-5.237315000
1	8.825416000	-8.398296000	1.190891000
1	6.978236000	-9.740350000	-2.503468000
1	5.260050000	-10.988685000	-1.190891000
1	7.107230000	-9.646631000	2.503468000
1	2.203517000	-11.981813000	-1.190891000
1	-0.079723000	-11.981813000	2.503468000
1	-2.203517000	-11.981813000	1.190891000
1	0.079723000	-11.981813000	-2.503468000
6	-2.296644000	-4.801038000	10.846570000
6	-0.583232000	-6.424143000	10.177355000
6	-1.602481000	-7.199237000	9.569517000
6	-3.304172000	-5.540056000	10.177355000
6	-2.935171000	-6.766220000	9.569517000

5	0.925707000	-6.762368000	9.943361000
8	1.290413000	-7.840996000	9.146403000
8	1.919053000	-5.906239000	10.403684000
5	-4.723733000	-4.926754000	9.943361000
8	-5.652788000	-5.585013000	9.146403000
5	-6.781095000	-4.926754000	8.671842000
8	-7.058471000	-3.650257000	9.146403000
5	-6.145335000	-2.970086000	9.943361000
8	-5.024147000	-3.650257000	10.403684000
6	4.247861000	-4.854426000	10.177355000
6	5.528040000	-4.882390000	9.569517000
6	3.856356000	-3.667841000	10.846570000
6	4.680003000	-2.534187000	10.846570000
6	5.929494000	-2.539856000	10.177355000
6	6.351687000	-3.748737000	9.569517000
5	2.590148000	-7.971655000	8.671842000
8	3.564856000	-7.101985000	9.146403000
5	3.225908000	-6.014988000	9.943361000
6	2.912580000	-8.963999000	7.506793000
6	1.885529000	-9.733424000	6.904914000
6	2.113179000	-10.434060000	5.712921000
6	3.377372000	-10.394482000	5.073110000
6	4.195739000	-8.982791000	6.904914000
6	4.423389000	-9.683428000	5.712921000
6	-7.625233000	-5.540056000	7.506793000
6	-8.674376000	-4.801038000	6.904914000
6	-7.246588000	-6.766220000	6.904914000
6	-7.842585000	-7.199237000	5.712921000
6	-8.842074000	-6.424143000	5.073110000
6	-9.270373000	-5.234055000	5.712921000
1	-2.562991000	-3.848378000	11.333959000
1	-1.330136000	-8.133435000	9.050719000
1	-3.704611000	-7.361922000	9.050719000
1	5.856816000	-5.798253000	9.050719000
1	2.868017000	-3.626763000	11.333959000
1	4.335523000	-1.606915000	11.333959000
1	7.324322000	-3.778405000	9.050719000
1	0.881592000	-9.740350000	7.361109000
1	1.287201000	-10.988685000	5.237315000
1	5.012011000	-8.398296000	7.361109000
1	5.417620000	-9.646631000	5.237315000
1	-8.991197000	-3.848378000	7.361109000
1	-6.438457000	-7.361922000	7.361109000
1	-7.500354000	-8.133435000	5.237315000
1	-10.053094000	-4.619891000	5.237315000
6	-0.963955000	-5.234055000	10.846570000
1	-0.188517000	-4.619891000	11.333959000
5	-10.336282000	-6.014988000	1.561467000
8	-10.163564000	-5.906239000	2.936215000
8	-9.775043000	-7.101985000	0.901892000
5	-8.914680000	-7.971655000	1.561467000
5	-9.307601000	-6.762368000	3.618829000

8	-8.757881000	-7.840996000	2.936215000
6	-8.016825000	-8.963999000	0.752049000
6	-7.019177000	-9.733424000	1.401504000
6	-6.054834000	-10.434060000	0.664811000
6	-6.047934000	-10.394482000	-0.752049000
6	-7.087992000	-9.683428000	-1.401504000
6	-8.052335000	-8.982791000	-0.664811000
1	-6.978236000	-9.740350000	2.503468000
1	-5.260050000	-10.988685000	1.190891000
1	-7.107230000	-9.646631000	-2.503468000
1	-8.825416000	-8.398296000	-1.190891000
6	-11.002605000	-4.854426000	0.752049000
6	-11.031451000	-4.882390000	-0.664811000
6	-11.426082000	-3.667841000	1.401504000
6	-11.794428000	-2.534187000	0.664811000
6	-11.754654000	-2.539856000	-0.752049000
6	-11.399797000	-3.748737000	-1.401504000
1	-10.714457000	-5.798253000	-1.190891000
1	-11.420017000	-3.626763000	2.503468000
1	-12.076306000	-1.606915000	1.190891000
1	-11.370746000	-3.778405000	-2.503468000
6	-6.289952000	-1.430483000	10.177355000
6	-5.275760000	-0.700636000	10.846570000
6	-7.342075000	-0.700636000	9.569517000
6	-7.342075000	0.700636000	9.569517000
6	-5.275760000	0.700636000	10.846570000
6	-6.289952000	1.430483000	10.177355000
1	-4.452032000	-1.248335000	11.333959000
1	-8.146391000	-1.248335000	9.050719000
1	-8.146391000	1.248335000	9.050719000
1	-4.452032000	1.248335000	11.333959000
6	-9.839697000	1.214549000	-6.904914000
6	-10.929405000	0.000000000	-5.073110000
6	-10.576389000	-1.214549000	-5.712921000
6	-9.425306000	0.000000000	-7.506793000
6	-9.839697000	-1.214549000	-6.904914000
5	-11.504828000	0.000000000	-3.618829000
8	-11.694097000	-1.195745000	-2.936215000
8	-11.694097000	1.195745000	-2.936215000
5	-8.381895000	0.000000000	-8.671842000
8	-7.855990000	-1.195745000	-9.146403000
5	-6.717454000	-1.209287000	-9.943361000
8	-6.210187000	0.000000000	-10.403684000
5	-6.717454000	1.209287000	-9.943361000
8	-7.855990000	1.195745000	-9.146403000
6	-5.929494000	2.539856000	-10.177355000
6	-6.351687000	3.748737000	-9.569517000
6	-4.680003000	2.534187000	-10.846570000
6	-3.856356000	3.667841000	-10.846570000
6	-5.528040000	4.882390000	-9.569517000
6	-4.247861000	4.854426000	-10.177355000
6	-11.754654000	2.539856000	-0.752049000

6	-11.794428000	2.534187000	0.664811000
6	-11.399797000	3.748737000	-1.401504000
6	-11.031451000	4.882390000	-0.664811000
6	-11.002605000	4.854426000	0.752049000
6	-11.426082000	3.667841000	1.401504000
5	-10.336282000	6.014988000	1.561467000
8	-9.775043000	7.101985000	0.901892000
8	-10.163564000	5.906239000	2.936215000
5	-9.307601000	6.762368000	3.618829000
5	-8.914680000	7.971655000	1.561467000
8	-8.757881000	7.840996000	2.936215000
6	-8.016825000	8.963999000	0.752049000
6	-7.019177000	9.733424000	1.401504000
6	-8.052335000	8.982791000	-0.664811000
6	-7.087992000	9.683428000	-1.401504000
6	-6.047934000	10.394482000	-0.752049000
6	-6.054834000	10.434060000	0.664811000
5	-4.826707000	10.941742000	-1.561467000
8	-4.750896000	10.752241000	-2.936215000
8	-3.733734000	11.491252000	-0.901892000
5	-2.526506000	11.689122000	-1.561467000
8	-2.476453000	11.491252000	-2.936215000
5	-3.555187000	10.941742000	-3.618829000
6	-3.377372000	10.394482000	-5.073110000
6	-4.423389000	9.683428000	-5.712921000
6	-2.113179000	10.434060000	-5.712921000
6	-1.885529000	9.733424000	-6.904914000
6	-2.912580000	8.963999000	-7.506793000
6	-4.195739000	8.982791000	-6.904914000
5	-2.590148000	7.971655000	-8.671842000
8	-3.564856000	7.101985000	-9.146403000
5	-3.225908000	6.014988000	-9.943361000
8	-1.919053000	5.906239000	-10.403684000
5	-0.925707000	6.762368000	-9.943361000
8	-1.290413000	7.840996000	-9.146403000
5	-11.897749000	-1.209287000	-1.561467000
8	-12.082618000	0.000000000	-0.901892000
5	-11.897749000	1.209287000	-1.561467000
6	-8.842074000	6.424143000	5.073110000
6	-7.842585000	7.199237000	5.712921000
6	-7.246588000	6.766220000	6.904914000
6	-7.625233000	5.540056000	7.506793000
6	-8.674376000	4.801038000	6.904914000
6	-9.270373000	5.234055000	5.712921000
1	-9.536050000	2.171490000	-7.361109000
1	-10.848627000	-2.171490000	-5.237315000
1	-9.536050000	-2.171490000	-7.361109000
1	-7.324322000	3.778405000	-9.050719000
1	-4.335523000	1.606915000	-11.333959000
1	-2.868017000	3.626763000	-11.333959000
1	-5.856816000	5.798253000	-9.050719000
1	-12.076306000	1.606915000	1.190891000

1	-11.370746000	3.778405000	-2.503468000
1	-10.714457000	5.798253000	-1.190891000
1	-11.420017000	3.626763000	2.503468000
1	-6.978236000	9.740350000	2.503468000
1	-8.825416000	8.398296000	-1.190891000
1	-7.107230000	9.646631000	-2.503468000
1	-5.260050000	10.988685000	1.190891000
1	-5.417620000	9.646631000	-5.237315000
1	-1.287201000	10.988685000	-5.237315000
1	-0.881592000	9.740350000	-7.361109000
1	-5.012011000	8.398296000	-7.361109000
1	-7.500354000	8.133435000	5.237315000
1	-6.438457000	7.361922000	7.361109000
1	-8.991197000	3.848378000	7.361109000
1	-10.053094000	4.619891000	5.237315000
6	-10.576389000	1.214549000	-5.712921000
1	-10.848627000	2.171490000	-5.237315000
6	8.052335000	8.982791000	0.664811000
6	6.047934000	10.394482000	0.752049000
6	6.054834000	10.434060000	-0.664811000
6	8.016825000	8.963999000	-0.752049000
6	7.019177000	9.733424000	-1.401504000
5	4.826707000	10.941742000	1.561467000
8	3.733734000	11.491252000	0.901892000
8	4.750896000	10.752241000	2.936215000
5	8.914680000	7.971655000	-1.561467000
8	8.757881000	7.840996000	-2.936215000
5	9.307601000	6.762368000	-3.618829000
8	10.163564000	5.906239000	-2.936215000
5	10.336282000	6.014988000	-1.561467000
8	9.775043000	7.101985000	-0.901892000
6	3.377372000	10.394482000	5.073110000
6	2.113179000	10.434060000	5.712921000
6	4.423389000	9.683428000	5.712921000
6	4.195739000	8.982791000	6.904914000
6	2.912580000	8.963999000	7.506793000
6	1.885529000	9.733424000	6.904914000
5	2.590148000	7.971655000	8.671842000
8	3.564856000	7.101985000	9.146403000
8	1.290413000	7.840996000	9.146403000
5	0.925707000	6.762368000	9.943361000
5	3.225908000	6.014988000	9.943361000
8	1.919053000	5.906239000	10.403684000
5	2.526506000	11.689122000	1.561467000
8	2.476453000	11.491252000	2.936215000
5	3.555187000	10.941742000	3.618829000
6	-0.583232000	6.424143000	10.177355000
6	-0.963955000	5.234055000	10.846570000
6	-2.296644000	4.801038000	10.846570000
6	-3.304172000	5.540056000	10.177355000
6	-2.935171000	6.766220000	9.569517000
6	-1.602481000	7.199237000	9.569517000

6	1.216841000	11.964199000	0.752049000
6	1.234523000	12.000274000	-0.664811000
6	0.042530000	12.000274000	-1.401504000
6	-1.216841000	11.964199000	-0.752049000
6	-0.042530000	12.000274000	1.401504000
6	-1.234523000	12.000274000	0.664811000
6	8.842074000	6.424143000	-5.073110000
6	9.270373000	5.234055000	-5.712921000
6	7.842585000	7.199237000	-5.712921000
6	7.246588000	6.766220000	-6.904914000
6	7.625233000	5.540056000	-7.506793000
6	8.674376000	4.801038000	-6.904914000
1	8.825416000	8.398296000	1.190891000
1	5.260050000	10.988685000	-1.190891000
1	6.978236000	9.740350000	-2.503468000
1	1.287201000	10.988685000	5.237315000
1	5.417620000	9.646631000	5.237315000
1	5.012011000	8.398296000	7.361109000
1	0.881592000	9.740350000	7.361109000
1	-0.188517000	4.619891000	11.333959000
1	-2.562991000	3.848378000	11.333959000
1	-1.330136000	8.133435000	9.050719000
1	2.203517000	11.981813000	-1.190891000
1	0.079723000	11.981813000	-2.503468000
1	-0.079723000	11.981813000	2.503468000
1	-2.203517000	11.981813000	1.190891000
1	10.053094000	4.619891000	-5.237315000
1	7.500354000	8.133435000	-5.237315000
1	6.438457000	7.361922000	-7.361109000
1	8.991197000	3.848378000	-7.361109000
6	7.087992000	9.683428000	1.401504000
1	7.107230000	9.646631000	2.503468000
5	6.145335000	2.970086000	-9.943361000
8	7.058471000	3.650257000	-9.146403000
8	5.024147000	3.650257000	-10.403684000
5	4.723733000	4.926754000	-9.943361000
5	6.781095000	4.926754000	-8.671842000
8	5.652788000	5.585013000	-9.146403000
6	3.304172000	5.540056000	-10.177355000
6	2.935171000	6.766220000	-9.569517000
6	1.602481000	7.199237000	-9.569517000
6	0.583232000	6.424143000	-10.177355000
6	0.963955000	5.234055000	-10.846570000
6	2.296644000	4.801038000	-10.846570000
1	3.704611000	7.361922000	-9.050719000
1	1.330136000	8.133435000	-9.050719000
1	0.188517000	4.619891000	-11.333959000
1	2.562991000	3.848378000	-11.333959000
6	-5.929494000	-2.539856000	-10.177355000
6	-6.351687000	-3.748737000	-9.569517000
6	-4.680003000	-2.534187000	-10.846570000
6	-3.856356000	-3.667841000	-10.846570000

6	-4.247861000	-4.854426000	-10.177355000
6	-5.528040000	-4.882390000	-9.569517000
5	-3.225908000	-6.014988000	-9.943361000
8	-3.564856000	-7.101985000	-9.146403000
5	-2.590148000	-7.971655000	-8.671842000
8	-1.290413000	-7.840996000	-9.146403000
5	-0.925707000	-6.762368000	-9.943361000
8	-1.919053000	-5.906239000	-10.403684000
1	-7.324322000	-3.778405000	-9.050719000
1	-4.335523000	-1.606915000	-11.333959000
1	-2.868017000	-3.626763000	-11.333959000
1	-5.856816000	-5.798253000	-9.050719000
6	6.289952000	1.430483000	-10.177355000
6	5.275760000	0.700636000	-10.846570000
6	7.342075000	0.700636000	-9.569517000
6	7.342075000	-0.700636000	-9.569517000
6	6.289952000	-1.430483000	-10.177355000
6	5.275760000	-0.700636000	-10.846570000
1	4.452032000	1.248335000	-11.333959000
1	8.146391000	1.248335000	-9.050719000
1	8.146391000	-1.248335000	-9.050719000
1	4.452032000	-1.248335000	-11.333959000
5	6.781095000	-4.926754000	-8.671842000
8	7.058471000	-3.650257000	-9.146403000
8	5.652788000	-5.585013000	-9.146403000
5	4.723733000	-4.926754000	-9.943361000
5	6.145335000	-2.970086000	-9.943361000
8	5.024147000	-3.650257000	-10.403684000
6	3.304172000	-5.540056000	-10.177355000
6	2.935171000	-6.766220000	-9.569517000
6	1.602481000	-7.199237000	-9.569517000
6	0.583232000	-6.424143000	-10.177355000
6	0.963955000	-5.234055000	-10.846570000
6	2.296644000	-4.801038000	-10.846570000
1	3.704611000	-7.361922000	-9.050719000
1	1.330136000	-8.133435000	-9.050719000
1	0.188517000	-4.619891000	-11.333959000
1	2.562991000	-3.848378000	-11.333959000
5	-6.781095000	4.926754000	8.671842000
8	-5.652788000	5.585013000	9.146403000
5	-4.723733000	4.926754000	9.943361000
8	-7.058471000	3.650257000	9.146403000
8	-5.024147000	3.650257000	10.403684000
5	-6.145335000	2.970086000	9.943361000
6	11.002605000	4.854426000	-0.752049000
6	11.031451000	4.882390000	0.664811000
6	11.426082000	3.667841000	-1.401504000
6	11.794428000	2.534187000	-0.664811000
6	11.399797000	3.748737000	1.401504000
6	11.754654000	2.539856000	0.752049000
6	4.247861000	4.854426000	10.177355000
6	3.856356000	3.667841000	10.846570000

6	5.528040000	4.882390000	9.569517000
6	6.351687000	3.748737000	9.569517000
6	5.929494000	2.539856000	10.177355000
6	4.680003000	2.534187000	10.846570000
5	6.717454000	1.209287000	9.943361000
8	7.855990000	1.195745000	9.146403000
8	6.210187000	0.000000000	10.403684000
5	6.717454000	-1.209287000	9.943361000
8	7.855990000	-1.195745000	9.146403000
5	8.381895000	0.000000000	8.671842000
6	9.425306000	0.000000000	7.506793000
6	9.839697000	1.214549000	6.904914000
6	9.839697000	-1.214549000	6.904914000
6	10.576389000	-1.214549000	5.712921000
6	10.929405000	0.000000000	5.073110000
6	10.576389000	1.214549000	5.712921000
5	11.504828000	0.000000000	3.618829000
8	11.694097000	1.195745000	2.936215000
5	11.897749000	1.209287000	1.561467000
8	12.082618000	0.000000000	0.901892000
5	11.897749000	-1.209287000	1.561467000
8	11.694097000	-1.195745000	2.936215000
1	10.714457000	5.798253000	1.190891000
1	11.420017000	3.626763000	-2.503468000
1	12.076306000	1.606915000	-1.190891000
1	11.370746000	3.778405000	2.503468000
1	2.868017000	3.626763000	11.333959000
1	5.856816000	5.798253000	9.050719000
1	7.324322000	3.778405000	9.050719000
1	4.335523000	1.606915000	11.333959000
1	9.536050000	2.171490000	7.361109000
1	9.536050000	-2.171490000	7.361109000
1	10.848627000	-2.171490000	5.237315000
1	10.848627000	2.171490000	5.237315000
1	-3.704611000	7.361922000	9.050719000
6	7.625233000	-5.540056000	-7.506793000
6	8.674376000	-4.801038000	-6.904914000
6	7.246588000	-6.766220000	-6.904914000
6	7.842585000	-7.199237000	-5.712921000
6	9.270373000	-5.234055000	-5.712921000
6	8.842074000	-6.424143000	-5.073110000
6	11.754654000	-2.539856000	0.752049000
6	11.794428000	-2.534187000	-0.664811000
6	11.399797000	-3.748737000	1.401504000
6	11.031451000	-4.882390000	0.664811000
6	11.002605000	-4.854426000	-0.752049000
6	11.426082000	-3.667841000	-1.401504000
5	10.336282000	-6.014988000	-1.561467000
8	10.163564000	-5.906239000	-2.936215000
5	9.307601000	-6.762368000	-3.618829000
8	8.757881000	-7.840996000	-2.936215000
5	8.914680000	-7.971655000	-1.561467000

8	9.775043000	-7.101985000	-0.901892000
1	8.991197000	-3.848378000	-7.361109000
1	6.438457000	-7.361922000	-7.361109000
1	7.500354000	-8.133435000	-5.237315000
1	10.053094000	-4.619891000	-5.237315000
1	12.076306000	-1.606915000	-1.190891000
1	11.370746000	-3.778405000	2.503468000
1	10.714457000	-5.798253000	1.190891000
1	11.420017000	-3.626763000	-2.503468000

Cartesian coordinates for COBOC-60-BDBA

6	16.407223000	13.689250000	3.642302000
6	16.895909000	13.142608000	2.429301000
6	16.816149000	13.082667000	4.856200000
6	17.638831000	11.950342000	4.856200000
6	18.089361000	11.373986000	3.642302000
6	17.720485000	12.007675000	2.429301000
5	18.852551000	10.009300000	3.672890000
8	19.048659000	9.365505000	4.887701000
5	19.489240000	8.049772000	4.946269000
8	19.891815000	7.431539000	3.769553000
5	19.769357000	8.075870000	2.542807000
8	19.261777000	9.370596000	2.509478000
1	16.596166000	13.601507000	1.472148000
1	16.451790000	13.495418000	5.811318000
1	17.918788000	11.476268000	5.811318000
1	18.064300000	11.580795000	1.472148000
6	20.129280000	7.331004000	1.216615000
6	19.942697000	7.953714000	-0.042780000
6	20.581425000	5.987912000	1.234676000
6	20.809057000	5.287333000	0.042780000
6	20.593986000	5.900787000	-1.216615000
6	20.170329000	7.253135000	-1.234676000
1	19.595150000	8.999611000	-0.078648000
1	20.736668000	5.486380000	2.204387000
1	21.142648000	4.236902000	0.078648000
1	20.001130000	7.750133000	-2.204387000
6	18.089361000	-11.373986000	3.642302000
6	17.638831000	-11.950342000	4.856200000
6	17.720485000	-12.007675000	2.429301000
6	16.895909000	-13.142608000	2.429301000
6	16.407223000	-13.689250000	3.642302000
6	16.816149000	-13.082667000	4.856200000
1	17.918788000	-11.476268000	5.811318000
1	18.064300000	-11.580795000	1.472148000
1	16.596166000	-13.601507000	1.472148000
1	16.451790000	-13.495418000	5.811318000
6	20.593986000	-5.900787000	-1.216615000
6	20.170329000	-7.253135000	-1.234676000
6	20.809057000	-5.287333000	0.042780000

6	20.581425000	-5.987912000	1.234676000
6	20.129280000	-7.331004000	1.216615000
6	19.942697000	-7.953714000	-0.042780000
5	19.769357000	-8.075870000	2.542807000
8	19.261777000	-9.370596000	2.509478000
8	19.891815000	-7.431539000	3.769553000
5	18.852551000	-10.009300000	3.672890000
8	19.048659000	-9.365505000	4.887701000
5	19.489240000	-8.049772000	4.946269000
1	20.001130000	-7.750133000	-2.204387000
1	21.142648000	-4.236902000	0.078648000
1	20.736668000	-5.486380000	2.204387000
1	19.595150000	-8.999611000	-0.078648000
6	19.989012000	-5.527460000	-6.304401000
6	19.407292000	-6.816218000	-6.402933000
6	19.991412000	-4.709843000	-7.462073000
6	19.396114000	-5.142352000	-8.652670000
6	18.771806000	-6.411813000	-8.738814000
6	18.810623000	-7.249724000	-7.596272000
1	19.399056000	-7.472837000	-5.516728000
1	20.443561000	-3.705816000	-7.408566000
1	19.382031000	-4.477062000	-9.531626000
1	18.336705000	-8.244681000	-7.641430000
6	18.771806000	6.411813000	-8.738814000
6	19.396114000	5.142352000	-8.652670000
6	18.810623000	7.249724000	-7.596272000
6	19.407292000	6.816218000	-6.402933000
6	19.989012000	5.527460000	-6.304401000
6	19.991412000	4.709843000	-7.462073000
5	20.498664000	4.943086000	-4.946269000
8	20.460965000	5.679874000	-3.769553000
5	20.740623000	5.086621000	-2.542807000
8	21.091003000	3.740817000	-2.509478000
5	21.135353000	2.983557000	-3.672890000
8	20.915595000	3.619668000	-4.887701000
1	19.382031000	4.477062000	-9.531626000
1	18.336705000	8.244681000	-7.641430000
1	19.399056000	7.472837000	-5.516728000
1	20.443561000	3.705816000	-7.408566000
6	21.320061000	1.430912000	-3.642302000
6	21.294349000	0.699815000	-4.856200000
6	21.394108000	0.701427000	-2.429301000
6	21.394108000	-0.701427000	-2.429301000
6	21.320061000	-1.430912000	-3.642302000
6	21.294349000	-0.699815000	-4.856200000
5	21.135353000	-2.983557000	-3.672890000
8	20.915595000	-3.619668000	-4.887701000
8	21.091003000	-3.740817000	-2.509478000
5	20.498664000	-4.943086000	-4.946269000
8	20.460965000	-5.679874000	-3.769553000
5	20.740623000	-5.086621000	-2.542807000
1	21.242185000	1.247903000	-5.811318000

1	21.421346000	1.248869000	-1.472148000
1	21.421346000	-1.248869000	-1.472148000
1	21.242185000	-1.247903000	-5.811318000
6	13.893156000	11.861813000	-11.255634000
6	13.815591000	10.903578000	-12.296991000
6	14.849074000	11.654448000	-10.230024000
6	15.672655000	10.520887000	-10.230024000
6	15.574476000	9.547675000	-11.255634000
6	14.639171000	9.770017000	-12.296991000
5	16.413685000	8.230325000	-11.196683000
8	17.278836000	7.991826000	-10.133812000
5	17.960698000	6.787025000	-10.022200000
8	17.836521000	5.856746000	-11.045848000
5	16.930513000	6.038552000	-12.082570000
8	16.259414000	7.251172000	-12.172656000
1	13.081142000	11.048445000	-13.106665000
1	14.928156000	12.390380000	-9.412636000
1	16.397006000	10.368682000	-9.412636000
1	14.549991000	9.026748000	-13.106665000
6	16.618123000	4.847070000	-13.046179000
6	15.595887000	4.914081000	-14.025744000
6	17.287917000	3.610658000	-12.869063000
6	16.920003000	2.478333000	-13.604892000
6	15.865848000	2.531806000	-14.550729000
6	15.227125000	3.779149000	-14.763268000
1	15.064571000	5.867336000	-14.185698000
1	18.089830000	3.538223000	-12.116028000
1	17.433769000	1.519074000	-13.428151000
1	14.408002000	3.846624000	-15.498836000
6	15.574476000	-9.547675000	-11.255634000
6	15.672655000	-10.520887000	-10.230024000
6	14.639171000	-9.770017000	-12.296991000
6	13.815591000	-10.903578000	-12.296991000
6	13.893156000	-11.861813000	-11.255634000
6	14.849074000	-11.654448000	-10.230024000
1	16.397006000	-10.368682000	-9.412636000
1	14.549991000	-9.026748000	-13.106665000
1	13.081142000	-11.048445000	-13.106665000
1	14.928156000	-12.390380000	-9.412636000
6	10.039019000	0.000000000	-18.963907000
6	10.667559000	-1.214933000	-18.593055000
6	10.667559000	1.214933000	-18.593055000
6	11.859456000	1.214933000	-17.856422000
6	12.472249000	0.000000000	-17.460089000
6	11.859456000	-1.214933000	-17.856422000
5	13.739490000	0.000000000	-16.545073000
8	14.292750000	1.198403000	-16.105984000
5	15.361964000	1.211055000	-15.219667000
8	15.933549000	0.000000000	-14.851792000
5	15.361964000	-1.211055000	-15.219667000
8	14.292750000	-1.198403000	-16.105984000
1	10.196781000	-2.171296000	-18.875386000

1	10.196781000	2.171296000	-18.875386000
1	12.322520000	2.171296000	-17.561607000
1	12.322520000	-2.171296000	-17.561607000
6	15.865848000	-2.531806000	-14.550729000
6	15.227125000	-3.779149000	-14.763268000
6	16.920003000	-2.478333000	-13.604892000
6	17.287917000	-3.610658000	-12.869063000
6	16.618123000	-4.847070000	-13.046179000
6	15.595887000	-4.914081000	-14.025744000
5	16.930513000	-6.038552000	-12.082570000
8	16.259414000	-7.251172000	-12.172656000
8	17.836521000	-5.856746000	-11.045848000
5	16.413685000	-8.230325000	-11.196683000
8	17.278836000	-7.991826000	-10.133812000
5	17.960698000	-6.787025000	-10.022200000
1	14.408002000	-3.846624000	-15.498836000
1	17.433769000	-1.519074000	-13.428151000
1	18.089830000	-3.538223000	-12.116028000
1	15.064571000	-5.867336000	-14.185698000
6	11.433872000	17.302601000	-6.304401000
6	10.657013000	17.557541000	-7.462073000
6	12.479792000	16.351104000	-6.402933000
6	12.707699000	15.649677000	-7.596272000
6	11.898803000	15.871689000	-8.738814000
6	10.884396000	16.857726000	-8.652670000
5	12.005006000	14.984333000	-10.022200000
8	12.940132000	13.963540000	-10.133812000
5	12.899612000	13.067032000	-11.196683000
8	11.920710000	13.222886000	-12.172656000
5	10.974821000	14.235860000	-12.082570000
8	11.081885000	15.153706000	-11.045848000
1	9.841848000	18.297822000	-7.408566000
1	13.101729000	16.140365000	-5.516728000
1	13.507511000	14.891496000	-7.641430000
1	10.247316000	17.049919000	-9.531626000
6	9.745120000	14.306947000	-13.046179000
6	8.776200000	15.326032000	-12.869063000
6	9.492963000	13.314035000	-14.025744000
6	8.299624000	13.314035000	-14.763268000
6	7.310707000	14.306947000	-14.550729000
6	7.585603000	15.326032000	-13.604892000
1	8.955115000	16.111080000	-12.116028000
1	10.235377000	12.514152000	-14.185698000
1	8.110674000	12.514152000	-15.498836000
1	6.832056000	16.111080000	-13.428151000
6	14.323899000	-7.277437000	14.550729000
6	14.540331000	-5.892884000	14.763268000
6	15.145298000	-7.940314000	13.604892000
6	16.108511000	-7.240499000	12.869063000
6	16.293380000	-5.846525000	13.046179000
6	15.505762000	-5.191457000	14.025744000
5	17.246445000	-5.066215000	12.082570000

8	17.872558000	-5.745837000	11.045848000
5	18.519824000	-5.066215000	10.022200000
8	18.676349000	-3.690722000	10.133812000
5	18.116614000	-2.989249000	11.196683000
8	17.416274000	-3.690722000	12.172656000
1	13.917307000	-5.356826000	15.498836000
1	14.997104000	-9.018356000	13.428151000
1	16.714695000	-7.770453000	12.116028000
1	15.636227000	-4.107958000	14.185698000
6	18.211998000	-1.430216000	11.255634000
6	17.586010000	-0.700579000	12.296991000
6	18.863466000	-0.700579000	10.230024000
6	18.863466000	0.700579000	10.230024000
6	18.211998000	1.430216000	11.255634000
6	17.586010000	0.700579000	12.296991000
1	17.076979000	-1.249478000	13.106665000
1	19.360015000	-1.249478000	9.412636000
1	19.360015000	1.249478000	9.412636000
1	17.076979000	1.249478000	13.106665000
6	11.975874000	-17.762601000	-1.216615000
6	11.458905000	-18.156713000	0.042780000
6	13.131116000	-16.941781000	-1.234676000
6	13.727064000	-16.508799000	-0.042780000
6	13.192489000	-16.878678000	1.216615000
6	12.054853000	-17.723731000	1.234676000
5	13.789676000	-16.306195000	2.542807000
8	14.864183000	-15.423365000	2.509478000
5	15.345169000	-14.836797000	3.672890000
8	14.793484000	-15.222251000	4.887701000
5	13.678294000	-16.047852000	4.946269000
8	13.214722000	-16.621768000	3.769553000
1	10.562971000	-18.798578000	0.078648000
1	13.551503000	-16.627282000	-2.204387000
1	14.614373000	-15.855062000	-0.078648000
1	11.625840000	-18.026358000	2.204387000
6	12.922491000	-16.221056000	6.304401000
6	11.694357000	-16.921756000	6.402933000
6	13.405016000	-15.561000000	7.462073000
6	12.669187000	-15.561000000	8.652670000
6	11.417941000	-16.221056000	8.738814000
6	10.956833000	-16.921756000	7.596272000
1	11.301742000	-17.448132000	5.516728000
1	14.360965000	-15.014492000	7.408566000
1	13.048842000	-15.014492000	9.531626000
1	9.988604000	-17.448132000	7.641430000
6	9.745120000	-14.306947000	-13.046179000
6	9.492963000	-13.314035000	-14.025744000
6	8.776200000	-15.326032000	-12.869063000
6	7.585603000	-15.326032000	-13.604892000
6	7.310707000	-14.306947000	-14.550729000
6	8.299624000	-13.314035000	-14.763268000
1	10.235377000	-12.514152000	-14.185698000

1	8.955115000	-16.111080000	-12.116028000
1	6.832056000	-16.111080000	-13.428151000
1	8.110674000	-12.514152000	-15.498836000
6	5.227383000	-20.718760000	-3.642302000
6	5.914752000	-20.468384000	-4.856200000
6	5.944046000	-20.563759000	-2.429301000
6	7.278240000	-20.130253000	-2.429301000
6	7.949140000	-19.834407000	-3.642302000
6	7.245879000	-20.035874000	-4.856200000
5	9.368715000	-19.178945000	-3.672890000
8	10.075207000	-18.902760000	-2.509478000
5	11.246869000	-18.153653000	-2.542807000
8	11.724667000	-17.704357000	-3.769553000
5	11.035589000	-17.967890000	-4.946269000
8	9.905783000	-18.773374000	-4.887701000
1	5.377370000	-20.588142000	-5.811318000
1	5.431815000	-20.758832000	-1.472148000
1	7.807305000	-19.986989000	-1.472148000
1	7.751023000	-19.816896000	-5.811318000
6	11.433872000	-17.302601000	-6.304401000
6	10.657013000	-17.557541000	-7.462073000
6	12.479792000	-16.351104000	-6.402933000
6	12.707699000	-15.649677000	-7.596272000
6	11.898803000	-15.871689000	-8.738814000
6	10.884396000	-16.857726000	-8.652670000
5	12.005006000	-14.984333000	-10.022200000
8	11.081885000	-15.153706000	-11.045848000
8	12.940132000	-13.963540000	-10.133812000
5	10.974821000	-14.235860000	-12.082570000
8	11.920710000	-13.222886000	-12.172656000
5	12.899612000	-13.067032000	-11.196683000
1	9.841848000	-18.297822000	-7.408566000
1	13.101729000	-16.140365000	-5.516728000
1	13.507511000	-14.891496000	-7.641430000
1	10.247316000	-17.049919000	-9.531626000
6	-0.578774000	-6.411813000	-20.698130000
6	-1.618054000	-7.249724000	-20.221888000
6	-0.935022000	-5.142352000	-21.218003000
6	-2.266150000	-4.709843000	-21.218003000
6	-3.300531000	-5.527460000	-20.698130000
6	-2.952247000	-6.816218000	-20.221888000
1	-1.365721000	-8.244681000	-19.818199000
1	-0.142563000	-4.477062000	-21.598488000
1	-2.516216000	-3.705816000	-21.598488000
1	-3.741210000	-7.472837000	-19.818199000
6	4.237007000	4.847070000	-20.698130000
6	3.779048000	3.610658000	-21.218003000
6	5.570314000	4.914081000	-20.221888000
6	6.394891000	3.779149000	-20.221888000
6	5.919145000	2.531806000	-20.698130000
6	4.601730000	2.478333000	-21.218003000
5	6.742805000	1.211055000	-20.546601000

8	8.013718000	1.198403000	-19.986639000
5	8.653876000	0.000000000	-19.688155000
8	8.013718000	-1.198403000	-19.986639000
5	6.742805000	-1.211055000	-20.546601000
8	6.158147000	0.000000000	-20.893323000
1	2.746887000	3.538223000	-21.598488000
1	5.950993000	5.867336000	-19.818199000
1	7.419126000	3.846624000	-19.818199000
1	4.213885000	1.519074000	-21.598488000
6	5.919145000	-2.531806000	-20.698130000
6	6.394891000	-3.779149000	-20.221888000
6	4.601730000	-2.478333000	-21.218003000
6	3.779048000	-3.610658000	-21.218003000
6	4.237007000	-4.847070000	-20.698130000
6	5.570314000	-4.914081000	-20.221888000
5	3.235423000	-6.038552000	-20.546601000
8	1.902972000	-5.856746000	-20.893323000
8	3.616124000	-7.251172000	-19.986639000
5	0.931859000	-6.787025000	-20.546601000
8	1.336626000	-7.991826000	-19.986639000
5	2.674195000	-8.230325000	-19.688155000
1	7.419126000	-3.846624000	-19.818199000
1	4.213885000	-1.519074000	-21.598488000
1	2.746887000	-3.538223000	-21.598488000
1	5.950993000	-5.867336000	-19.818199000
6	-0.578774000	6.411813000	-20.698130000
6	-1.618054000	7.249724000	-20.221888000
6	-0.935022000	5.142352000	-21.218003000
6	-2.266150000	4.709843000	-21.218003000
6	-3.300531000	5.527460000	-20.698130000
6	-2.952247000	6.816218000	-20.221888000
1	-1.365721000	8.244681000	-19.818199000
1	-0.142563000	4.477062000	-21.598488000
1	-2.516216000	3.705816000	-21.598488000
1	-3.741210000	7.472837000	-19.818199000
6	0.525445000	17.302601000	-13.046179000
6	0.145825000	16.351104000	-14.025744000
6	1.908320000	17.557541000	-12.869063000
6	2.871533000	16.857726000	-13.604892000
6	2.494926000	15.871689000	-14.550729000
6	1.111256000	15.649677000	-14.763268000
5	3.595326000	14.984333000	-15.219667000
8	4.923737000	15.153706000	-14.851792000
5	5.898890000	14.235860000	-15.219667000
8	5.556451000	13.222886000	-16.105984000
5	4.245736000	13.067032000	-16.545073000
8	3.276954000	13.963540000	-16.105984000
1	-0.924960000	16.140365000	-14.185698000
1	2.225015000	18.297822000	-12.116028000
1	3.942606000	17.049919000	-13.428151000
1	0.793960000	14.891496000	-15.498836000
6	3.854137000	11.861813000	-17.460089000

6	4.820243000	10.903578000	-17.856422000
6	2.509304000	11.654448000	-17.856422000
6	2.140988000	10.520887000	-18.593055000
6	3.102228000	9.547675000	-18.963907000
6	4.451927000	9.770017000	-18.593055000
5	2.674195000	8.230325000	-19.688155000
8	1.336626000	7.991826000	-19.986639000
8	3.616124000	7.251172000	-19.986639000
5	0.931859000	6.787025000	-20.546601000
8	1.902972000	5.856746000	-20.893323000
5	3.235423000	6.038552000	-20.546601000
1	5.872893000	11.048445000	-17.561607000
1	1.742843000	12.390380000	-17.561607000
1	1.085953000	10.368682000	-18.875386000
1	5.216004000	9.026748000	-18.875386000
6	8.121737000	-5.900787000	18.963907000
6	7.916117000	-7.253135000	18.593055000
6	9.344356000	-5.287333000	18.593055000
6	10.308621000	-5.987912000	17.856422000
6	10.090261000	-7.331004000	17.460089000
6	8.880382000	-7.953714000	17.856422000
5	11.115481000	-8.075870000	16.545073000
8	12.267481000	-7.431539000	16.105984000
5	13.139931000	-8.049772000	15.219667000
8	12.890512000	-9.365505000	14.851792000
5	11.716250000	-10.009300000	15.219667000
8	10.858674000	-9.370596000	16.105984000
1	6.973113000	-7.750133000	18.875386000
1	9.525625000	-4.236902000	18.875386000
1	11.245384000	-5.486380000	17.561607000
1	8.692872000	-8.999611000	17.561607000
6	6.988034000	-16.878678000	11.255634000
6	6.495422000	-17.723731000	10.230024000
6	6.100667000	-16.508799000	12.296991000
6	4.768086000	-16.941781000	12.296991000
6	4.267600000	-17.762601000	11.255634000
6	5.162841000	-18.156713000	10.230024000
1	7.170898000	-18.026358000	9.412636000
1	6.465401000	-15.855062000	13.106665000
1	4.088753000	-16.627282000	13.106665000
1	4.794249000	-18.798578000	9.412636000
6	-2.494926000	-15.871689000	14.550729000
6	-2.871533000	-16.857726000	13.604892000
6	-1.111256000	-15.649677000	14.763268000
6	-0.145825000	-16.351104000	14.025744000
6	-0.525445000	-17.302601000	13.046179000
6	-1.908320000	-17.557541000	12.869063000
5	0.511188000	-17.967890000	12.082570000
8	1.871839000	-17.704357000	12.172656000
5	2.755397000	-18.153653000	11.196683000
8	2.261224000	-18.902760000	10.133812000
5	0.904684000	-19.178945000	10.022200000

8	0.058308000	-18.773374000	11.045848000
1	-3.942606000	-17.049919000	13.428151000
1	-0.793960000	-14.891496000	15.498836000
1	0.924960000	-16.140365000	14.185698000
1	-2.225015000	-18.297822000	12.116028000
6	0.297189000	-19.834407000	8.738814000
6	1.082095000	-20.130253000	7.596272000
6	-1.103061000	-20.035874000	8.652670000
6	-1.698359000	-20.468384000	7.462073000
6	-0.920017000	-20.718760000	6.304401000
6	0.485426000	-20.563759000	6.402933000
1	2.174804000	-19.986989000	7.641430000
1	-1.731438000	-19.816896000	9.531626000
1	-2.792968000	-20.588142000	7.408566000
1	1.112453000	-20.758832000	5.516728000
6	-4.267600000	-17.762601000	-11.255634000
6	-4.768086000	-16.941781000	-12.296991000
6	-5.162841000	-18.156713000	-10.230024000
6	-6.495422000	-17.723731000	-10.230024000
6	-6.988034000	-16.878678000	-11.255634000
6	-6.100667000	-16.508799000	-12.296991000
1	-4.088753000	-16.627282000	-13.106665000
1	-4.794249000	-18.798578000	-9.412636000
1	-7.170898000	-18.026358000	-9.412636000
1	-6.465401000	-15.855062000	-13.106665000
6	-7.949140000	-19.834407000	3.642302000
6	-7.245879000	-20.035874000	4.856200000
6	-7.278240000	-20.130253000	2.429301000
6	-5.944046000	-20.563759000	2.429301000
6	-5.227383000	-20.718760000	3.642302000
6	-5.914752000	-20.468384000	4.856200000
5	-3.693652000	-21.022885000	3.672890000
8	-3.020765000	-21.010451000	4.887701000
5	-1.633282000	-21.022885000	4.946269000
8	-0.920905000	-21.214712000	3.769553000
5	-1.571541000	-21.297357000	2.542807000
8	-2.959750000	-21.214712000	2.509478000
1	-7.751023000	-19.816896000	5.811318000
1	-7.807305000	-19.986989000	1.472148000
1	-5.431815000	-20.758832000	1.472148000
1	-5.377370000	-20.588142000	5.811318000
6	0.920017000	-20.718760000	-6.304401000
6	-0.485426000	-20.563759000	-6.402933000
6	1.698359000	-20.468384000	-7.462073000
6	1.103061000	-20.035874000	-8.652670000
6	-0.297189000	-19.834407000	-8.738814000
6	-1.082095000	-20.130253000	-7.596272000
5	-0.904684000	-19.178945000	-10.022200000
8	-2.261224000	-18.902760000	-10.133812000
8	-0.058308000	-18.773374000	-11.045848000
5	-2.755397000	-18.153653000	-11.196683000
8	-1.871839000	-17.704357000	-12.172656000

5	-0.511188000	-17.967890000	-12.082570000
1	-1.112453000	-20.758832000	-5.516728000
1	2.792968000	-20.588142000	-7.408566000
1	1.731438000	-19.816896000	-9.531626000
1	-2.174804000	-19.986989000	-7.641430000
6	0.578774000	-6.411813000	20.698130000
6	1.618054000	-7.249724000	20.221888000
6	0.935022000	-5.142352000	21.218003000
6	2.266150000	-4.709843000	21.218003000
6	3.300531000	-5.527460000	20.698130000
6	2.952247000	-6.816218000	20.221888000
5	4.743204000	-4.943086000	20.546601000
8	5.778831000	-5.679874000	19.986639000
5	7.001133000	-5.086621000	19.688155000
8	7.187638000	-3.740817000	19.986639000
5	6.166884000	-2.983557000	20.546601000
8	4.982046000	-3.619668000	20.893323000
1	1.365721000	-8.244681000	19.818199000
1	0.142563000	-4.477062000	21.598488000
1	2.516216000	-3.705816000	21.598488000
1	3.741210000	-7.472837000	19.818199000
6	6.276847000	-1.430912000	20.698130000
6	7.394903000	-0.701427000	20.221888000
6	5.179605000	-0.699815000	21.218003000
6	5.179605000	0.699815000	21.218003000
6	6.276847000	1.430912000	20.698130000
6	7.394903000	0.701427000	20.221888000
1	8.263188000	-1.248869000	19.818199000
1	4.301994000	-1.247903000	21.598488000
1	4.301994000	1.247903000	21.598488000
1	8.263188000	1.248869000	19.818199000
6	-4.267600000	17.762601000	-11.255634000
6	-5.162841000	18.156713000	-10.230024000
6	-4.768086000	16.941781000	-12.296991000
6	-6.100667000	16.508799000	-12.296991000
6	-6.988034000	16.878678000	-11.255634000
6	-6.495422000	17.723731000	-10.230024000
1	-4.794249000	18.798578000	-9.412636000
1	-4.088753000	16.627282000	-13.106665000
1	-6.465401000	15.855062000	-13.106665000
1	-7.170898000	18.026358000	-9.412636000
6	-0.751909000	21.409488000	1.216615000
6	0.665167000	21.424465000	1.234676000
6	-1.401800000	21.424465000	-0.042780000
6	-0.665167000	21.424465000	-1.234676000
6	0.751909000	21.409488000	-1.216615000
6	1.401800000	21.424465000	0.042780000
5	1.571541000	21.297357000	-2.542807000
8	2.959750000	21.214712000	-2.509478000
5	3.693652000	21.022885000	-3.672890000
8	3.020765000	21.010451000	-4.887701000
5	1.633282000	21.022885000	-4.946269000

8	0.920905000	21.214712000	-3.769553000
1	1.190125000	21.417128000	2.204387000
1	-2.503904000	21.417128000	-0.078648000
1	-1.190125000	21.417128000	-2.204387000
1	2.503904000	21.417128000	0.078648000
6	0.920017000	20.718760000	-6.304401000
6	-0.485426000	20.563759000	-6.402933000
6	1.698359000	20.468384000	-7.462073000
6	1.103061000	20.035874000	-8.652670000
6	-0.297189000	19.834407000	-8.738814000
6	-1.082095000	20.130253000	-7.596272000
5	-0.904684000	19.178945000	-10.022200000
8	-2.261224000	18.902760000	-10.133812000
8	-0.058308000	18.773374000	-11.045848000
5	-2.755397000	18.153653000	-11.196683000
8	-1.871839000	17.704357000	-12.172656000
5	-0.511188000	17.967890000	-12.082570000
1	-1.112453000	20.758832000	-5.516728000
1	2.792968000	20.588142000	-7.408566000
1	1.731438000	19.816896000	-9.531626000
1	-2.174804000	19.986989000	-7.641430000
6	-12.922491000	16.221056000	-6.304401000
6	-11.694357000	16.921756000	-6.402933000
6	-13.405016000	15.561000000	-7.462073000
6	-12.669187000	15.561000000	-8.652670000
6	-11.417941000	16.221056000	-8.738814000
6	-10.956833000	16.921756000	-7.596272000
5	-10.541197000	16.047852000	-10.022200000
8	-9.281395000	16.621768000	-10.133812000
5	-8.441286000	16.306195000	-11.196683000
8	-8.892010000	15.423365000	-12.172656000
5	-10.147701000	14.836797000	-12.082570000
8	-10.987540000	15.222251000	-11.045848000
1	-11.301742000	17.448132000	-5.516728000
1	-14.360965000	15.014492000	-7.408566000
1	-13.048842000	15.014492000	-9.531626000
1	-9.988604000	17.448132000	-7.641430000
6	-10.595307000	13.689250000	-13.046179000
6	-11.863927000	13.082667000	-12.869063000
6	-9.728913000	13.142608000	-14.025744000
6	-10.097675000	12.007675000	-14.763268000
6	-11.347582000	11.373986000	-14.550729000
6	-12.231842000	11.950342000	-13.604892000
1	-12.555265000	13.495418000	-12.116028000
1	-8.738760000	13.601507000	-14.185698000
1	-9.395329000	11.580795000	-15.498836000
1	-13.211326000	11.476268000	-13.428151000
6	-14.323899000	7.277437000	-14.550729000
6	-14.540331000	5.892884000	-14.763268000
6	-15.145298000	7.940314000	-13.604892000
6	-16.108511000	7.240499000	-12.869063000
6	-16.293380000	5.846525000	-13.046179000

6	-15.505762000	5.191457000	-14.025744000
1	-13.917307000	5.356826000	-15.498836000
1	-14.997104000	9.018356000	-13.428151000
1	-16.714695000	7.770453000	-12.116028000
1	-15.636227000	4.107958000	-14.185698000
6	-10.090261000	-7.331004000	-17.460089000
6	-10.308621000	-5.987912000	-17.856422000
6	-8.880382000	-7.953714000	-17.856422000
6	-7.916117000	-7.253135000	-18.593055000
6	-8.121737000	-5.900787000	-18.963907000
6	-9.344356000	-5.287333000	-18.593055000
5	-7.001133000	-5.086621000	-19.688155000
8	-5.778831000	-5.679874000	-19.986639000
5	-4.743204000	-4.943086000	-20.546601000
8	-4.982046000	-3.619668000	-20.893323000
5	-6.166884000	-2.983557000	-20.546601000
8	-7.187638000	-3.740817000	-19.986639000
1	-11.245384000	-5.486380000	-17.561607000
1	-8.692872000	-8.999611000	-17.561607000
1	-6.973113000	-7.750133000	-18.875386000
1	-9.525625000	-4.236902000	-18.875386000
6	-6.276847000	-1.430912000	-20.698130000
6	-7.394903000	-0.701427000	-20.221888000
6	-5.179605000	-0.699815000	-21.218003000
6	-5.179605000	0.699815000	-21.218003000
6	-6.276847000	1.430912000	-20.698130000
6	-7.394903000	0.701427000	-20.221888000
5	-6.166884000	2.983557000	-20.546601000
8	-7.187638000	3.740817000	-19.986639000
8	-4.982046000	3.619668000	-20.893323000
5	-7.001133000	5.086621000	-19.688155000
8	-5.778831000	5.679874000	-19.986639000
5	-4.743204000	4.943086000	-20.546601000
1	-8.263188000	-1.248869000	-19.818199000
1	-4.301994000	-1.247903000	-21.598488000
1	-4.301994000	1.247903000	-21.598488000
1	-8.263188000	1.248869000	-19.818199000
6	-16.407223000	-13.689250000	-3.642302000
6	-16.816149000	-13.082667000	-4.856200000
6	-16.895909000	-13.142608000	-2.429301000
6	-17.720485000	-12.007675000	-2.429301000
6	-18.089361000	-11.373986000	-3.642302000
6	-17.638831000	-11.950342000	-4.856200000
1	-16.451790000	-13.495418000	-5.811318000
1	-16.596166000	-13.601507000	-1.472148000
1	-18.064300000	-11.580795000	-1.472148000
1	-17.918788000	-11.476268000	-5.811318000
6	-11.898803000	-15.871689000	8.738814000
6	-12.707699000	-15.649677000	7.596272000
6	-10.884396000	-16.857726000	8.652670000
6	-10.657013000	-17.557541000	7.462073000
6	-11.433872000	-17.302601000	6.304401000

6	-12.479792000	-16.351104000	6.402933000
5	-11.035589000	-17.967890000	4.946269000
8	-9.905783000	-18.773374000	4.887701000
5	-9.368715000	-19.178945000	3.672890000
8	-10.075207000	-18.902760000	2.509478000
5	-11.246869000	-18.153653000	2.542807000
8	-11.724667000	-17.704357000	3.769553000
1	-10.247316000	-17.049919000	9.531626000
1	-9.841848000	-18.297822000	7.408566000
1	-13.101729000	-16.140365000	5.516728000
6	-11.975874000	-17.762601000	1.216615000
6	-13.131116000	-16.941781000	1.234676000
6	-11.458905000	-18.156713000	-0.042780000
6	-12.054853000	-17.723731000	-1.234676000
6	-13.192489000	-16.878678000	-1.216615000
6	-13.727064000	-16.508799000	0.042780000
5	-13.789676000	-16.306195000	-2.542807000
8	-14.864183000	-15.423365000	-2.509478000
8	-13.214722000	-16.621768000	-3.769553000
5	-15.345169000	-14.836797000	-3.672890000
8	-14.793484000	-15.222251000	-4.887701000
5	-13.678294000	-16.047852000	-4.946269000
1	-13.551503000	-16.627282000	2.204387000
1	-10.562971000	-18.798578000	-0.078648000
1	-11.625840000	-18.026358000	-2.204387000
1	-14.614373000	-15.855062000	0.078648000
5	-18.852551000	-10.009300000	-3.672890000
8	-19.048659000	-9.365505000	-4.887701000
8	-19.261777000	-9.370596000	-2.509478000
5	-19.769357000	-8.075870000	-2.542807000
8	-19.891815000	-7.431539000	-3.769553000
5	-19.489240000	-8.049772000	-4.946269000
6	-19.420410000	-7.277437000	-6.304401000
6	-18.941768000	-7.940314000	-7.462073000
6	-19.707302000	-5.892884000	-6.402933000
6	-19.479394000	-5.191457000	-7.596272000
6	-18.955479000	-5.846525000	-8.738814000
6	-18.714384000	-7.240499000	-8.652670000
5	-18.519824000	-5.066215000	-10.022200000
8	-17.872558000	-5.745837000	-11.045848000
5	-17.246445000	-5.066215000	-12.082570000
8	-17.416274000	-3.690722000	-12.172656000
5	-18.116614000	-2.989249000	-11.196683000
8	-18.676349000	-3.690722000	-10.133812000
1	-18.717412000	-9.018356000	-7.408566000
1	-20.086590000	-5.356826000	-5.516728000
1	-19.680808000	-4.107958000	-7.641430000
1	-18.311944000	-7.770453000	-9.531626000
6	-21.394108000	0.701427000	2.429301000
6	-21.320061000	-1.430912000	3.642302000
6	-21.294349000	-0.699815000	4.856200000
6	-21.320061000	1.430912000	3.642302000

6	-21.294349000	0.699815000	4.856200000
5	-21.135353000	-2.983557000	3.672890000
8	-21.091003000	-3.740817000	2.509478000
8	-20.915595000	-3.619668000	4.887701000
5	-21.135353000	2.983557000	3.672890000
8	-21.091003000	3.740817000	2.509478000
5	-20.740623000	5.086621000	2.542807000
8	-20.460965000	5.679874000	3.769553000
5	-20.498664000	4.943086000	4.946269000
8	-20.915595000	3.619668000	4.887701000
6	-19.989012000	-5.527460000	6.304401000
6	-19.991412000	-4.709843000	7.462073000
6	-19.407292000	-6.816218000	6.402933000
6	-18.810623000	-7.249724000	7.596272000
6	-18.771806000	-6.411813000	8.738814000
6	-19.396114000	-5.142352000	8.652670000
5	-20.740623000	-5.086621000	2.542807000
8	-20.460965000	-5.679874000	3.769553000
5	-20.498664000	-4.943086000	4.946269000
6	-20.593986000	-5.900787000	1.216615000
6	-20.170329000	-7.253135000	1.234676000
6	-19.942697000	-7.953714000	0.042780000
6	-20.129280000	-7.331004000	-1.216615000
6	-20.809057000	-5.287333000	-0.042780000
6	-20.581425000	-5.987912000	-1.234676000
6	-20.593986000	5.900787000	1.216615000
6	-20.170329000	7.253135000	1.234676000
6	-20.809057000	5.287333000	-0.042780000
6	-20.581425000	5.987912000	-1.234676000
6	-20.129280000	7.331004000	-1.216615000
6	-19.942697000	7.953714000	0.042780000
1	-21.421346000	1.248869000	1.472148000
1	-21.242185000	-1.247903000	5.811318000
1	-21.242185000	1.247903000	5.811318000
1	-20.443561000	-3.705816000	7.408566000
1	-19.399056000	-7.472837000	5.516728000
1	-18.336705000	-8.244681000	7.641430000
1	-20.001130000	-7.750133000	2.204387000
1	-19.595150000	-8.999611000	0.078648000
1	-21.142648000	-4.236902000	-0.078648000
1	-20.736668000	-5.486380000	-2.204387000
1	-20.001130000	7.750133000	2.204387000
1	-21.142648000	4.236902000	-0.078648000
1	-20.736668000	5.486380000	-2.204387000
1	-19.595150000	8.999611000	0.078648000
6	-21.394108000	-0.701427000	2.429301000
1	-21.421346000	-1.248869000	1.472148000
5	-18.852551000	10.009300000	-3.672890000
8	-19.261777000	9.370596000	-2.509478000
8	-19.048659000	9.365505000	-4.887701000
5	-19.489240000	8.049772000	-4.946269000
5	-19.769357000	8.075870000	-2.542807000

8	-19.891815000	7.431539000	-3.769553000
6	-19.420410000	7.277437000	-6.304401000
6	-19.707302000	5.892884000	-6.402933000
6	-19.479394000	5.191457000	-7.596272000
6	-18.955479000	5.846525000	-8.738814000
6	-18.714384000	7.240499000	-8.652670000
6	-18.941768000	7.940314000	-7.462073000
1	-20.086590000	5.356826000	-5.516728000
1	-19.680808000	4.107958000	-7.641430000
1	-18.311944000	7.770453000	-9.531626000
1	-18.717412000	9.018356000	-7.408566000
6	-18.089361000	11.373986000	-3.642302000
6	-17.720485000	12.007675000	-2.429301000
6	-17.638831000	11.950342000	-4.856200000
6	-16.816149000	13.082667000	-4.856200000
6	-16.407223000	13.689250000	-3.642302000
6	-16.895909000	13.142608000	-2.429301000
1	-18.064300000	11.580795000	-1.472148000
1	-17.918788000	11.476268000	-5.811318000
1	-16.451790000	13.495418000	-5.811318000
1	-16.596166000	13.601507000	-1.472148000
6	-19.989012000	5.527460000	6.304401000
6	-19.407292000	6.816218000	6.402933000
6	-19.991412000	4.709843000	7.462073000
6	-19.396114000	5.142352000	8.652670000
6	-18.810623000	7.249724000	7.596272000
6	-18.771806000	6.411813000	8.738814000
1	-19.399056000	7.472837000	5.516728000
1	-19.382031000	4.477062000	9.531626000
1	-18.336705000	8.244681000	7.641430000
5	-13.678294000	16.047852000	-4.946269000
8	-14.793484000	15.222251000	-4.887701000
8	-13.214722000	16.621768000	-3.769553000
6	-11.433872000	17.302601000	6.304401000
6	-12.479792000	16.351104000	6.402933000
6	-10.657013000	17.557541000	7.462073000
6	-10.884396000	16.857726000	8.652670000
6	-11.898803000	15.871689000	8.738814000
6	-12.707699000	15.649677000	7.596272000
5	-12.005006000	14.984333000	10.022200000
8	-11.081885000	15.153706000	11.045848000
8	-12.940132000	13.963540000	10.133812000
5	-12.899612000	13.067032000	11.196683000
5	-10.974821000	14.235860000	12.082570000
8	-11.920710000	13.222886000	12.172656000
6	-9.745120000	14.306947000	13.046179000
6	-9.492963000	13.314035000	14.025744000
6	-8.776200000	15.326032000	12.869063000
6	-7.585603000	15.326032000	13.604892000
6	-7.310707000	14.306947000	14.550729000
6	-8.299624000	13.314035000	14.763268000
5	-5.898890000	14.235860000	15.219667000

8	-4.923737000	15.153706000	14.851792000
8	-5.556451000	13.222886000	16.105984000
5	-4.245736000	13.067032000	16.545073000
8	-3.276954000	13.963540000	16.105984000
5	-3.595326000	14.984333000	15.219667000
5	-15.345169000	14.836797000	-3.672890000
8	-14.864183000	15.423365000	-2.509478000
5	-13.789676000	16.306195000	-2.542807000
6	-13.893156000	11.861813000	11.255634000
6	-14.849074000	11.654448000	10.230024000
6	-15.672655000	10.520887000	10.230024000
6	-15.574476000	9.547675000	11.255634000
6	-14.639171000	9.770017000	12.296991000
6	-13.815591000	10.903578000	12.296991000
1	-13.101729000	16.140365000	5.516728000
1	-9.841848000	18.297822000	7.408566000
1	-10.247316000	17.049919000	9.531626000
1	-13.507511000	14.891496000	7.641430000
1	-10.235377000	12.514152000	14.185698000
1	-8.955115000	16.111080000	12.116028000
1	-6.832056000	16.111080000	13.428151000
1	-8.110674000	12.514152000	15.498836000
1	-14.928156000	12.390380000	9.412636000
1	-16.397006000	10.368682000	9.412636000
1	-14.549991000	9.026748000	13.106665000
1	-13.081142000	11.048445000	13.106665000
6	-3.779048000	-3.610658000	21.218003000
6	-5.919145000	-2.531806000	20.698130000
6	-6.394891000	-3.779149000	20.221888000
6	-4.237007000	-4.847070000	20.698130000
6	-5.570314000	-4.914081000	20.221888000
5	-6.742805000	-1.211055000	20.546601000
8	-6.158147000	0.000000000	20.893323000
8	-8.013718000	-1.198403000	19.986639000
5	-3.235423000	-6.038552000	20.546601000
8	-1.902972000	-5.856746000	20.893323000
5	-0.931859000	-6.787025000	20.546601000
8	-1.336626000	-7.991826000	19.986639000
5	-2.674195000	-8.230325000	19.688155000
8	-3.616124000	-7.251172000	19.986639000
6	-10.039019000	0.000000000	18.963907000
6	-10.667559000	1.214933000	18.593055000
6	-10.667559000	-1.214933000	18.593055000
6	-11.859456000	-1.214933000	17.856422000
6	-12.472249000	0.000000000	17.460089000
6	-11.859456000	1.214933000	17.856422000
5	-13.739490000	0.000000000	16.545073000
8	-14.292750000	-1.198403000	16.105984000
8	-14.292750000	1.198403000	16.105984000
5	-15.361964000	1.211055000	15.219667000
5	-15.361964000	-1.211055000	15.219667000
8	-15.933549000	0.000000000	14.851792000

5	-6.742805000	1.211055000	20.546601000
8	-8.013718000	1.198403000	19.986639000
5	-8.653876000	0.000000000	19.688155000
6	-15.865848000	2.531806000	14.550729000
6	-15.227125000	3.779149000	14.763268000
6	-15.595887000	4.914081000	14.025744000
6	-16.618123000	4.847070000	13.046179000
6	-17.287917000	3.610658000	12.869063000
6	-16.920003000	2.478333000	13.604892000
6	-5.919145000	2.531806000	20.698130000
6	-4.601730000	2.478333000	21.218003000
6	-3.779048000	3.610658000	21.218003000
6	-4.237007000	4.847070000	20.698130000
6	-6.394891000	3.779149000	20.221888000
6	-5.570314000	4.914081000	20.221888000
1	-2.746887000	-3.538223000	21.598488000
1	-7.419126000	-3.846624000	19.818199000
1	-5.950993000	-5.867336000	19.818199000
1	-10.196781000	2.171296000	18.875386000
1	-10.196781000	-2.171296000	18.875386000
1	-12.322520000	-2.171296000	17.561607000
1	-12.322520000	2.171296000	17.561607000
1	-14.408002000	3.846624000	15.498836000
1	-15.064571000	5.867336000	14.185698000
1	-17.433769000	1.519074000	13.428151000
1	-4.213885000	1.519074000	21.598488000
1	-2.746887000	3.538223000	21.598488000
1	-7.419126000	3.846624000	19.818199000
1	-5.950993000	5.867336000	19.818199000
6	-4.601730000	-2.478333000	21.218003000
1	-4.213885000	-1.519074000	21.598488000
5	-16.413685000	8.230325000	11.196683000
8	-16.259414000	7.251172000	12.172656000
5	-16.930513000	6.038552000	12.082570000
8	-17.278836000	7.991826000	10.133812000
8	-17.836521000	5.856746000	11.045848000
5	-17.960698000	6.787025000	10.022200000
6	-7.310707000	-14.306947000	14.550729000
6	-7.585603000	-15.326032000	13.604892000
6	-8.299624000	-13.314035000	14.763268000
6	-9.492963000	-13.314035000	14.025744000
6	-8.776200000	-15.326032000	12.869063000
6	-9.745120000	-14.306947000	13.046179000
6	-15.865848000	-2.531806000	14.550729000
6	-16.920003000	-2.478333000	13.604892000
6	-15.227125000	-3.779149000	14.763268000
6	-15.595887000	-4.914081000	14.025744000
6	-16.618123000	-4.847070000	13.046179000
6	-17.287917000	-3.610658000	12.869063000
5	-16.930513000	-6.038552000	12.082570000
8	-16.259414000	-7.251172000	12.172656000
8	-17.836521000	-5.856746000	11.045848000

5	-17.960698000	-6.787025000	10.022200000
8	-17.278836000	-7.991826000	10.133812000
5	-16.413685000	-8.230325000	11.196683000
6	-15.574476000	-9.547675000	11.255634000
6	-14.639171000	-9.770017000	12.296991000
6	-15.672655000	-10.520887000	10.230024000
6	-14.849074000	-11.654448000	10.230024000
6	-13.893156000	-11.861813000	11.255634000
6	-13.815591000	-10.903578000	12.296991000
5	-12.899612000	-13.067032000	11.196683000
8	-11.920710000	-13.222886000	12.172656000
5	-10.974821000	-14.235860000	12.082570000
8	-11.081885000	-15.153706000	11.045848000
5	-12.005006000	-14.984333000	10.022200000
8	-12.940132000	-13.963540000	10.133812000
1	-6.832056000	-16.111080000	13.428151000
1	-8.110674000	-12.514152000	15.498836000
1	-10.235377000	-12.514152000	14.185698000
1	-8.955115000	-16.111080000	12.116028000
1	-17.433769000	-1.519074000	13.428151000
1	-14.408002000	-3.846624000	15.498836000
1	-15.064571000	-5.867336000	14.185698000
1	-18.089830000	-3.538223000	12.116028000
1	-14.549991000	-9.026748000	13.106665000
1	-16.397006000	-10.368682000	9.412636000
1	-14.928156000	-12.390380000	9.412636000
1	-13.081142000	-11.048445000	13.106665000
1	-18.089830000	3.538223000	12.116028000
6	-18.211998000	-1.430216000	-11.255634000
6	-18.863466000	-0.700579000	-10.230024000
6	-17.586010000	-0.700579000	-12.296991000
6	-17.586010000	0.700579000	-12.296991000
6	-18.211998000	1.430216000	-11.255634000
6	-18.863466000	0.700579000	-10.230024000
5	-18.116614000	2.989249000	-11.196683000
8	-18.676349000	3.690722000	-10.133812000
8	-17.416274000	3.690722000	-12.172656000
5	-17.246445000	5.066215000	-12.082570000
5	-18.519824000	5.066215000	-10.022200000
8	-17.872558000	5.745837000	-11.045848000
1	-19.360015000	-1.249478000	-9.412636000
1	-17.076979000	-1.249478000	-13.106665000
1	-17.076979000	1.249478000	-13.106665000
1	-19.360015000	1.249478000	-9.412636000
6	-13.192489000	16.878678000	-1.216615000
6	-12.054853000	17.723731000	-1.234676000
6	-13.727064000	16.508799000	0.042780000
6	-13.131116000	16.941781000	1.234676000
6	-11.975874000	17.762601000	1.216615000
6	-11.458905000	18.156713000	-0.042780000
5	-11.246869000	18.153653000	2.542807000
8	-11.724667000	17.704357000	3.769553000

8	-10.075207000	18.902760000	2.509478000
5	-9.368715000	19.178945000	3.672890000
8	-9.905783000	18.773374000	4.887701000
5	-11.035589000	17.967890000	4.946269000
6	-3.854137000	11.861813000	17.460089000
6	-2.509304000	11.654448000	17.856422000
6	-4.820243000	10.903578000	17.856422000
6	-4.451927000	9.770017000	18.593055000
6	-3.102228000	9.547675000	18.963907000
6	-2.140988000	10.520887000	18.593055000
5	-2.674195000	8.230325000	19.688155000
8	-3.616124000	7.251172000	19.986639000
8	-1.336626000	7.991826000	19.986639000
5	-0.931859000	6.787025000	20.546601000
8	-1.902972000	5.856746000	20.893323000
5	-3.235423000	6.038552000	20.546601000
6	-3.102228000	-9.547675000	18.963907000
6	-2.140988000	-10.520887000	18.593055000
6	-4.451927000	-9.770017000	18.593055000
6	-2.509304000	-11.654448000	17.856422000
6	-3.854137000	-11.861813000	17.460089000
6	-4.820243000	-10.903578000	17.856422000
5	-4.245736000	-13.067032000	16.545073000
8	-5.556451000	-13.222886000	16.105984000
8	-3.276954000	-13.963540000	16.105984000
5	-3.595326000	-14.984333000	15.219667000
8	-4.923737000	-15.153706000	14.851792000
5	-5.898890000	-14.235860000	15.219667000
1	-11.625840000	18.026358000	-2.204387000
1	-14.614373000	15.855062000	0.078648000
1	-13.551503000	16.627282000	2.204387000
1	-10.562971000	18.798578000	-0.078648000
1	-1.742843000	12.390380000	17.561607000
1	-5.872893000	11.048445000	17.561607000
1	-5.216004000	9.026748000	18.875386000
1	-1.085953000	10.368682000	18.875386000
1	-1.085953000	-10.368682000	18.875386000
1	-5.216004000	-9.026748000	18.875386000
1	-1.742843000	-12.390380000	17.561607000
1	-5.872893000	-11.048445000	17.561607000
6	-14.323899000	-7.277437000	-14.550729000
6	-14.540331000	-5.892884000	-14.763268000
6	-15.145298000	-7.940314000	-13.604892000
6	-16.108511000	-7.240499000	-12.869063000
6	-16.293380000	-5.846525000	-13.046179000
6	-15.505762000	-5.191457000	-14.025744000
1	-13.917307000	-5.356826000	-15.498836000
1	-14.997104000	-9.018356000	-13.428151000
1	-16.714695000	-7.770453000	-12.116028000
1	-15.636227000	-4.107958000	-14.185698000
6	-12.922491000	-16.221056000	-6.304401000
6	-11.694357000	-16.921756000	-6.402933000

6	-13.405016000	-15.561000000	-7.462073000
6	-12.669187000	-15.561000000	-8.652670000
6	-11.417941000	-16.221056000	-8.738814000
6	-10.956833000	-16.921756000	-7.596272000
5	-10.541197000	-16.047852000	-10.022200000
8	-9.281395000	-16.621768000	-10.133812000
5	-8.441286000	-16.306195000	-11.196683000
8	-8.892010000	-15.423365000	-12.172656000
5	-10.147701000	-14.836797000	-12.082570000
8	-10.987540000	-15.222251000	-11.045848000
1	-11.301742000	-17.448132000	-5.516728000
1	-14.360965000	-15.014492000	-7.408566000
1	-13.048842000	-15.014492000	-9.531626000
1	-9.988604000	-17.448132000	-7.641430000
6	-10.595307000	-13.689250000	-13.046179000
6	-11.863927000	-13.082667000	-12.869063000
6	-9.728913000	-13.142608000	-14.025744000
6	-10.097675000	-12.007675000	-14.763268000
6	-11.347582000	-11.373986000	-14.550729000
6	-12.231842000	-11.950342000	-13.604892000
5	-11.716250000	-10.009300000	-15.219667000
8	-12.890512000	-9.365505000	-14.851792000
8	-10.858674000	-9.370596000	-16.105984000
5	-13.139931000	-8.049772000	-15.219667000
8	-12.267481000	-7.431539000	-16.105984000
5	-11.115481000	-8.075870000	-16.545073000
1	-12.555265000	-13.495418000	-12.116028000
1	-8.738760000	-13.601507000	-14.185698000
1	-9.395329000	-11.580795000	-15.498836000
1	-13.211326000	-11.476268000	-13.428151000
6	-8.121737000	5.900787000	-18.963907000
6	-9.344356000	5.287333000	-18.593055000
6	-10.308621000	5.987912000	-17.856422000
6	-10.090261000	7.331004000	-17.460089000
6	-8.880382000	7.953714000	-17.856422000
6	-7.916117000	7.253135000	-18.593055000
5	-11.115481000	8.075870000	-16.545073000
8	-10.858674000	9.370596000	-16.105984000
5	-11.716250000	10.009300000	-15.219667000
8	-12.890512000	9.365505000	-14.851792000
5	-13.139931000	8.049772000	-15.219667000
8	-12.267481000	7.431539000	-16.105984000
1	-9.525625000	4.236902000	-18.875386000
1	-11.245384000	5.486380000	-17.561607000
1	-8.692872000	8.999611000	-17.561607000
1	-6.973113000	7.750133000	-18.875386000
5	-1.571541000	21.297357000	2.542807000
8	-0.920905000	21.214712000	3.769553000
5	-1.633282000	21.022885000	4.946269000
8	-3.020765000	21.010451000	4.887701000
5	-3.693652000	21.022885000	3.672890000
8	-2.959750000	21.214712000	2.509478000

6	-5.227383000	20.718760000	3.642302000
6	-5.914752000	20.468384000	4.856200000
6	-5.944046000	20.563759000	2.429301000
6	-7.278240000	20.130253000	2.429301000
6	-7.949140000	19.834407000	3.642302000
6	-7.245879000	20.035874000	4.856200000
1	-5.377370000	20.588142000	5.811318000
1	-5.431815000	20.758832000	1.472148000
1	-7.807305000	19.986989000	1.472148000
1	-7.751023000	19.816896000	5.811318000
6	-2.494926000	15.871689000	14.550729000
6	-1.111256000	15.649677000	14.763268000
6	-2.871533000	16.857726000	13.604892000
6	-1.908320000	17.557541000	12.869063000
6	-0.525445000	17.302601000	13.046179000
6	-0.145825000	16.351104000	14.025744000
5	0.511188000	17.967890000	12.082570000
8	1.871839000	17.704357000	12.172656000
5	2.755397000	18.153653000	11.196683000
8	2.261224000	18.902760000	10.133812000
5	0.904684000	19.178945000	10.022200000
8	0.058308000	18.773374000	11.045848000
1	-0.793960000	14.891496000	15.498836000
1	-3.942606000	17.049919000	13.428151000
1	-2.225015000	18.297822000	12.116028000
1	0.924960000	16.140365000	14.185698000
6	0.297189000	19.834407000	8.738814000
6	1.082095000	20.130253000	7.596272000
6	-1.103061000	20.035874000	8.652670000
6	-1.698359000	20.468384000	7.462073000
6	-0.920017000	20.718760000	6.304401000
6	0.485426000	20.563759000	6.402933000
1	2.174804000	19.986989000	7.641430000
1	-1.731438000	19.816896000	9.531626000
1	-2.792968000	20.588142000	7.408566000
1	1.112453000	20.758832000	5.516728000
6	11.347582000	11.373986000	14.550729000
6	12.231842000	11.950342000	13.604892000
6	10.097675000	12.007675000	14.763268000
6	9.728913000	13.142608000	14.025744000
6	11.863927000	13.082667000	12.869063000
1	13.211326000	11.476268000	13.428151000
1	9.395329000	11.580795000	15.498836000
1	8.738760000	13.601507000	14.185698000
1	12.555265000	13.495418000	12.116028000
6	0.578774000	6.411813000	20.698130000
6	0.935022000	5.142352000	21.218003000
6	1.618054000	7.249724000	20.221888000
6	2.952247000	6.816218000	20.221888000
6	3.300531000	5.527460000	20.698130000
6	2.266150000	4.709843000	21.218003000
5	4.743204000	4.943086000	20.546601000

8	4.982046000	3.619668000	20.893323000
5	6.166884000	2.983557000	20.546601000
8	7.187638000	3.740817000	19.986639000
5	7.001133000	5.086621000	19.688155000
8	5.778831000	5.679874000	19.986639000
1	0.142563000	4.477062000	21.598488000
1	1.365721000	8.244681000	19.818199000
1	3.741210000	7.472837000	19.818199000
1	2.516216000	3.705816000	21.598488000
6	8.121737000	5.900787000	18.963907000
6	7.916117000	7.253135000	18.593055000
6	9.344356000	5.287333000	18.593055000
6	10.308621000	5.987912000	17.856422000
6	10.090261000	7.331004000	17.460089000
6	8.880382000	7.953714000	17.856422000
5	11.115481000	8.075870000	16.545073000
8	10.858674000	9.370596000	16.105984000
8	12.267481000	7.431539000	16.105984000
5	11.716250000	10.009300000	15.219667000
8	12.890512000	9.365505000	14.851792000
5	13.139931000	8.049772000	15.219667000
1	6.973113000	7.750133000	18.875386000
1	9.525625000	4.236902000	18.875386000
1	11.245384000	5.486380000	17.561607000
1	8.692872000	8.999611000	17.561607000
6	10.595307000	13.689250000	13.046179000
5	10.147701000	14.836797000	12.082570000
8	10.987540000	15.222251000	11.045848000
5	10.541197000	16.047852000	10.022200000
8	9.281395000	16.621768000	10.133812000
5	8.441286000	16.306195000	11.196683000
8	8.892010000	15.423365000	12.172656000
6	6.988034000	16.878678000	11.255634000
6	6.100667000	16.508799000	12.296991000
6	6.495422000	17.723731000	10.230024000
6	5.162841000	18.156713000	10.230024000
6	4.267600000	17.762601000	11.255634000
6	4.768086000	16.941781000	12.296991000
1	6.465401000	15.855062000	13.106665000
1	7.170898000	18.026358000	9.412636000
1	4.794249000	18.798578000	9.412636000
1	4.088753000	16.627282000	13.106665000
5	1.633282000	-21.022885000	-4.946269000
8	3.020765000	-21.010451000	-4.887701000
8	0.920905000	-21.214712000	-3.769553000
5	3.693652000	-21.022885000	-3.672890000
8	2.959750000	-21.214712000	-2.509478000
5	1.571541000	-21.297357000	-2.542807000
6	0.751909000	-21.409488000	-1.216615000
6	-0.665167000	-21.424465000	-1.234676000
6	1.401800000	-21.424465000	0.042780000
6	-1.401800000	-21.424465000	-0.042780000

6	0.665167000	-21.424465000	1.234676000
6	-0.751909000	-21.409488000	1.216615000
1	-1.190125000	-21.417128000	-2.204387000
1	2.503904000	-21.417128000	0.078648000
1	-2.503904000	-21.417128000	-0.078648000
1	1.190125000	-21.417128000	2.204387000
5	8.441286000	-16.306195000	11.196683000
8	8.892010000	-15.423365000	12.172656000
8	9.281395000	-16.621768000	10.133812000
5	10.541197000	-16.047852000	10.022200000
8	10.987540000	-15.222251000	11.045848000
5	10.147701000	-14.836797000	12.082570000
6	10.595307000	-13.689250000	13.046179000
6	11.863927000	-13.082667000	12.869063000
6	9.728913000	-13.142608000	14.025744000
6	10.097675000	-12.007675000	14.763268000
6	11.347582000	-11.373986000	14.550729000
6	12.231842000	-11.950342000	13.604892000
1	12.555265000	-13.495418000	12.116028000
1	8.738760000	-13.601507000	14.185698000
1	9.395329000	-11.580795000	15.498836000
1	13.211326000	-11.476268000	13.428151000
6	3.102228000	-9.547675000	-18.963907000
6	2.140988000	-10.520887000	-18.593055000
6	4.451927000	-9.770017000	-18.593055000
6	4.820243000	-10.903578000	-17.856422000
6	3.854137000	-11.861813000	-17.460089000
6	2.509304000	-11.654448000	-17.856422000
5	4.245736000	-13.067032000	-16.545073000
8	5.556451000	-13.222886000	-16.105984000
8	3.276954000	-13.963540000	-16.105984000
5	5.898890000	-14.235860000	-15.219667000
8	4.923737000	-15.153706000	-14.851792000
5	3.595326000	-14.984333000	-15.219667000
6	2.494926000	-15.871689000	-14.550729000
6	2.871533000	-16.857726000	-13.604892000
6	1.111256000	-15.649677000	-14.763268000
6	0.145825000	-16.351104000	-14.025744000
6	0.525445000	-17.302601000	-13.046179000
6	1.908320000	-17.557541000	-12.869063000
1	1.085953000	-10.368682000	-18.875386000
1	5.216004000	-9.026748000	-18.875386000
1	5.872893000	-11.048445000	-17.561607000
1	1.742843000	-12.390380000	-17.561607000
1	3.942606000	-17.049919000	-13.428151000
1	0.793960000	-14.891496000	-15.498836000
1	-0.924960000	-16.140365000	-14.185698000
1	2.225015000	-18.297822000	-12.116028000
5	18.116614000	2.989249000	11.196683000
8	18.676349000	3.690722000	10.133812000
8	17.416274000	3.690722000	12.172656000
5	17.246445000	5.066215000	12.082570000

8	17.872558000	5.745837000	11.045848000
5	18.519824000	5.066215000	10.022200000
6	15.505762000	5.191457000	14.025744000
6	16.108511000	7.240499000	12.869063000
6	15.145298000	7.940314000	13.604892000
6	14.540331000	5.892884000	14.763268000
6	14.323899000	7.277437000	14.550729000
6	16.293380000	5.846525000	13.046179000
1	15.636227000	4.107958000	14.185698000
1	16.714695000	7.770453000	12.116028000
1	14.997104000	9.018356000	13.428151000
1	13.917307000	5.356826000	15.498836000
1	-13.507511000	-14.891496000	7.641430000
6	7.949140000	19.834407000	-3.642302000
6	7.245879000	20.035874000	-4.856200000
6	7.278240000	20.130253000	-2.429301000
6	5.944046000	20.563759000	-2.429301000
6	5.227383000	20.718760000	-3.642302000
6	5.914752000	20.468384000	-4.856200000
1	7.751023000	19.816896000	-5.811318000
1	7.807305000	19.986989000	-1.472148000
1	5.431815000	20.758832000	-1.472148000
1	5.377370000	20.588142000	-5.811318000
6	11.417941000	16.221056000	8.738814000
6	10.956833000	16.921756000	7.596272000
6	12.669187000	15.561000000	8.652670000
6	13.405016000	15.561000000	7.462073000
6	12.922491000	16.221056000	6.304401000
6	11.694357000	16.921756000	6.402933000
5	13.678294000	16.047852000	4.946269000
8	14.793484000	15.222251000	4.887701000
5	15.345169000	14.836797000	3.672890000
8	14.864183000	15.423365000	2.509478000
5	13.789676000	16.306195000	2.542807000
8	13.214722000	16.621768000	3.769553000
1	13.048842000	15.014492000	9.531626000
1	14.360965000	15.014492000	7.408566000
1	11.301742000	17.448132000	5.516728000
6	13.192489000	16.878678000	1.216615000
6	12.054853000	17.723731000	1.234676000
6	13.727064000	16.508799000	-0.042780000
6	13.131116000	16.941781000	-1.234676000
6	11.975874000	17.762601000	-1.216615000
6	11.458905000	18.156713000	0.042780000
5	11.246869000	18.153653000	-2.542807000
8	10.075207000	18.902760000	-2.509478000
8	11.724667000	17.704357000	-3.769553000
5	9.368715000	19.178945000	-3.672890000
8	9.905783000	18.773374000	-4.887701000
5	11.035589000	17.967890000	-4.946269000
1	11.625840000	18.026358000	2.204387000
1	14.614373000	15.855062000	-0.078648000

1	13.551503000	16.627282000	-2.204387000
1	10.562971000	18.798578000	0.078648000
1	9.988604000	17.448132000	7.641430000
1	-19.382031000	-4.477062000	9.531626000
1	-20.443561000	3.705816000	7.408566000
6	18.955479000	5.846525000	8.738814000
6	18.714384000	7.240499000	8.652670000
6	18.941768000	7.940314000	7.462073000
6	19.420410000	7.277437000	6.304401000
6	19.707302000	5.892884000	6.402933000
6	19.479394000	5.191457000	7.596272000
1	18.311944000	7.770453000	9.531626000
1	18.717412000	9.018356000	7.408566000
1	20.086590000	5.356826000	5.516728000
1	19.680808000	4.107958000	7.641430000
6	18.955479000	-5.846525000	8.738814000
6	19.479394000	-5.191457000	7.596272000
6	18.714384000	-7.240499000	8.652670000
6	19.707302000	-5.892884000	6.402933000
6	19.420410000	-7.277437000	6.304401000
6	18.941768000	-7.940314000	7.462073000
1	19.680808000	-4.107958000	7.641430000
1	18.311944000	-7.770453000	9.531626000
1	20.086590000	-5.356826000	5.516728000
1	18.717412000	-9.018356000	7.408566000

Cartesian coordinates for COBOC-70-BDBA

6	-12.828683000	16.142807000	10.096939000
6	-11.607208000	16.852424000	10.211552000
6	-13.330353000	15.492762000	11.252127000
6	-12.619112000	15.511552000	12.457325000
6	-11.374590000	16.181508000	12.560801000
6	-10.894405000	16.871402000	11.419533000
5	-10.521878000	16.030113000	13.862680000
8	-10.980642000	15.214552000	14.888734000
5	-10.154692000	14.843827000	15.941924000
8	-8.902498000	15.436142000	16.044451000
5	-8.441297000	16.310290000	15.065729000
8	-9.267783000	16.612820000	13.988576000
1	-11.200484000	17.371648000	9.327466000
1	-14.281740000	14.939765000	11.185586000
1	-13.013007000	14.972785000	13.334844000
1	-9.930770000	17.404797000	11.478420000
6	-6.990153000	16.886483000	15.132168000
6	-6.494278000	17.720752000	14.099421000
6	-6.107255000	16.528858000	16.181517000
6	-4.774546000	16.961881000	16.181517000
6	-4.270473000	17.770161000	15.132168000
6	-5.162015000	18.153630000	14.099421000
1	-7.166807000	18.013556000	13.276103000

1	-6.474970000	15.883919000	16.996938000
1	-4.097973000	16.656252000	16.996938000
1	-4.790033000	18.785816000	13.276103000
6	4.236805000	4.846909000	24.488691000
6	3.774351000	3.607167000	24.996442000
6	5.573886000	4.916704000	24.023621000
6	6.398489000	3.781735000	24.023621000
6	5.918930000	2.531664000	24.488691000
6	4.596958000	2.474945000	24.996442000
1	2.739083000	3.532474000	25.367921000
1	5.958098000	5.872481000	23.629293000
1	7.426214000	3.851791000	23.629293000
1	4.206006000	1.513428000	25.367921000
6	3.865496000	11.896775000	21.323693000
6	4.830371000	10.934686000	21.713553000
6	2.519395000	11.685568000	21.713553000
6	2.148547000	10.544225000	22.436639000
6	3.108463000	9.566867000	22.799799000
6	4.459529000	9.793341000	22.436639000
5	2.677694000	8.241096000	23.506620000
8	3.618656000	7.258621000	23.797175000
8	1.338956000	7.999340000	23.797175000
5	3.235902000	6.039452000	24.341180000
8	1.901640000	5.852646000	24.677802000
5	0.932001000	6.788035000	24.341180000
1	5.884006000	11.082469000	21.423665000
1	1.753851000	12.424438000	21.423665000
1	1.092498000	10.388944000	22.713516000
1	5.222619000	9.046987000	22.713516000
6	7.322673000	14.341518000	18.418948000
6	8.311921000	13.350598000	18.638784000
6	7.595978000	15.352203000	17.463781000
6	8.784799000	15.344944000	16.725256000
6	9.753760000	14.327228000	16.909644000
6	9.503888000	13.343842000	17.899253000
1	8.124113000	12.557224000	19.381678000
1	6.842251000	16.135867000	17.281462000
1	8.961957000	16.122757000	15.964264000
1	10.246500000	12.545274000	18.064987000
6	0.890119000	20.600316000	10.096939000
6	1.678065000	20.369293000	11.252127000
6	-0.515178000	20.456443000	10.211552000
6	-1.103002000	20.052822000	11.419533000
6	-0.309015000	19.776931000	12.560801000
6	1.091615000	19.966437000	12.457325000
5	-0.909886000	19.153239000	13.862680000
8	-2.266977000	18.887519000	13.988576000
5	-2.757795000	18.156972000	15.065729000
8	-1.870864000	17.720859000	16.044451000
5	-0.509665000	17.977686000	15.941924000
8	-0.059363000	18.763091000	14.888734000
1	2.772797000	20.481120000	11.185586000

1	-1.149417000	20.637438000	9.327466000
1	-2.196121000	19.917937000	11.478420000
1	1.726962000	19.762091000	13.334844000
6	0.530376000	17.324087000	16.909644000
6	1.912480000	17.577896000	16.725256000
6	0.154506000	16.381640000	17.899253000
6	1.122800000	15.686486000	18.638784000
6	2.505565000	15.906691000	18.418948000
6	2.878523000	16.884997000	17.463781000
5	3.607825000	15.022731000	19.089067000
8	4.935260000	15.189169000	18.716408000
8	3.290780000	14.005777000	19.980215000
5	5.911348000	14.274270000	19.089067000
8	5.570092000	13.265184000	19.980215000
5	4.258979000	13.107788000	20.417298000
1	2.226343000	18.311291000	15.964264000
1	-0.915665000	16.172081000	18.064987000
1	0.808406000	14.934242000	19.381678000
1	3.948927000	17.075964000	17.281462000
6	-0.789366000	21.315599000	2.556757000
6	0.625146000	21.405749000	2.552796000
6	-1.446603000	21.222022000	3.808734000
6	-0.718106000	21.201727000	5.005925000
6	0.697971000	21.263257000	4.999773000
6	1.353948000	21.374775000	3.748628000
5	1.518760000	21.143869000	6.324598000
8	0.873412000	21.051301000	7.553197000
5	1.593363000	20.879332000	8.728575000
8	2.980380000	20.870689000	8.662739000
5	3.647674000	20.881745000	7.444993000
8	2.907802000	21.073464000	6.285613000
1	1.155216000	21.484238000	1.589100000
1	-2.546975000	21.152892000	3.835007000
1	-1.248084000	21.119079000	5.969445000
1	2.455141000	21.425551000	3.721935000
6	5.181359000	20.577336000	7.409885000
6	5.894363000	20.411020000	6.196229000
6	5.872412000	20.337823000	8.623862000
6	7.203391000	19.905362000	8.623862000
6	7.903247000	19.692941000	7.409885000
6	7.228657000	19.977482000	6.196229000
1	5.379219000	20.596842000	5.238792000
1	5.337965000	20.466266000	9.579508000
1	7.711265000	19.695134000	9.579508000
1	7.754640000	19.825021000	5.238792000
6	13.899924000	11.866241000	15.132168000
6	13.832632000	10.916042000	16.181517000
6	14.846594000	11.652439000	14.099421000
6	15.669978000	10.519149000	14.099421000
6	15.580779000	9.552743000	15.132168000
6	14.656292000	9.782372000	16.181517000
1	13.105629000	11.066463000	16.996938000

1	14.917244000	12.382534000	13.276103000
1	16.386171000	10.360729000	13.276103000
1	14.574694000	9.044469000	16.996938000
6	13.167605000	16.780704000	2.556757000
6	13.644318000	16.318685000	3.808734000
6	12.076230000	17.685066000	2.552796000
6	11.468411000	18.088386000	3.748628000
6	11.933558000	17.612593000	4.999773000
6	13.043023000	16.730465000	5.005925000
5	11.199352000	17.998454000	6.324598000
8	10.034210000	18.757954000	6.285613000
5	9.322952000	19.037735000	7.444993000
8	9.856305000	18.636565000	8.662739000
5	10.983505000	17.828289000	8.728575000
8	11.667039000	17.544239000	7.553197000
1	14.493904000	15.615975000	3.835007000
1	11.693529000	18.060132000	1.589100000
1	10.607372000	18.776731000	3.721935000
1	13.423204000	16.352088000	5.969445000
6	11.388441000	17.189204000	10.096939000
6	12.440783000	16.246796000	10.211552000
6	10.615187000	17.465446000	11.252127000
6	10.852842000	16.794822000	12.457325000
6	11.874587000	15.818238000	12.560801000
6	12.679101000	15.574745000	11.419533000
5	11.994104000	14.960478000	13.862680000
8	12.935828000	13.947829000	13.988576000
8	11.076694000	15.144767000	14.888734000
5	12.903503000	13.068308000	15.065729000
8	11.929620000	13.236809000	16.044451000
5	10.979346000	14.244681000	15.941924000
1	13.060279000	16.020428000	9.327466000
1	9.795261000	18.199383000	11.185586000
1	10.218724000	17.002950000	13.334844000
1	13.484169000	14.823102000	11.478420000
6	-7.889848000	19.651625000	0.000000000
6	-7.199440000	19.890223000	-1.214626000
6	-7.199440000	19.890223000	1.214626000
6	-5.866711000	20.323253000	1.214626000
6	-5.167915000	20.536035000	0.000000000
6	-5.866711000	20.323253000	-1.214626000
5	-3.651209000	20.916869000	0.000000000
8	-2.958216000	21.061737000	1.196427000
5	-1.583351000	21.261630000	1.211269000
8	-0.912338000	21.369431000	0.000000000
5	-1.583351000	21.261630000	-1.211269000
8	-2.958216000	21.061737000	-1.196427000
1	-7.718498000	19.713293000	-2.171355000
1	-7.718498000	19.713293000	2.171355000
1	-5.342787000	20.485208000	2.171355000
1	-5.342787000	20.485208000	-2.171355000
6	-6.276589000	-1.430901000	24.488691000

6	-7.399334000	-0.701449000	24.023621000
6	-5.173753000	-0.699751000	24.996442000
6	-5.173753000	0.699751000	24.996442000
6	-6.276589000	1.430901000	24.488691000
6	-7.399334000	0.701449000	24.023621000
5	-6.167801000	2.984004000	24.341180000
8	-4.978558000	3.617134000	24.677802000
5	-4.743912000	4.943819000	24.341180000
8	-5.785133000	5.684583000	23.797175000
5	-7.010295000	5.093277000	23.506620000
8	-7.194064000	3.745355000	23.797175000
1	-8.271960000	-1.248855000	23.629293000
1	-4.292301000	-1.247839000	25.367921000
1	-4.292301000	1.247839000	25.367921000
1	-8.271960000	1.248855000	23.629293000
6	-8.138063000	5.912649000	22.799799000
6	-9.364216000	5.301734000	22.436639000
6	-7.935951000	7.267573000	22.436639000
6	-8.906837000	7.972960000	21.713553000
6	-10.120001000	7.352611000	21.323693000
6	-10.335100000	6.007126000	21.713553000
1	-9.542873000	4.249387000	22.713516000
1	-6.990317000	7.762678000	22.713516000
1	-8.721796000	9.020694000	21.423665000
1	-11.274373000	5.507374000	21.423665000
6	12.509009000	0.000000000	21.323693000
6	11.892171000	-1.214952000	21.713553000
6	11.892171000	1.214952000	21.713553000
6	10.692091000	1.214955000	22.436639000
6	10.059199000	0.000000000	22.799799000
6	10.692091000	-1.214955000	22.436639000
5	8.665201000	0.000000000	23.506620000
8	8.021585000	1.198509000	23.797175000
5	6.743809000	1.211232000	24.341180000
8	6.153836000	0.000000000	24.677802000
5	6.743809000	-1.211232000	24.341180000
8	8.021585000	-1.198509000	23.797175000
1	12.358312000	-2.171351000	21.423665000
1	12.358312000	2.171351000	21.423665000
1	10.218073000	2.171333000	22.713516000
1	10.218073000	-2.171333000	22.713516000
6	5.918930000	-2.531664000	24.488691000
6	6.398489000	-3.781735000	24.023621000
6	4.596958000	-2.474945000	24.996442000
6	3.774351000	-3.607167000	24.996442000
6	4.236805000	-4.846909000	24.488691000
6	5.573886000	-4.916704000	24.023621000
1	7.426214000	-3.851791000	23.629293000
1	4.206006000	-1.513428000	25.367921000
1	2.739083000	-3.532474000	25.367921000
1	5.958098000	-5.872481000	23.629293000
6	18.713488000	6.405299000	12.560801000

6	18.730520000	7.245680000	11.419533000
6	19.326538000	5.131781000	12.457325000
6	19.890899000	4.698523000	11.252127000
6	19.867126000	5.519294000	10.096939000
6	19.296034000	6.811352000	10.211552000
1	18.264445000	8.243616000	11.478420000
1	19.328526000	4.464383000	13.334844000
1	20.335544000	3.691928000	11.185586000
1	19.272180000	7.470480000	9.327466000
6	16.640081000	-4.849020000	16.909644000
6	17.308561000	-3.612992000	16.725256000
6	15.627610000	-4.915261000	17.899253000
6	15.265699000	-3.779545000	18.638784000
6	15.902424000	-2.532503000	18.418948000
6	16.948099000	-2.480113000	17.463781000
5	15.402345000	-1.211034000	19.089067000
8	14.337193000	-1.198305000	19.980215000
5	13.782344000	0.000000000	20.417298000
8	14.337193000	1.198305000	19.980215000
5	15.402345000	1.211034000	19.089067000
8	15.970837000	0.000000000	18.716408000
1	18.103050000	-3.541122000	15.964264000
1	15.097607000	-5.868297000	18.064987000
1	14.453119000	-3.846095000	19.381678000
1	17.460493000	-1.521110000	17.281462000
6	15.902424000	2.532503000	18.418948000
6	16.948099000	2.480113000	17.463781000
6	15.265699000	3.779545000	18.638784000
6	15.627610000	4.915261000	17.899253000
6	16.640081000	4.849020000	16.909644000
6	17.308561000	3.612992000	16.725256000
5	16.940301000	6.040131000	15.941924000
8	17.826416000	5.854572000	14.888734000
8	16.275409000	7.255344000	16.044451000
5	17.934643000	6.784029000	13.862680000
8	17.262564000	7.992588000	13.988576000
5	16.416101000	8.233632000	15.065729000
1	17.460493000	1.521110000	17.281462000
1	14.453119000	3.846095000	19.381678000
1	15.097607000	5.868297000	18.064987000
1	18.103050000	3.541122000	15.964264000
5	13.778231000	16.270349000	-1.211269000
8	13.298733000	16.751974000	0.000000000
5	13.778231000	16.270349000	1.211269000
8	14.773025000	15.300507000	1.196427000
5	15.248517000	14.775976000	0.000000000
8	14.773025000	15.300507000	-1.196427000
6	16.251709000	13.576377000	0.000000000
6	16.691977000	12.993491000	1.214626000
6	16.691977000	12.993491000	-1.214626000
6	17.515649000	11.859804000	-1.214626000
6	17.933956000	11.260963000	0.000000000

6	17.515649000	11.859804000	1.214626000
5	18.764839000	9.936174000	0.000000000
8	19.116763000	9.321865000	-1.196427000
8	19.116763000	9.321865000	1.196427000
5	19.731729000	8.076062000	-1.211269000
8	20.041609000	7.471202000	0.000000000
5	19.731729000	8.076062000	1.211269000
1	16.363309000	13.432470000	2.171355000
1	16.363309000	13.432470000	-2.171355000
1	17.831579000	11.411570000	-2.171355000
1	17.831579000	11.411570000	2.171355000
6	-10.120001000	-7.352611000	21.323693000
6	-8.906837000	-7.972960000	21.713553000
6	-10.335100000	-6.007126000	21.713553000
6	-9.364216000	-5.301734000	22.436639000
6	-8.138063000	-5.912649000	22.799799000
6	-7.935951000	-7.267573000	22.436639000
5	-7.010295000	-5.093277000	23.506620000
8	-7.194064000	-3.745355000	23.797175000
5	-6.167801000	-2.984004000	24.341180000
8	-4.978558000	-3.617134000	24.677802000
5	-4.743912000	-4.943819000	24.341180000
8	-5.785133000	-5.684583000	23.797175000
1	-8.721796000	-9.020694000	21.423665000
1	-11.274373000	-5.507374000	21.423665000
1	-9.542873000	-4.249387000	22.713516000
1	-6.990317000	-7.762678000	22.713516000
6	3.108463000	-9.566867000	22.799799000
6	4.459529000	-9.793341000	22.436639000
6	2.148547000	-10.544225000	22.436639000
6	2.519395000	-11.685568000	21.713553000
6	3.865496000	-11.896775000	21.323693000
6	4.830371000	-10.934686000	21.713553000
1	5.222619000	-9.046987000	22.713516000
1	1.092498000	-10.388944000	22.713516000
1	1.753851000	-12.424438000	21.423665000
1	5.884006000	-11.082469000	21.423665000
6	0.530376000	-17.324087000	16.909644000
6	1.912480000	-17.577896000	16.725256000
6	0.154506000	-16.381640000	17.899253000
6	1.122800000	-15.686486000	18.638784000
6	2.505565000	-15.906691000	18.418948000
6	2.878523000	-16.884997000	17.463781000
5	3.607825000	-15.022731000	19.089067000
8	3.290780000	-14.005777000	19.980215000
5	4.258979000	-13.107788000	20.417298000
8	5.570092000	-13.265184000	19.980215000
5	5.911348000	-14.274270000	19.089067000
8	4.935260000	-15.189169000	18.716408000
1	2.226343000	-18.311291000	15.964264000
1	-0.915665000	-16.172081000	18.064987000
1	0.808406000	-14.934242000	19.381678000

1	3.948927000	-17.075964000	17.281462000
6	7.322673000	-14.341518000	18.418948000
6	8.311921000	-13.350598000	18.638784000
6	7.595978000	-15.352203000	17.463781000
6	8.784799000	-15.344944000	16.725256000
6	9.753760000	-14.327228000	16.909644000
6	9.503888000	-13.343842000	17.899253000
1	8.124113000	-12.557224000	19.381678000
1	6.842251000	-16.135867000	17.281462000
1	8.961957000	-16.122757000	15.964264000
1	10.246500000	-12.545274000	18.064987000
6	20.438244000	-5.906898000	4.999773000
6	20.747012000	-5.317488000	3.748628000
6	19.942134000	-7.234654000	5.005925000
6	19.736317000	-7.933766000	3.808734000
6	20.028412000	-7.337614000	2.556757000
6	20.551258000	-6.020191000	2.552796000
1	21.135590000	-4.285881000	3.721935000
1	19.699758000	-7.713153000	5.969445000
1	19.330537000	-8.958920000	3.835007000
1	20.789706000	-5.540319000	1.589100000
6	11.388441000	-17.189204000	10.096939000
6	10.615187000	-17.465446000	11.252127000
6	12.440783000	-16.246796000	10.211552000
6	12.679101000	-15.574745000	11.419533000
6	11.874587000	-15.818238000	12.560801000
6	10.852842000	-16.794822000	12.457325000
5	11.994104000	-14.960478000	13.862680000
8	11.076694000	-15.144767000	14.888734000
5	10.979346000	-14.244681000	15.941924000
8	11.929620000	-13.236809000	16.044451000
5	12.903503000	-13.068308000	15.065729000
8	12.935828000	-13.947829000	13.988576000
1	9.795261000	-18.199383000	11.185586000
1	13.060279000	-16.020428000	9.327466000
1	13.484169000	-14.823102000	11.478420000
1	10.218724000	-17.002950000	13.334844000
6	18.713488000	-6.405299000	12.560801000
6	18.730520000	-7.245680000	11.419533000
6	19.326538000	-5.131781000	12.457325000
6	19.890899000	-4.698523000	11.252127000
6	19.867126000	-5.519294000	10.096939000
6	19.296034000	-6.811352000	10.211552000
5	20.349801000	-4.936690000	8.728575000
8	20.290876000	-5.674545000	7.553197000
8	20.770192000	-3.614888000	8.662739000
5	20.578337000	-5.089388000	6.324598000
8	20.940615000	-3.746574000	6.285613000
5	20.986913000	-2.983670000	7.444993000
1	18.264445000	-8.243616000	11.478420000
1	19.328526000	-4.464383000	13.334844000
1	20.335544000	-3.691928000	11.185586000

1	19.272180000	-7.470480000	9.327466000
6	-10.611926000	-13.703734000	16.909644000
6	-9.753885000	-13.162209000	17.899253000
6	-11.879256000	-13.096689000	16.725256000
6	-12.253527000	-11.968296000	17.463781000
6	-11.376763000	-11.396049000	18.418948000
6	-10.128649000	-12.030668000	18.638784000
5	-11.748931000	-10.033018000	19.089067000
8	-10.894686000	-9.396640000	19.980215000
5	-11.150151000	-8.101059000	20.417298000
8	-12.303379000	-7.457741000	19.980215000
5	-13.172587000	-8.073524000	19.089067000
8	-12.920679000	-9.387423000	18.716408000
1	-8.764922000	-13.621703000	18.064987000
1	-12.564256000	-13.505534000	15.964264000
1	-13.231749000	-11.493624000	17.281462000
1	-9.432141000	-11.606887000	19.381678000
6	-14.353900000	-7.298372000	18.418948000
6	-14.571770000	-5.915237000	18.638784000
6	-15.169074000	-7.955390000	17.463781000
6	-16.126583000	-7.250745000	16.725256000
6	-16.312291000	-5.857855000	16.909644000
6	-15.532120000	-5.209149000	17.899253000
1	-13.953497000	-5.383774000	19.381678000
1	-15.019921000	-9.032416000	17.281462000
1	-16.727095000	-7.775878000	15.964264000
1	-15.663519000	-4.126598000	18.064987000
6	5.181359000	-20.577336000	7.409885000
6	5.894363000	-20.411020000	6.196229000
6	5.872412000	-20.337823000	8.623862000
6	7.203391000	-19.905362000	8.623862000
6	7.903247000	-19.692941000	7.409885000
6	7.228657000	-19.977482000	6.196229000
5	9.322952000	-19.037735000	7.444993000
8	9.856305000	-18.636565000	8.662739000
5	10.983505000	-17.828289000	8.728575000
8	11.667039000	-17.544239000	7.553197000
5	11.199352000	-17.998454000	6.324598000
8	10.034210000	-18.757954000	6.285613000
1	5.337965000	-20.466266000	9.579508000
1	7.711265000	-19.695134000	9.579508000
1	7.754640000	-19.825021000	5.238792000
6	11.933558000	-17.612593000	4.999773000
6	11.468411000	-18.088386000	3.748628000
6	13.043023000	-16.730465000	5.005925000
6	13.644318000	-16.318685000	3.808734000
6	13.167605000	-16.780704000	2.556757000
6	12.076230000	-17.685066000	2.552796000
5	13.778231000	-16.270349000	1.211269000
8	13.298733000	-16.751974000	0.000000000
8	14.773025000	-15.300507000	1.196427000
5	13.778231000	-16.270349000	-1.211269000

8	14.773025000	-15.300507000	-1.196427000
5	15.248517000	-14.775976000	0.000000000
1	10.607372000	-18.776731000	3.721935000
1	13.423204000	-16.352088000	5.969445000
1	14.493904000	-15.615975000	3.835007000
1	11.693529000	-18.060132000	1.589100000
5	-20.710294000	-5.064348000	-1.211269000
8	-20.945041000	-3.695004000	-1.196427000
8	-20.605465000	-5.735833000	0.000000000
5	-20.710294000	-5.064348000	1.211269000
8	-20.945041000	-3.695004000	1.196427000
5	-21.021411000	-2.991162000	0.000000000
6	-12.619112000	-15.511552000	12.457325000
6	-12.828683000	-16.142807000	10.096939000
6	-11.607208000	-16.852424000	10.211552000
6	-11.374590000	-16.181508000	12.560801000
6	-10.894405000	-16.871402000	11.419533000
5	-13.561621000	-15.955179000	8.728575000
8	-14.678661000	-15.132919000	8.662739000
8	-13.080250000	-16.517481000	7.553197000
5	-10.521878000	-16.030113000	13.862680000
8	-10.980642000	-15.214552000	14.888734000
5	-10.154692000	-14.843827000	15.941924000
8	-8.902498000	-15.436142000	16.044451000
5	-8.441297000	-16.310290000	15.065729000
8	-9.267783000	-16.612820000	13.988576000
6	-13.062899000	-16.792079000	4.999773000
6	-13.659144000	-16.496725000	3.748628000
6	-11.881102000	-17.574650000	5.005925000
6	-11.303665000	-18.019268000	3.808734000
6	-11.890384000	-17.708660000	2.556757000
6	-13.087737000	-16.950163000	2.552796000
5	-11.216314000	-18.131690000	1.211269000
8	-9.986531000	-18.778099000	1.196427000
8	-11.822540000	-17.824492000	0.000000000
5	-11.216314000	-18.131690000	-1.211269000
5	-9.340737000	-19.068230000	0.000000000
8	-9.986531000	-18.778099000	-1.196427000
5	-15.225012000	-14.749638000	7.444993000
8	-14.739133000	-15.339627000	6.285613000
5	-13.656757000	-16.213044000	6.324598000
6	-16.286862000	-13.601889000	7.409885000
6	-16.705154000	-13.001927000	8.623862000
6	-17.527744000	-11.869729000	8.623862000
6	-17.969082000	-11.286512000	7.409885000
6	-16.765936000	-13.048242000	6.196229000
6	-17.590575000	-11.913225000	6.196229000
1	-13.013007000	-14.972785000	13.334844000
1	-11.200484000	-17.371648000	9.327466000
1	-9.930770000	-17.404797000	11.478420000
1	-14.579874000	-15.890539000	3.721935000
1	-11.403762000	-17.819299000	5.969445000

1	-10.372812000	-18.610123000	3.835007000
1	-13.562707000	-16.702095000	1.589100000
1	-16.348274000	-13.419980000	9.579508000
1	-17.815054000	-11.401131000	9.579508000
1	-16.458399000	-13.501369000	5.238792000
1	-17.926490000	-11.480715000	5.238792000
6	-13.330353000	-15.492762000	11.252127000
1	-14.281740000	-14.939765000	11.185586000
6	-0.309015000	-19.776931000	12.560801000
6	1.091615000	-19.966437000	12.457325000
6	-1.103002000	-20.052822000	11.419533000
6	-0.515178000	-20.456443000	10.211552000
6	1.678065000	-20.369293000	11.252127000
6	0.890119000	-20.600316000	10.096939000
6	-7.889848000	-19.651625000	0.000000000
6	-7.199440000	-19.890223000	-1.214626000
6	-7.199440000	-19.890223000	1.214626000
6	-5.866711000	-20.323253000	1.214626000
6	-5.167915000	-20.536035000	0.000000000
6	-5.866711000	-20.323253000	-1.214626000
5	-3.651209000	-20.916869000	0.000000000
8	-2.958216000	-21.061737000	1.196427000
8	-2.958216000	-21.061737000	-1.196427000
5	-1.583351000	-21.261630000	-1.211269000
8	-0.912338000	-21.369431000	0.000000000
5	-1.583351000	-21.261630000	1.211269000
6	-0.789366000	-21.315599000	2.556757000
6	-1.446603000	-21.222022000	3.808734000
6	0.625146000	-21.405749000	2.552796000
6	1.353948000	-21.374775000	3.748628000
6	0.697971000	-21.263257000	4.999773000
6	-0.718106000	-21.201727000	5.005925000
5	1.518760000	-21.143869000	6.324598000
8	0.873412000	-21.051301000	7.553197000
5	1.593363000	-20.879332000	8.728575000
8	2.980380000	-20.870689000	8.662739000
5	3.647674000	-20.881745000	7.444993000
8	2.907802000	-21.073464000	6.285613000
1	1.726962000	-19.762091000	13.334844000
1	-2.196121000	-19.917937000	11.478420000
1	-1.149417000	-20.637438000	9.327466000
1	2.772797000	-20.481120000	11.185586000
1	-7.718498000	-19.713293000	-2.171355000
1	-7.718498000	-19.713293000	2.171355000
1	-5.342787000	-20.485208000	2.171355000
1	-5.342787000	-20.485208000	-2.171355000
1	-2.546975000	-21.152892000	3.835007000
1	1.155216000	-21.484238000	1.589100000
1	2.455141000	-21.425551000	3.721935000
1	-1.248084000	-21.119079000	5.969445000
6	-20.516267000	-5.836150000	2.556757000
6	-20.630367000	-5.182165000	3.808734000

6	-20.164897000	-7.209289000	2.552796000
6	-19.910226000	-7.892849000	3.748628000
6	-20.006874000	-7.234518000	4.999773000
6	-20.385948000	-5.868734000	5.005925000
5	-19.639692000	-7.978241000	6.324598000
8	-19.143495000	-9.277543000	6.285613000
8	-19.751078000	-7.335874000	7.553197000
5	-19.365048000	-7.967447000	8.728575000
8	-18.928217000	-9.283907000	8.662739000
5	-18.732526000	-9.921958000	7.444993000
6	-6.990153000	-16.886483000	15.132168000
6	-6.107255000	-16.528858000	16.181517000
6	-6.494278000	-17.720752000	14.099421000
6	-4.774546000	-16.961881000	16.181517000
6	-4.270473000	-17.770161000	15.132168000
6	-5.162015000	-18.153630000	14.099421000
5	-2.757795000	-18.156972000	15.065729000
8	-2.266977000	-18.887519000	13.988576000
8	-1.870864000	-17.720859000	16.044451000
5	-0.509665000	-17.977686000	15.941924000
8	-0.059363000	-18.763091000	14.888734000
5	-0.909886000	-19.153239000	13.862680000
1	-20.904654000	-4.114286000	3.835007000
1	-20.075743000	-7.737670000	1.589100000
1	-19.618230000	-8.955837000	3.721935000
1	-20.471117000	-5.339156000	5.969445000
1	-6.474970000	-15.883919000	16.996938000
1	-7.166807000	-18.013556000	13.276103000
1	-4.097973000	-16.656252000	16.996938000
1	-4.790033000	-18.785816000	13.276103000
6	16.251709000	-13.576377000	0.000000000
6	16.691977000	-12.993491000	1.214626000
6	16.691977000	-12.993491000	-1.214626000
6	17.515649000	-11.859804000	-1.214626000
6	17.933956000	-11.260963000	0.000000000
6	17.515649000	-11.859804000	1.214626000
5	18.764839000	-9.936174000	0.000000000
8	19.116763000	-9.321865000	1.196427000
5	19.731729000	-8.076062000	1.211269000
8	20.041609000	-7.471202000	0.000000000
5	19.731729000	-8.076062000	-1.211269000
8	19.116763000	-9.321865000	-1.196427000
1	16.363309000	-13.432470000	2.171355000
1	16.363309000	-13.432470000	-2.171355000
1	17.831579000	-11.411570000	-2.171355000
1	17.831579000	-11.411570000	2.171355000
6	-21.127903000	-1.431005000	0.000000000
6	-21.141476000	-0.700657000	1.214626000
6	-21.141476000	-0.700657000	-1.214626000
6	-21.141476000	0.700657000	-1.214626000
6	-21.127903000	1.431005000	0.000000000
6	-21.141476000	0.700657000	1.214626000

5	-21.021411000	2.991162000	0.000000000
8	-20.945041000	3.695004000	1.196427000
5	-20.710294000	5.064348000	1.211269000
8	-20.605465000	5.735833000	0.000000000
5	-20.710294000	5.064348000	-1.211269000
8	-20.945041000	3.695004000	-1.196427000
1	-21.133603000	-1.248985000	2.171355000
1	-21.133603000	-1.248985000	-2.171355000
1	-21.133603000	1.248985000	-2.171355000
1	-21.133603000	1.248985000	2.171355000
6	-18.904470000	5.817517000	12.560801000
6	-18.651883000	7.208156000	12.457325000
6	-19.412213000	5.147645000	11.419533000
6	-19.614432000	5.831425000	10.211552000
6	-18.853798000	7.890393000	11.252127000
1	-18.261205000	7.749261000	13.334844000
1	-19.621722000	4.066346000	11.478420000
1	-19.982559000	5.284158000	9.327466000
1	-18.621861000	8.966101000	11.185586000
6	-19.317003000	-7.212401000	10.096939000
6	-18.853798000	-7.890393000	11.252127000
6	-19.614432000	-5.831425000	10.211552000
6	-19.412213000	-5.147645000	11.419533000
6	-18.904470000	-5.817517000	12.560801000
6	-18.651883000	-7.208156000	12.457325000
5	-18.496983000	-5.053324000	13.862680000
8	-17.863104000	-5.741656000	14.888734000
5	-17.255291000	-5.070691000	15.941924000
8	-17.431667000	-3.696749000	16.044451000
5	-18.120512000	-2.987994000	15.065729000
8	-18.663633000	-3.680541000	13.988576000
1	-18.621861000	-8.966101000	11.185586000
1	-19.982559000	-5.284158000	9.327466000
1	-19.621722000	-4.066346000	11.478420000
1	-18.261205000	-7.749261000	13.334844000
6	-18.220076000	-1.429821000	15.132168000
6	-18.860279000	-0.700412000	14.099421000
6	-17.607123000	-0.700646000	16.181517000
6	-17.607123000	0.700646000	16.181517000
6	-18.220076000	1.429821000	15.132168000
6	-18.860279000	0.700412000	14.099421000
5	-18.120512000	2.987994000	15.065729000
8	-18.663633000	3.680541000	13.988576000
8	-17.431667000	3.696749000	16.044451000
5	-18.496983000	5.053324000	13.862680000
8	-17.863104000	5.741656000	14.888734000
5	-17.255291000	5.070691000	15.941924000
1	-19.346575000	-1.249544000	13.276103000
1	-17.107381000	-1.249661000	16.996938000
1	-17.107381000	1.249661000	16.996938000
1	-19.346575000	1.249544000	13.276103000
6	-19.317003000	7.212401000	10.096939000

5	-19.365048000	7.967447000	8.728575000
8	-18.928217000	9.283907000	8.662739000
5	-18.732526000	9.921958000	7.444993000
8	-19.143495000	9.277543000	6.285613000
5	-19.639692000	7.978241000	6.324598000
8	-19.751078000	7.335874000	7.553197000
6	-20.006874000	7.234518000	4.999773000
6	-20.385948000	5.868734000	5.005925000
6	-19.910226000	7.892849000	3.748628000
6	-20.164897000	7.209289000	2.552796000
6	-20.516267000	5.836150000	2.556757000
6	-20.630367000	5.182165000	3.808734000
1	-20.471117000	5.339156000	5.969445000
1	-19.618230000	8.955837000	3.721935000
1	-20.075743000	7.737670000	1.589100000
1	-20.904654000	4.114286000	3.835007000
5	17.934643000	-6.784029000	13.862680000
8	17.826416000	-5.854572000	14.888734000
8	17.262564000	-7.992588000	13.988576000
5	16.940301000	-6.040131000	15.941924000
8	16.275409000	-7.255344000	16.044451000
5	16.416101000	-8.233632000	15.065729000
6	15.580779000	-9.552743000	15.132168000
6	15.669978000	-10.519149000	14.099421000
6	14.656292000	-9.782372000	16.181517000
6	14.846594000	-11.652439000	14.099421000
6	13.832632000	-10.916042000	16.181517000
6	13.899924000	-11.866241000	15.132168000
1	16.386171000	-10.360729000	13.276103000
1	14.574694000	-9.044469000	16.996938000
1	14.917244000	-12.382534000	13.276103000
1	13.105629000	-11.066463000	16.996938000
5	2.677694000	-8.241096000	23.506620000
8	1.338956000	-7.999340000	23.797175000
8	3.618656000	-7.258621000	23.797175000
5	3.235902000	-6.039452000	24.341180000
8	1.901640000	-5.852646000	24.677802000
5	0.932001000	-6.788035000	24.341180000
6	-0.578705000	-6.411564000	24.488691000
6	-0.933274000	-5.136767000	24.996442000
6	-1.619402000	-7.253945000	24.023621000
6	-2.953638000	-6.820425000	24.023621000
6	-3.300440000	-5.527219000	24.488691000
6	-2.264281000	-4.704297000	24.996442000
1	-0.139629000	-4.467824000	25.367921000
1	-1.368445000	-8.253018000	23.629293000
1	-3.743908000	-7.481184000	23.629293000
1	-2.513159000	-3.696617000	25.367921000
6	20.028412000	7.337614000	2.556757000
6	20.551258000	6.020191000	2.552796000
6	19.736317000	7.933766000	3.808734000
6	19.942134000	7.234654000	5.005925000

6	20.438244000	5.906898000	4.999773000
6	20.747012000	5.317488000	3.748628000
5	20.578337000	5.089388000	6.324598000
8	20.290876000	5.674545000	7.553197000
8	20.940615000	3.746574000	6.285613000
5	20.349801000	4.936690000	8.728575000
8	20.770192000	3.614888000	8.662739000
5	20.986913000	2.983670000	7.444993000
6	21.171338000	1.430982000	7.409885000
6	21.157094000	0.699737000	8.623862000
6	21.233492000	0.701480000	6.196229000
6	21.233492000	-0.701480000	6.196229000
6	21.171338000	-1.430982000	7.409885000
6	21.157094000	-0.699737000	8.623862000
1	20.789706000	5.540319000	1.589100000
1	19.330537000	8.958920000	3.835007000
1	19.699758000	7.713153000	5.969445000
1	21.135590000	4.285881000	3.721935000
1	21.114098000	1.247718000	9.579508000
1	21.251031000	1.248833000	5.238792000
1	21.251031000	-1.248833000	5.238792000
1	21.114098000	-1.247718000	9.579508000
5	-11.150151000	8.101059000	20.417298000
8	-10.894686000	9.396640000	19.980215000
8	-12.303379000	7.457741000	19.980215000
5	-13.172587000	8.073524000	19.089067000
8	-12.920679000	9.387423000	18.716408000
5	-11.748931000	10.033018000	19.089067000
6	-14.571770000	5.915237000	18.638784000
6	-15.169074000	7.955390000	17.463781000
6	-16.126583000	7.250745000	16.725256000
6	-15.532120000	5.209149000	17.899253000
6	-16.312291000	5.857855000	16.909644000
6	-14.353900000	7.298372000	18.418948000
1	-13.953497000	5.383774000	19.381678000
1	-15.019921000	9.032416000	17.281462000
1	-16.727095000	7.775878000	15.964264000
1	-15.663519000	4.126598000	18.064987000
1	5.379219000	-20.596842000	5.238792000
6	-17.969082000	11.286512000	7.409885000
6	-17.590575000	11.913225000	6.196229000
6	-17.527744000	11.869729000	8.623862000
6	-16.705154000	13.001927000	8.623862000
6	-16.286862000	13.601889000	7.409885000
6	-16.765936000	13.048242000	6.196229000
5	-15.225012000	14.749638000	7.444993000
8	-14.678661000	15.132919000	8.662739000
5	-13.561621000	15.955179000	8.728575000
8	-13.080250000	16.517481000	7.553197000
5	-13.656757000	16.213044000	6.324598000
8	-14.739133000	15.339627000	6.285613000
1	-17.815054000	11.401131000	9.579508000

1	-16.348274000	13.419980000	9.579508000
1	-16.458399000	13.501369000	5.238792000
6	-13.062899000	16.792079000	4.999773000
6	-13.659144000	16.496725000	3.748628000
6	-11.881102000	17.574650000	5.005925000
6	-11.303665000	18.019268000	3.808734000
6	-11.890384000	17.708660000	2.556757000
6	-13.087737000	16.950163000	2.552796000
5	-11.216314000	18.131690000	1.211269000
8	-11.822540000	17.824492000	0.000000000
8	-9.986531000	18.778099000	1.196427000
5	-11.216314000	18.131690000	-1.211269000
8	-9.986531000	18.778099000	-1.196427000
5	-9.340737000	19.068230000	0.000000000
1	-14.579874000	15.890539000	3.721935000
1	-11.403762000	17.819299000	5.969445000
1	-10.372812000	18.610123000	3.835007000
1	-13.562707000	16.702095000	1.589100000
1	-17.926490000	11.480715000	5.238792000
6	-11.376763000	11.396049000	18.418948000
6	-12.253527000	11.968296000	17.463781000
6	-11.879256000	13.096689000	16.725256000
6	-10.611926000	13.703734000	16.909644000
6	-9.753885000	13.162209000	17.899253000
6	-10.128649000	12.030668000	18.638784000
1	-13.231749000	11.493624000	17.281462000
1	-12.564256000	13.505534000	15.964264000
1	-8.764922000	13.621703000	18.064987000
1	-9.432141000	11.606887000	19.381678000
6	-3.300440000	5.527219000	24.488691000
6	-2.953638000	6.820425000	24.023621000
6	-2.264281000	4.704297000	24.996442000
6	-1.619402000	7.253945000	24.023621000
6	-0.578705000	6.411564000	24.488691000
6	-0.933274000	5.136767000	24.996442000
1	-3.743908000	7.481184000	23.629293000
1	-2.513159000	3.696617000	25.367921000
1	-1.368445000	8.253018000	23.629293000
1	-0.139629000	4.467824000	25.367921000
6	-12.828683000	-16.142807000	-10.096939000
6	-11.607208000	-16.852424000	-10.211552000
6	-13.330353000	-15.492762000	-11.252127000
6	-12.619112000	-15.511552000	-12.457325000
6	-11.374590000	-16.181508000	-12.560801000
6	-10.894405000	-16.871402000	-11.419533000
5	-10.521878000	-16.030113000	-13.862680000
8	-10.980642000	-15.214552000	-14.888734000
5	-10.154692000	-14.843827000	-15.941924000
8	-8.902498000	-15.436142000	-16.044451000
5	-8.441297000	-16.310290000	-15.065729000
8	-9.267783000	-16.612820000	-13.988576000
1	-11.200484000	-17.371648000	-9.327466000

1	-14.281740000	-14.939765000	-11.185586000
1	-13.013007000	-14.972785000	-13.334844000
1	-9.930770000	-17.404797000	-11.478420000
6	-6.990153000	-16.886483000	-15.132168000
6	-6.494278000	-17.720752000	-14.099421000
6	-6.107255000	-16.528858000	-16.181517000
6	-4.774546000	-16.961881000	-16.181517000
6	-4.270473000	-17.770161000	-15.132168000
6	-5.162015000	-18.153630000	-14.099421000
1	-7.166807000	-18.013556000	-13.276103000
1	-6.474970000	-15.883919000	-16.996938000
1	-4.097973000	-16.656252000	-16.996938000
1	-4.790033000	-18.785816000	-13.276103000
6	4.236805000	-4.846909000	-24.488691000
6	3.774351000	-3.607167000	-24.996442000
6	5.573886000	-4.916704000	-24.023621000
6	6.398489000	-3.781735000	-24.023621000
6	5.918930000	-2.531664000	-24.488691000
6	4.596958000	-2.474945000	-24.996442000
1	2.739083000	-3.532474000	-25.367921000
1	5.958098000	-5.872481000	-23.629293000
1	7.426214000	-3.851791000	-23.629293000
1	4.206006000	-1.513428000	-25.367921000
6	3.865496000	-11.896775000	-21.323693000
6	4.830371000	-10.934686000	-21.713553000
6	2.519395000	-11.685568000	-21.713553000
6	2.148547000	-10.544225000	-22.436639000
6	3.108463000	-9.566867000	-22.799799000
6	4.459529000	-9.793341000	-22.436639000
5	2.677694000	-8.241096000	-23.506620000
8	3.618656000	-7.258621000	-23.797175000
8	1.338956000	-7.999340000	-23.797175000
5	3.235902000	-6.039452000	-24.341180000
8	1.901640000	-5.852646000	-24.677802000
5	0.932001000	-6.788035000	-24.341180000
1	5.884006000	-11.082469000	-21.423665000
1	1.753851000	-12.424438000	-21.423665000
1	1.092498000	-10.388944000	-22.713516000
1	5.222619000	-9.046987000	-22.713516000
6	7.322673000	-14.341518000	-18.418948000
6	8.311921000	-13.350598000	-18.638784000
6	7.595978000	-15.352203000	-17.463781000
6	8.784799000	-15.344944000	-16.725256000
6	9.753760000	-14.327228000	-16.909644000
6	9.503888000	-13.343842000	-17.899253000
1	8.124113000	-12.557224000	-19.381678000
1	6.842251000	-16.135867000	-17.281462000
1	8.961957000	-16.122757000	-15.964264000
1	10.246500000	-12.545274000	-18.064987000
6	0.890119000	-20.600316000	-10.096939000
6	1.678065000	-20.369293000	-11.252127000
6	-0.515178000	-20.456443000	-10.211552000

6	-1.103002000	-20.052822000	-11.419533000
6	-0.309015000	-19.776931000	-12.560801000
6	1.091615000	-19.966437000	-12.457325000
5	-0.909886000	-19.153239000	-13.862680000
8	-2.266977000	-18.887519000	-13.988576000
5	-2.757795000	-18.156972000	-15.065729000
8	-1.870864000	-17.720859000	-16.044451000
5	-0.509665000	-17.977686000	-15.941924000
8	-0.059363000	-18.763091000	-14.888734000
1	2.772797000	-20.481120000	-11.185586000
1	-1.149417000	-20.637438000	-9.327466000
1	-2.196121000	-19.917937000	-11.478420000
1	1.726962000	-19.762091000	-13.334844000
6	0.530376000	-17.324087000	-16.909644000
6	1.912480000	-17.577896000	-16.725256000
6	0.154506000	-16.381640000	-17.899253000
6	1.122800000	-15.686486000	-18.638784000
6	2.505565000	-15.906691000	-18.418948000
6	2.878523000	-16.884997000	-17.463781000
5	3.607825000	-15.022731000	-19.089067000
8	4.935260000	-15.189169000	-18.716408000
8	3.290780000	-14.005777000	-19.980215000
5	5.911348000	-14.274270000	-19.089067000
8	5.570092000	-13.265184000	-19.980215000
5	4.258979000	-13.107788000	-20.417298000
1	2.226343000	-18.311291000	-15.964264000
1	-0.915665000	-16.172081000	-18.064987000
1	0.808406000	-14.934242000	-19.381678000
1	3.948927000	-17.075964000	-17.281462000
6	-0.789366000	-21.315599000	-2.556757000
6	0.625146000	-21.405749000	-2.552796000
6	-1.446603000	-21.222022000	-3.808734000
6	-0.718106000	-21.201727000	-5.005925000
6	0.697971000	-21.263257000	-4.999773000
6	1.353948000	-21.374775000	-3.748628000
5	1.518760000	-21.143869000	-6.324598000
8	0.873412000	-21.051301000	-7.553197000
5	1.593363000	-20.879332000	-8.728575000
8	2.980380000	-20.870689000	-8.662739000
5	3.647674000	-20.881745000	-7.444993000
8	2.907802000	-21.073464000	-6.285613000
1	1.155216000	-21.484238000	-1.589100000
1	-2.546975000	-21.152892000	-3.835007000
1	-1.248084000	-21.119079000	-5.969445000
1	2.455141000	-21.425551000	-3.721935000
6	5.181359000	-20.577336000	-7.409885000
6	5.894363000	-20.411020000	-6.196229000
6	5.872412000	-20.337823000	-8.623862000
6	7.203391000	-19.905362000	-8.623862000
6	7.903247000	-19.692941000	-7.409885000
6	7.228657000	-19.977482000	-6.196229000
1	5.379219000	-20.596842000	-5.238792000

1	5.337965000	-20.466266000	-9.579508000
1	7.711265000	-19.695134000	-9.579508000
1	7.754640000	-19.825021000	-5.238792000
6	13.899924000	-11.866241000	-15.132168000
6	13.832632000	-10.916042000	-16.181517000
6	14.846594000	-11.652439000	-14.099421000
6	15.669978000	-10.519149000	-14.099421000
6	15.580779000	-9.552743000	-15.132168000
6	14.656292000	-9.782372000	-16.181517000
1	13.105629000	-11.066463000	-16.996938000
1	14.917244000	-12.382534000	-13.276103000
1	16.386171000	-10.360729000	-13.276103000
1	14.574694000	-9.044469000	-16.996938000
6	13.167605000	-16.780704000	-2.556757000
6	13.644318000	-16.318685000	-3.808734000
6	12.076230000	-17.685066000	-2.552796000
6	11.468411000	-18.088386000	-3.748628000
6	11.933558000	-17.612593000	-4.999773000
6	13.043023000	-16.730465000	-5.005925000
5	11.199352000	-17.998454000	-6.324598000
8	10.034210000	-18.757954000	-6.285613000
5	9.322952000	-19.037735000	-7.444993000
8	9.856305000	-18.636565000	-8.662739000
5	10.983505000	-17.828289000	-8.728575000
8	11.667039000	-17.544239000	-7.553197000
1	14.493904000	-15.615975000	-3.835007000
1	11.693529000	-18.060132000	-1.589100000
1	10.607372000	-18.776731000	-3.721935000
1	13.423204000	-16.352088000	-5.969445000
6	11.388441000	-17.189204000	-10.096939000
6	12.440783000	-16.246796000	-10.211552000
6	10.615187000	-17.465446000	-11.252127000
6	10.852842000	-16.794822000	-12.457325000
6	11.874587000	-15.818238000	-12.560801000
6	12.679101000	-15.574745000	-11.419533000
5	11.994104000	-14.960478000	-13.862680000
8	12.935828000	-13.947829000	-13.988576000
8	11.076694000	-15.144767000	-14.888734000
5	12.903503000	-13.068308000	-15.065729000
8	11.929620000	-13.236809000	-16.044451000
5	10.979346000	-14.244681000	-15.941924000
1	13.060279000	-16.020428000	-9.327466000
1	9.795261000	-18.199383000	-11.185586000
1	10.218724000	-17.002950000	-13.334844000
1	13.484169000	-14.823102000	-11.478420000
6	-6.276589000	1.430901000	-24.488691000
6	-7.399334000	0.701449000	-24.023621000
6	-5.173753000	0.699751000	-24.996442000
6	-5.173753000	-0.699751000	-24.996442000
6	-6.276589000	-1.430901000	-24.488691000
6	-7.399334000	-0.701449000	-24.023621000
5	-6.167801000	-2.984004000	-24.341180000

8	-4.978558000	-3.617134000	-24.677802000
5	-4.743912000	-4.943819000	-24.341180000
8	-5.785133000	-5.684583000	-23.797175000
5	-7.010295000	-5.093277000	-23.506620000
8	-7.194064000	-3.745355000	-23.797175000
1	-8.271960000	1.248855000	-23.629293000
1	-4.292301000	1.247839000	-25.367921000
1	-4.292301000	-1.247839000	-25.367921000
1	-8.271960000	-1.248855000	-23.629293000
6	-8.138063000	-5.912649000	-22.799799000
6	-9.364216000	-5.301734000	-22.436639000
6	-7.935951000	-7.267573000	-22.436639000
6	-8.906837000	-7.972960000	-21.713553000
6	-10.120001000	-7.352611000	-21.323693000
6	-10.335100000	-6.007126000	-21.713553000
1	-9.542873000	-4.249387000	-22.713516000
1	-6.990317000	-7.762678000	-22.713516000
1	-8.721796000	-9.020694000	-21.423665000
1	-11.274373000	-5.507374000	-21.423665000
6	12.509009000	0.000000000	-21.323693000
6	11.892171000	1.214952000	-21.713553000
6	11.892171000	-1.214952000	-21.713553000
6	10.692091000	-1.214955000	-22.436639000
6	10.059199000	0.000000000	-22.799799000
6	10.692091000	1.214955000	-22.436639000
5	8.665201000	0.000000000	-23.506620000
8	8.021585000	-1.198509000	-23.797175000
5	6.743809000	-1.211232000	-24.341180000
8	6.153836000	0.000000000	-24.677802000
5	6.743809000	1.211232000	-24.341180000
8	8.021585000	1.198509000	-23.797175000
1	12.358312000	2.171351000	-21.423665000
1	12.358312000	-2.171351000	-21.423665000
1	10.218073000	-2.171333000	-22.713516000
1	10.218073000	2.171333000	-22.713516000
6	5.918930000	2.531664000	-24.488691000
6	6.398489000	3.781735000	-24.023621000
6	4.596958000	2.474945000	-24.996442000
6	3.774351000	3.607167000	-24.996442000
6	4.236805000	4.846909000	-24.488691000
6	5.573886000	4.916704000	-24.023621000
1	7.426214000	3.851791000	-23.629293000
1	4.206006000	1.513428000	-25.367921000
1	2.739083000	3.532474000	-25.367921000
1	5.958098000	5.872481000	-23.629293000
6	18.713488000	-6.405299000	-12.560801000
6	18.730520000	-7.245680000	-11.419533000
6	19.326538000	-5.131781000	-12.457325000
6	19.890899000	-4.698523000	-11.252127000
6	19.867126000	-5.519294000	-10.096939000
6	19.296034000	-6.811352000	-10.211552000
1	18.264445000	-8.243616000	-11.478420000

1	19.328526000	-4.464383000	-13.334844000
1	20.335544000	-3.691928000	-11.185586000
1	19.272180000	-7.470480000	-9.327466000
6	16.640081000	4.849020000	-16.909644000
6	17.308561000	3.612992000	-16.725256000
6	15.627610000	4.915261000	-17.899253000
6	15.265699000	3.779545000	-18.638784000
6	15.902424000	2.532503000	-18.418948000
6	16.948099000	2.480113000	-17.463781000
5	15.402345000	1.211034000	-19.089067000
8	14.337193000	1.198305000	-19.980215000
5	13.782344000	0.000000000	-20.417298000
8	14.337193000	-1.198305000	-19.980215000
5	15.402345000	-1.211034000	-19.089067000
8	15.970837000	0.000000000	-18.716408000
1	18.103050000	3.541122000	-15.964264000
1	15.097607000	5.868297000	-18.064987000
1	14.453119000	3.846095000	-19.381678000
1	17.460493000	1.521110000	-17.281462000
6	15.902424000	-2.532503000	-18.418948000
6	16.948099000	-2.480113000	-17.463781000
6	15.265699000	-3.779545000	-18.638784000
6	15.627610000	-4.915261000	-17.899253000
6	16.640081000	-4.849020000	-16.909644000
6	17.308561000	-3.612992000	-16.725256000
5	16.940301000	-6.040131000	-15.941924000
8	17.826416000	-5.854572000	-14.888734000
8	16.275409000	-7.255344000	-16.044451000
5	17.934643000	-6.784029000	-13.862680000
8	17.262564000	-7.992588000	-13.988576000
5	16.416101000	-8.233632000	-15.065729000
1	17.460493000	-1.521110000	-17.281462000
1	14.453119000	-3.846095000	-19.381678000
1	15.097607000	-5.868297000	-18.064987000
1	18.103050000	-3.541122000	-15.964264000
6	-10.120001000	7.352611000	-21.323693000
6	-8.906837000	7.972960000	-21.713553000
6	-10.335100000	6.007126000	-21.713553000
6	-9.364216000	5.301734000	-22.436639000
6	-8.138063000	5.912649000	-22.799799000
6	-7.935951000	7.267573000	-22.436639000
5	-7.010295000	5.093277000	-23.506620000
8	-7.194064000	3.745355000	-23.797175000
5	-6.167801000	2.984004000	-24.341180000
8	-4.978558000	3.617134000	-24.677802000
5	-4.743912000	4.943819000	-24.341180000
8	-5.785133000	5.684583000	-23.797175000
1	-8.721796000	9.020694000	-21.423665000
1	-11.274373000	5.507374000	-21.423665000
1	-9.542873000	4.249387000	-22.713516000
1	-6.990317000	7.762678000	-22.713516000
6	3.108463000	9.566867000	-22.799799000

6	4.459529000	9.793341000	-22.436639000
6	2.148547000	10.544225000	-22.436639000
6	2.519395000	11.685568000	-21.713553000
6	3.865496000	11.896775000	-21.323693000
6	4.830371000	10.934686000	-21.713553000
1	5.222619000	9.046987000	-22.713516000
1	1.092498000	10.388944000	-22.713516000
1	1.753851000	12.424438000	-21.423665000
1	5.884006000	11.082469000	-21.423665000
6	0.530376000	17.324087000	-16.909644000
6	1.912480000	17.577896000	-16.725256000
6	0.154506000	16.381640000	-17.899253000
6	1.122800000	15.686486000	-18.638784000
6	2.505565000	15.906691000	-18.418948000
6	2.878523000	16.884997000	-17.463781000
5	3.607825000	15.022731000	-19.089067000
8	3.290780000	14.005777000	-19.980215000
5	4.258979000	13.107788000	-20.417298000
8	5.570092000	13.265184000	-19.980215000
5	5.911348000	14.274270000	-19.089067000
8	4.935260000	15.189169000	-18.716408000
1	2.226343000	18.311291000	-15.964264000
1	-0.915665000	16.172081000	-18.064987000
1	0.808406000	14.934242000	-19.381678000
1	3.948927000	17.075964000	-17.281462000
6	7.322673000	14.341518000	-18.418948000
6	8.311921000	13.350598000	-18.638784000
6	7.595978000	15.352203000	-17.463781000
6	8.784799000	15.344944000	-16.725256000
6	9.753760000	14.327228000	-16.909644000
6	9.503888000	13.343842000	-17.899253000
1	8.124113000	12.557224000	-19.381678000
1	6.842251000	16.135867000	-17.281462000
1	8.961957000	16.122757000	-15.964264000
1	10.246500000	12.545274000	-18.064987000
6	20.438244000	5.906898000	-4.999773000
6	20.747012000	5.317488000	-3.748628000
6	19.942134000	7.234654000	-5.005925000
6	19.736317000	7.933766000	-3.808734000
6	20.028412000	7.337614000	-2.556757000
6	20.551258000	6.020191000	-2.552796000
1	21.135590000	4.285881000	-3.721935000
1	19.699758000	7.713153000	-5.969445000
1	19.330537000	8.958920000	-3.835007000
1	20.789706000	5.540319000	-1.589100000
6	11.388441000	17.189204000	-10.096939000
6	10.615187000	17.465446000	-11.252127000
6	12.440783000	16.246796000	-10.211552000
6	12.679101000	15.574745000	-11.419533000
6	11.874587000	15.818238000	-12.560801000
6	10.852842000	16.794822000	-12.457325000
5	11.994104000	14.960478000	-13.862680000

8	11.076694000	15.144767000	-14.888734000
5	10.979346000	14.244681000	-15.941924000
8	11.929620000	13.236809000	-16.044451000
5	12.903503000	13.068308000	-15.065729000
8	12.935828000	13.947829000	-13.988576000
1	9.795261000	18.199383000	-11.185586000
1	13.060279000	16.020428000	-9.327466000
1	13.484169000	14.823102000	-11.478420000
1	10.218724000	17.002950000	-13.334844000
6	18.713488000	6.405299000	-12.560801000
6	18.730520000	7.245680000	-11.419533000
6	19.326538000	5.131781000	-12.457325000
6	19.890899000	4.698523000	-11.252127000
6	19.867126000	5.519294000	-10.096939000
6	19.296034000	6.811352000	-10.211552000
5	20.349801000	4.936690000	-8.728575000
8	20.290876000	5.674545000	-7.553197000
8	20.770192000	3.614888000	-8.662739000
5	20.578337000	5.089388000	-6.324598000
8	20.940615000	3.746574000	-6.285613000
5	20.986913000	2.983670000	-7.444993000
1	18.264445000	8.243616000	-11.478420000
1	19.328526000	4.464383000	-13.334844000
1	20.335544000	3.691928000	-11.185586000
1	19.272180000	7.470480000	-9.327466000
6	-10.611926000	13.703734000	-16.909644000
6	-9.753885000	13.162209000	-17.899253000
6	-11.879256000	13.096689000	-16.725256000
6	-12.253527000	11.968296000	-17.463781000
6	-11.376763000	11.396049000	-18.418948000
6	-10.128649000	12.030668000	-18.638784000
5	-11.748931000	10.033018000	-19.089067000
8	-10.894686000	9.396640000	-19.980215000
5	-11.150151000	8.101059000	-20.417298000
8	-12.303379000	7.457741000	-19.980215000
5	-13.172587000	8.073524000	-19.089067000
8	-12.920679000	9.387423000	-18.716408000
1	-8.764922000	13.621703000	-18.064987000
1	-12.564256000	13.505534000	-15.964264000
1	-13.231749000	11.493624000	-17.281462000
1	-9.432141000	11.606887000	-19.381678000
6	-14.353900000	7.298372000	-18.418948000
6	-14.571770000	5.915237000	-18.638784000
6	-15.169074000	7.955390000	-17.463781000
6	-16.126583000	7.250745000	-16.725256000
6	-16.312291000	5.857855000	-16.909644000
6	-15.532120000	5.209149000	-17.899253000
1	-13.953497000	5.383774000	-19.381678000
1	-15.019921000	9.032416000	-17.281462000
1	-16.727095000	7.775878000	-15.964264000
1	-15.663519000	4.126598000	-18.064987000
6	5.181359000	20.577336000	-7.409885000

6	5.894363000	20.411020000	-6.196229000
6	5.872412000	20.337823000	-8.623862000
6	7.203391000	19.905362000	-8.623862000
6	7.903247000	19.692941000	-7.409885000
6	7.228657000	19.977482000	-6.196229000
5	9.322952000	19.037735000	-7.444993000
8	9.856305000	18.636565000	-8.662739000
5	10.983505000	17.828289000	-8.728575000
8	11.667039000	17.544239000	-7.553197000
5	11.199352000	17.998454000	-6.324598000
8	10.034210000	18.757954000	-6.285613000
1	5.337965000	20.466266000	-9.579508000
1	7.711265000	19.695134000	-9.579508000
1	7.754640000	19.825021000	-5.238792000
6	11.933558000	17.612593000	-4.999773000
6	11.468411000	18.088386000	-3.748628000
6	13.043023000	16.730465000	-5.005925000
6	13.644318000	16.318685000	-3.808734000
6	13.167605000	16.780704000	-2.556757000
6	12.076230000	17.685066000	-2.552796000
1	10.607372000	18.776731000	-3.721935000
1	13.423204000	16.352088000	-5.969445000
1	14.493904000	15.615975000	-3.835007000
1	11.693529000	18.060132000	-1.589100000
6	-12.619112000	15.511552000	-12.457325000
6	-12.828683000	16.142807000	-10.096939000
6	-11.607208000	16.852424000	-10.211552000
6	-11.374590000	16.181508000	-12.560801000
6	-10.894405000	16.871402000	-11.419533000
5	-13.561621000	15.955179000	-8.728575000
8	-14.678661000	15.132919000	-8.662739000
8	-13.080250000	16.517481000	-7.553197000
5	-10.521878000	16.030113000	-13.862680000
8	-10.980642000	15.214552000	-14.888734000
5	-10.154692000	14.843827000	-15.941924000
8	-8.902498000	15.436142000	-16.044451000
5	-8.441297000	16.310290000	-15.065729000
8	-9.267783000	16.612820000	-13.988576000
6	-13.062899000	16.792079000	-4.999773000
6	-13.659144000	16.496725000	-3.748628000
6	-11.881102000	17.574650000	-5.005925000
6	-11.303665000	18.019268000	-3.808734000
6	-11.890384000	17.708660000	-2.556757000
6	-13.087737000	16.950163000	-2.552796000
5	-15.225012000	14.749638000	-7.444993000
8	-14.739133000	15.339627000	-6.285613000
5	-13.656757000	16.213044000	-6.324598000
6	-16.286862000	13.601889000	-7.409885000
6	-16.705154000	13.001927000	-8.623862000
6	-17.527744000	11.869729000	-8.623862000
6	-17.969082000	11.286512000	-7.409885000
6	-16.765936000	13.048242000	-6.196229000

6	-17.590575000	11.913225000	-6.196229000
1	-13.013007000	14.972785000	-13.334844000
1	-11.200484000	17.371648000	-9.327466000
1	-9.930770000	17.404797000	-11.478420000
1	-14.579874000	15.890539000	-3.721935000
1	-11.403762000	17.819299000	-5.969445000
1	-10.372812000	18.610123000	-3.835007000
1	-13.562707000	16.702095000	-1.589100000
1	-16.348274000	13.419980000	-9.579508000
1	-17.815054000	11.401131000	-9.579508000
1	-16.458399000	13.501369000	-5.238792000
1	-17.926490000	11.480715000	-5.238792000
6	-13.330353000	15.492762000	-11.252127000
1	-14.281740000	14.939765000	-11.185586000
6	-0.309015000	19.776931000	-12.560801000
6	1.091615000	19.966437000	-12.457325000
6	-1.103002000	20.052822000	-11.419533000
6	-0.515178000	20.456443000	-10.211552000
6	1.678065000	20.369293000	-11.252127000
6	0.890119000	20.600316000	-10.096939000
6	-0.789366000	21.315599000	-2.556757000
6	-1.446603000	21.222022000	-3.808734000
6	0.625146000	21.405749000	-2.552796000
6	1.353948000	21.374775000	-3.748628000
6	0.697971000	21.263257000	-4.999773000
6	-0.718106000	21.201727000	-5.005925000
5	1.518760000	21.143869000	-6.324598000
8	0.873412000	21.051301000	-7.553197000
5	1.593363000	20.879332000	-8.728575000
8	2.980380000	20.870689000	-8.662739000
5	3.647674000	20.881745000	-7.444993000
8	2.907802000	21.073464000	-6.285613000
1	1.726962000	19.762091000	-13.334844000
1	-2.196121000	19.917937000	-11.478420000
1	-1.149417000	20.637438000	-9.327466000
1	2.772797000	20.481120000	-11.185586000
1	-2.546975000	21.152892000	-3.835007000
1	1.155216000	21.484238000	-1.589100000
1	2.455141000	21.425551000	-3.721935000
1	-1.248084000	21.119079000	-5.969445000
6	-20.516267000	5.836150000	-2.556757000
6	-20.630367000	5.182165000	-3.808734000
6	-20.164897000	7.209289000	-2.552796000
6	-19.910226000	7.892849000	-3.748628000
6	-20.006874000	7.234518000	-4.999773000
6	-20.385948000	5.868734000	-5.005925000
5	-19.639692000	7.978241000	-6.324598000
8	-19.143495000	9.277543000	-6.285613000
8	-19.751078000	7.335874000	-7.553197000
5	-19.365048000	7.967447000	-8.728575000
8	-18.928217000	9.283907000	-8.662739000
5	-18.732526000	9.921958000	-7.444993000

6	-6.990153000	16.886483000	-15.132168000
6	-6.107255000	16.528858000	-16.181517000
6	-6.494278000	17.720752000	-14.099421000
6	-4.774546000	16.961881000	-16.181517000
6	-4.270473000	17.770161000	-15.132168000
6	-5.162015000	18.153630000	-14.099421000
5	-2.757795000	18.156972000	-15.065729000
8	-2.266977000	18.887519000	-13.988576000
8	-1.870864000	17.720859000	-16.044451000
5	-0.509665000	17.977686000	-15.941924000
8	-0.059363000	18.763091000	-14.888734000
5	-0.909886000	19.153239000	-13.862680000
1	-20.904654000	4.114286000	-3.835007000
1	-20.075743000	7.737670000	-1.589100000
1	-19.618230000	8.955837000	-3.721935000
1	-20.471117000	5.339156000	-5.969445000
1	-6.474970000	15.883919000	-16.996938000
1	-7.166807000	18.013556000	-13.276103000
1	-4.097973000	16.656252000	-16.996938000
1	-4.790033000	18.785816000	-13.276103000
6	-18.904470000	-5.817517000	-12.560801000
6	-18.651883000	-7.208156000	-12.457325000
6	-19.412213000	-5.147645000	-11.419533000
6	-19.614432000	-5.831425000	-10.211552000
6	-18.853798000	-7.890393000	-11.252127000
1	-18.261205000	-7.749261000	-13.334844000
1	-19.621722000	-4.066346000	-11.478420000
1	-19.982559000	-5.284158000	-9.327466000
1	-18.621861000	-8.966101000	-11.185586000
6	-19.317003000	7.212401000	-10.096939000
6	-18.853798000	7.890393000	-11.252127000
6	-19.614432000	5.831425000	-10.211552000
6	-19.412213000	5.147645000	-11.419533000
6	-18.904470000	5.817517000	-12.560801000
6	-18.651883000	7.208156000	-12.457325000
5	-18.496983000	5.053324000	-13.862680000
8	-17.863104000	5.741656000	-14.888734000
5	-17.255291000	5.070691000	-15.941924000
8	-17.431667000	3.696749000	-16.044451000
5	-18.120512000	2.987994000	-15.065729000
8	-18.663633000	3.680541000	-13.988576000
1	-18.621861000	8.966101000	-11.185586000
1	-19.982559000	5.284158000	-9.327466000
1	-19.621722000	4.066346000	-11.478420000
1	-18.261205000	7.749261000	-13.334844000
6	-18.220076000	1.429821000	-15.132168000
6	-18.860279000	0.700412000	-14.099421000
6	-17.607123000	0.700646000	-16.181517000
6	-17.607123000	-0.700646000	-16.181517000
6	-18.220076000	-1.429821000	-15.132168000
6	-18.860279000	-0.700412000	-14.099421000
5	-18.120512000	-2.987994000	-15.065729000

8	-18.663633000	-3.680541000	-13.988576000
8	-17.431667000	-3.696749000	-16.044451000
5	-18.496983000	-5.053324000	-13.862680000
8	-17.863104000	-5.741656000	-14.888734000
5	-17.255291000	-5.070691000	-15.941924000
1	-19.346575000	1.249544000	-13.276103000
1	-17.107381000	1.249661000	-16.996938000
1	-17.107381000	-1.249661000	-16.996938000
1	-19.346575000	-1.249544000	-13.276103000
6	-19.317003000	-7.212401000	-10.096939000
5	-19.365048000	-7.967447000	-8.728575000
8	-18.928217000	-9.283907000	-8.662739000
5	-18.732526000	-9.921958000	-7.444993000
8	-19.143495000	-9.277543000	-6.285613000
5	-19.639692000	-7.978241000	-6.324598000
8	-19.751078000	-7.335874000	-7.553197000
6	-20.006874000	-7.234518000	-4.999773000
6	-20.385948000	-5.868734000	-5.005925000
6	-19.910226000	-7.892849000	-3.748628000
6	-20.164897000	-7.209289000	-2.552796000
6	-20.516267000	-5.836150000	-2.556757000
6	-20.630367000	-5.182165000	-3.808734000
1	-20.471117000	-5.339156000	-5.969445000
1	-19.618230000	-8.955837000	-3.721935000
1	-20.075743000	-7.737670000	-1.589100000
1	-20.904654000	-4.114286000	-3.835007000
5	17.934643000	6.784029000	-13.862680000
8	17.826416000	5.854572000	-14.888734000
8	17.262564000	7.992588000	-13.988576000
5	16.940301000	6.040131000	-15.941924000
8	16.275409000	7.255344000	-16.044451000
5	16.416101000	8.233632000	-15.065729000
6	15.580779000	9.552743000	-15.132168000
6	15.669978000	10.519149000	-14.099421000
6	14.656292000	9.782372000	-16.181517000
6	14.846594000	11.652439000	-14.099421000
6	13.832632000	10.916042000	-16.181517000
6	13.899924000	11.866241000	-15.132168000
1	16.386171000	10.360729000	-13.276103000
1	14.574694000	9.044469000	-16.996938000
1	14.917244000	12.382534000	-13.276103000
1	13.105629000	11.066463000	-16.996938000
5	2.677694000	8.241096000	-23.506620000
8	1.338956000	7.999340000	-23.797175000
8	3.618656000	7.258621000	-23.797175000
5	3.235902000	6.039452000	-24.341180000
8	1.901640000	5.852646000	-24.677802000
5	0.932001000	6.788035000	-24.341180000
6	-0.578705000	6.411564000	-24.488691000
6	-0.933274000	5.136767000	-24.996442000
6	-1.619402000	7.253945000	-24.023621000
6	-2.953638000	6.820425000	-24.023621000

6	-3.300440000	5.527219000	-24.488691000
6	-2.264281000	4.704297000	-24.996442000
1	-0.139629000	4.467824000	-25.367921000
1	-1.368445000	8.253018000	-23.629293000
1	-3.743908000	7.481184000	-23.629293000
1	-2.513159000	3.696617000	-25.367921000
6	20.028412000	-7.337614000	-2.556757000
6	20.551258000	-6.020191000	-2.552796000
6	19.736317000	-7.933766000	-3.808734000
6	19.942134000	-7.234654000	-5.005925000
6	20.438244000	-5.906898000	-4.999773000
6	20.747012000	-5.317488000	-3.748628000
5	20.578337000	-5.089388000	-6.324598000
8	20.290876000	-5.674545000	-7.553197000
8	20.940615000	-3.746574000	-6.285613000
5	20.349801000	-4.936690000	-8.728575000
8	20.770192000	-3.614888000	-8.662739000
5	20.986913000	-2.983670000	-7.444993000
6	21.171338000	-1.430982000	-7.409885000
6	21.157094000	-0.699737000	-8.623862000
6	21.233492000	-0.701480000	-6.196229000
6	21.233492000	0.701480000	-6.196229000
6	21.171338000	1.430982000	-7.409885000
6	21.157094000	0.699737000	-8.623862000
1	20.789706000	-5.540319000	-1.589100000
1	19.330537000	-8.958920000	-3.835007000
1	19.699758000	-7.713153000	-5.969445000
1	21.135590000	-4.285881000	-3.721935000
1	21.114098000	-1.247718000	-9.579508000
1	21.251031000	-1.248833000	-5.238792000
1	21.251031000	1.248833000	-5.238792000
1	21.114098000	1.247718000	-9.579508000
5	-11.150151000	-8.101059000	-20.417298000
8	-10.894686000	-9.396640000	-19.980215000
8	-12.303379000	-7.457741000	-19.980215000
5	-13.172587000	-8.073524000	-19.089067000
8	-12.920679000	-9.387423000	-18.716408000
5	-11.748931000	-10.033018000	-19.089067000
6	-14.571770000	-5.915237000	-18.638784000
6	-15.169074000	-7.955390000	-17.463781000
6	-16.126583000	-7.250745000	-16.725256000
6	-15.532120000	-5.209149000	-17.899253000
6	-16.312291000	-5.857855000	-16.909644000
6	-14.353900000	-7.298372000	-18.418948000
1	-13.953497000	-5.383774000	-19.381678000
1	-15.019921000	-9.032416000	-17.281462000
1	-16.727095000	-7.775878000	-15.964264000
1	-15.663519000	-4.126598000	-18.064987000
1	5.379219000	20.596842000	-5.238792000
6	-17.969082000	-11.286512000	-7.409885000
6	-17.590575000	-11.913225000	-6.196229000
6	-17.527744000	-11.869729000	-8.623862000

6	-16.705154000	-13.001927000	-8.623862000
6	-16.286862000	-13.601889000	-7.409885000
6	-16.765936000	-13.048242000	-6.196229000
5	-15.225012000	-14.749638000	-7.444993000
8	-14.678661000	-15.132919000	-8.662739000
5	-13.561621000	-15.955179000	-8.728575000
8	-13.080250000	-16.517481000	-7.553197000
5	-13.656757000	-16.213044000	-6.324598000
8	-14.739133000	-15.339627000	-6.285613000
1	-17.815054000	-11.401131000	-9.579508000
1	-16.348274000	-13.419980000	-9.579508000
1	-16.458399000	-13.501369000	-5.238792000
6	-13.062899000	-16.792079000	-4.999773000
6	-13.659144000	-16.496725000	-3.748628000
6	-11.881102000	-17.574650000	-5.005925000
6	-11.303665000	-18.019268000	-3.808734000
6	-11.890384000	-17.708660000	-2.556757000
6	-13.087737000	-16.950163000	-2.552796000
1	-14.579874000	-15.890539000	-3.721935000
1	-11.403762000	-17.819299000	-5.969445000
1	-10.372812000	-18.610123000	-3.835007000
1	-13.562707000	-16.702095000	-1.589100000
1	-17.926490000	-11.480715000	-5.238792000
6	-11.376763000	-11.396049000	-18.418948000
6	-12.253527000	-11.968296000	-17.463781000
6	-11.879256000	-13.096689000	-16.725256000
6	-10.611926000	-13.703734000	-16.909644000
6	-9.753885000	-13.162209000	-17.899253000
6	-10.128649000	-12.030668000	-18.638784000
1	-13.231749000	-11.493624000	-17.281462000
1	-12.564256000	-13.505534000	-15.964264000
1	-8.764922000	-13.621703000	-18.064987000
1	-9.432141000	-11.606887000	-19.381678000
6	-3.300440000	-5.527219000	-24.488691000
6	-2.953638000	-6.820425000	-24.023621000
6	-2.264281000	-4.704297000	-24.996442000
6	-1.619402000	-7.253945000	-24.023621000
6	-0.578705000	-6.411564000	-24.488691000
6	-0.933274000	-5.136767000	-24.996442000
1	-3.743908000	-7.481184000	-23.629293000
1	-2.513159000	-3.696617000	-25.367921000
1	-1.368445000	-8.253018000	-23.629293000
1	-0.139629000	-4.467824000	-25.367921000

Cartesian coordinates for COBNC-20-BDBA

6	8.997837667	7.419880181	-1.376843810
6	8.607060832	8.693236728	0.692812327
6	9.824954487	8.156691459	1.181539712
6	10.180579357	6.831316735	-0.866258632
6	10.589231847	7.253822259	0.424116843

5	7.583395644	9.518364783	1.572702899
7	7.442171873	9.289594616	2.993276223
7	6.619286704	10.402595704	0.961260935
5	10.825459836	5.616962139	-1.649292897
7	11.469607769	4.525157606	-0.961260935
5	11.729850398	3.243110746	-1.572702899
7	11.481130233	3.141041043	-2.993276223
5	10.851134066	4.192525836	-3.757938168
7	10.630187896	5.440552399	-3.067439473
6	4.220914481	11.510645735	0.866258632
6	2.918107442	11.291605444	1.376843810
6	4.303178773	12.092659797	-0.424116843
6	3.154172206	12.373865360	-1.181539712
6	1.853502143	12.092079676	-0.692812327
6	1.771669496	11.573192570	0.622452275
5	6.314447011	9.769961223	3.757938168
7	5.402126198	10.649767025	3.067439473
5	5.456413471	10.907263468	1.649292897
6	12.073014833	1.973872827	-0.692812327
6	12.742938096	0.823938655	-1.181539712
6	11.554236192	1.891355367	0.622452275
6	11.640699727	0.714012877	1.376843810
6	12.251608942	-0.457343073	0.866258632
6	12.830558270	-0.355728827	-0.424116843
1	8.620643775	7.118934735	-2.369999607
1	10.179880489	8.438336783	2.189581572
1	11.522273976	6.853643167	0.861367632
1	2.789842470	10.826426457	2.369999607
1	5.293245079	12.317336514	-0.861367632
1	3.275766401	12.810341481	-2.189581572
1	0.786258690	11.318713824	1.051215950
1	13.195626229	0.843174243	-2.189581572
1	11.007703839	2.749898473	1.051215950
1	11.158652168	0.692251907	2.369999607
1	13.350185843	-1.227908921	-0.861367632
6	8.235862646	8.321548269	-0.622452275
1	7.289069703	8.694880575	-1.051215950
5	11.395900983	-4.270901368	1.572702899
7	11.938928509	-3.080736893	0.961260935
7	11.134687075	-4.207283446	2.993276223
5	11.243056723	-2.986311923	3.757938168
5	12.059548489	-1.818827815	1.649292897
7	11.797879129	-1.846768326	3.067439473
6	10.927487506	-5.499443408	0.692812327
6	10.459284229	-5.261270999	-0.622452275
6	10.793552464	-6.823530712	1.181539712
6	10.171047525	-7.829403596	0.424116843
6	9.642940326	-7.571313374	-0.866258632
6	9.837210147	-6.264583073	-1.376843810
1	10.521769241	-4.245451377	-1.051215950
1	11.171091257	-7.074052202	2.189581572
1	10.078780471	-8.840441535	0.861367632

1	9.434434700	-5.998847626	-2.369999607
6	10.256435286	3.956375303	-5.205434729
6	10.754833570	3.014499342	-6.140247052
6	9.063133102	4.630533735	-5.564221662
6	8.389001869	4.354969313	-6.760405978
6	10.078880285	2.738496444	-7.341236404
6	8.864119974	3.386973397	-7.678771165
1	11.695084956	2.475644050	-5.923648221
1	8.614063763	5.357512312	-4.864802027
1	7.434659826	4.875002464	-6.956605169
1	10.508884770	1.991562826	-8.033040002
6	4.276240645	-10.850321218	1.376843810
6	5.608031373	-10.872159178	-0.692812327
6	4.721396654	-11.864643263	-1.181539712
6	3.350996260	-11.793299301	0.866258632
6	3.626542330	-12.312512301	-0.424116843
5	6.709104726	-10.153574320	-1.572702899
7	7.847984348	-9.509894608	-0.961260935
7	6.535171906	-9.948568662	-2.993276223
5	1.996797384	-12.031360878	1.649292897
7	1.889364099	-11.791132622	3.067439473
5	0.634144181	-11.615603491	3.757938168
7	-0.560556804	-11.889838784	2.993276223
5	-0.540341508	-12.157926995	1.572702899
7	0.759376909	-12.306595814	0.961260935
6	-1.853502143	-12.092079676	0.692812327
6	-1.771669496	-11.573192570	-0.622452275
6	-3.154172206	-12.373865360	1.181539712
6	-4.303178773	-12.092659797	0.424116843
6	-2.918107442	-11.291605444	-1.376843810
6	-4.220914481	-11.510645735	-0.866258632
6	6.932149316	-8.531862406	-5.205434729
6	6.190385585	-9.296923023	-6.140247052
6	7.204561435	-7.188638179	-5.564221662
6	6.734166089	-6.632655362	-6.760405978
6	5.960366830	-7.383646721	-7.678771165
6	5.719010182	-8.739342831	-7.341236404
5	5.318618599	-6.644381818	-8.922080308
7	4.016038111	-6.996420227	-9.440265588
7	5.941695065	-5.470059354	-9.486976534
5	5.272389093	-4.572773985	-10.396203581
5	3.282747535	-6.158929242	-10.361850130
7	3.989822448	-5.015220501	-10.887336643
6	1.733636538	-6.351297152	-10.627788844
6	1.041179733	-7.579802218	-10.479814481
6	0.935584154	-5.205130437	-10.864255079
6	-0.463767015	-5.268240136	-10.881197425
6	-1.159814684	-6.482004383	-10.662242077
6	-0.362256594	-7.643029364	-10.497080995
5	-2.719708667	-6.427404874	-10.396203581
7	-3.366250842	-7.341229106	-9.486976534
7	-3.536835197	-5.344334999	-10.887336643

5	-4.843065008	-5.025292237	-10.361850130
7	-5.412967018	-5.981491967	-9.440265588
5	-4.675639089	-7.111533773	-8.922080308
6	-5.180410689	-7.950318031	-7.678771165
6	-6.544337610	-8.139707356	-7.341236404
6	-4.227058340	-8.454175762	-6.760405978
6	-4.610469260	-9.073356462	-5.564221662
6	-5.972131393	-9.229356259	-5.205434729
6	-6.928964876	-8.760313760	-6.140247052
5	-6.314447011	-9.769961223	-3.757938168
7	-5.402126198	-10.649767025	-3.067439473
5	-5.456413471	-10.907263468	-1.649292897
7	-6.619286704	-10.402595704	-0.961260935
5	-7.583395644	-9.518364783	-1.572702899
7	-7.442171873	-9.289594616	-2.993276223
5	8.687299508	-8.559887363	-1.649292897
7	8.459181525	-8.428686321	-3.067439473
5	7.340513849	-9.024480027	-3.757938168
6	5.806350061	-3.106098824	-10.662242077
6	4.867082220	-2.069044371	-10.881197425
6	5.239484624	-0.718680353	-10.864255079
6	6.576165696	-0.313872431	-10.627788844
6	7.530562524	-1.352066927	-10.479814481
6	7.157009435	-2.706352456	-10.497080995
1	4.106583836	-10.398591255	2.369999607
1	4.879579111	-12.289231143	-2.189581572
1	2.957623525	-13.076225960	-0.861367632
1	-0.786258690	-11.318713824	-1.051215950
1	-3.275766401	-12.810341481	2.189581572
1	-5.293245079	-12.317336514	0.861367632
1	-2.789842470	-10.826426457	-2.369999607
1	5.968457405	-10.357670677	-5.923648221
1	7.757189089	-6.536899124	-4.864802027
1	6.933839096	-5.564323068	-6.956605169
1	5.141512790	-9.379116579	-8.033040002
1	1.612067590	-8.513760983	-10.327102812
1	1.415186940	-4.216667770	-10.974168655
1	-1.028188040	-4.327078915	-11.003189169
1	-0.848485299	-8.625798718	-10.357043618
1	-7.331255110	-7.788175656	-8.033040002
1	-3.149311596	-8.313943246	-6.956605169
1	-3.819857251	-9.397538150	-4.864802027
1	-8.006375422	-8.877036571	-5.923648221
1	3.797569020	-2.315005856	-11.003189169
1	4.447606172	0.042900765	-10.974168655
1	8.595224143	-1.097729445	-10.327102812
1	7.941425699	-3.472475867	-10.357043618
6	5.369241187	-10.404270671	0.622452275
1	6.016876420	-9.619183104	1.051215950
6	-10.053988142	1.580995492	-5.564221662
6	-9.162036710	2.470079958	-7.678771165
6	-9.763633258	3.708727028	-7.341236404

6	-10.623129502	2.827806546	-5.205434729
6	-10.472721384	3.882751369	-6.140247052
5	-8.208322478	2.249212233	-8.922080308
7	-8.022152498	0.932930245	-9.486976534
7	-7.361435713	3.299654885	-9.440265588
5	-11.243056723	2.986311923	-3.757938168
7	-11.797879129	1.846768326	-3.067439473
5	-12.059548489	1.818827815	-1.649292897
7	-11.938928509	3.080736893	-0.961260935
5	-11.395900983	4.270901368	-1.572702899
7	-11.134687075	4.207283446	-2.993276223
6	-5.135740218	4.119301342	-10.627788844
6	-5.297628059	5.520198718	-10.479814481
6	-3.816402385	3.661116411	-10.864255079
6	-2.721398463	4.534691210	-10.881197425
6	-2.871716795	5.925773672	-10.662242077
6	-4.199388201	6.396269731	-10.497080995
5	-1.577643266	6.798484414	-10.396203581
7	-1.591712062	7.917811709	-9.486976534
7	-0.279961515	6.402557409	-10.887336643
5	0.964329231	6.912229010	-10.361850130
5	-0.397383189	8.501623382	-8.922080308
7	0.863349544	8.020790835	-9.440265588
5	-6.953261483	0.600419313	-10.396203581
7	-6.175706812	1.712239822	-10.887336643
5	-6.275926320	3.053127832	-10.361850130
6	2.330657379	6.157313320	-10.627788844
6	3.612963859	6.744178901	-10.479814481
6	4.785531683	5.970411561	-10.497080995
6	4.748336370	4.562329736	-10.662242077
6	3.471789254	3.989500391	-10.881197425
6	2.302595422	4.760961549	-10.864255079
6	-6.523154957	-0.900000201	-10.662242077
6	-7.380896323	-2.017299467	-10.497080995
6	-6.887078062	-3.332508468	-10.479814481
6	-5.504719389	-3.611445083	-10.627788844
6	-5.153706002	-1.186907093	-10.881197425
6	-4.661261814	-2.498267171	-10.864255079
6	-12.251608942	0.457343073	-0.866258632
6	-12.830558270	0.355728827	0.424116843
6	-11.640699727	-0.714012877	-1.376843810
6	-11.554236192	-1.891355367	-0.622452275
6	-12.073014833	-1.973872827	0.692812327
6	-12.742938096	-0.823938655	1.181539712
1	-10.117990701	0.728901136	-4.864802027
1	-9.672477629	4.565759312	-8.033040002
1	-10.916669546	4.871360354	-5.923648221
1	-6.308453227	5.940227767	-10.327102812
1	-3.623405415	2.579529868	-10.974168655
1	-1.711571577	4.105034141	-11.003189169
1	-4.383678253	7.477144896	-10.357043618
1	3.700073070	7.835326878	-10.327102812

1	5.756556297	6.479690608	-10.357043618
1	1.333584840	4.243181900	-10.974168655
1	-8.465818456	-1.858560924	-10.357043618
1	-7.598911581	-4.164064218	-10.327102812
1	-4.433024174	-0.359275987	-11.003189169
1	-3.572972542	-2.648944762	-10.974168655
1	-13.350185843	1.227908921	0.861367632
1	-11.158652168	-0.692251907	-2.369999607
1	-11.007703839	-2.749898473	-1.051215950
1	-13.195626229	-0.843174243	2.189581572
6	-9.346631812	1.407687393	-6.760405978
1	-8.880220705	0.426023564	-6.956605169
5	-10.851134066	-4.192525836	3.757938168
7	-11.481130233	-3.141041043	2.993276223
7	-10.630187896	-5.440552399	3.067439473
5	-10.825459836	-5.616962139	1.649292897
5	-11.729850398	-3.243110746	1.572702899
7	-11.469607769	-4.525157606	0.961260935
6	-10.180579357	-6.831316735	0.866258632
6	-10.589231847	-7.253822259	-0.424116843
6	-9.824954487	-8.156691459	-1.181539712
6	-8.607060832	-8.693236728	-0.692812327
6	-8.235862646	-8.321548269	0.622452275
6	-8.997837667	-7.419880181	1.376843810
1	-11.522273976	-6.853643167	-0.861367632
1	-10.179880489	-8.438336783	-2.189581572
1	-7.289069703	-8.694880575	1.051215950
1	-8.620643775	-7.118934735	2.369999607
5	6.871914562	1.218864631	-10.361850130
7	6.002681081	2.244758270	-10.887336643
5	5.978224324	3.601275127	-10.396203581
7	7.895015071	1.657466468	-9.440265588
7	7.038420343	3.960546505	-9.486976534
5	7.962726158	3.005079977	-8.922080308
6	-10.927487506	5.499443408	-0.692812327
6	-10.793552464	6.823530712	-1.181539712
6	-10.459284229	5.261270999	0.622452275
6	-9.837210147	6.264583073	1.376843810
6	-10.171047525	7.829403596	-0.424116843
6	-9.642940326	7.571313374	0.866258632
6	-0.482039404	9.476911403	-7.678771165
6	0.510080401	10.431826709	-7.341236404
6	-1.549477801	9.324174418	-6.760405978
6	-1.603237130	10.050465414	-5.564221662
6	-0.593323708	10.977036817	-5.205434729
6	0.456467104	11.159986076	-6.140247052
5	-0.634144181	11.615603491	-3.757938168
7	-1.889364099	11.791132622	-3.067439473
7	0.560556804	11.889838784	-2.993276223
5	0.540341508	12.157926995	-1.572702899
7	-0.759376909	12.306595814	-0.961260935
5	-1.996797384	12.031360878	-1.649292897

6	-3.350996260	11.793299301	-0.866258632
6	-4.276240645	10.850321218	-1.376843810
6	-3.626542330	12.312512301	0.424116843
6	-4.721396654	11.864643263	1.181539712
6	-5.608031373	10.872159178	0.692812327
6	-5.369241187	10.404270671	-0.622452275
5	-6.709104726	10.153574320	1.572702899
7	-7.847984348	9.509894608	0.961260935
5	-8.687299508	8.559887363	1.649292897
7	-8.459181525	8.428686321	3.067439473
5	-7.340513849	9.024480027	3.757938168
7	-6.535171906	9.948568662	2.993276223
1	-11.171091257	7.074052202	-2.189581572
1	-10.521769241	4.245451377	1.051215950
1	-9.434434700	5.998847626	2.369999607
1	-10.078780471	8.840441535	-0.861367632
1	1.353335180	10.609970097	-8.033040002
1	-2.338966626	8.577240287	-6.956605169
1	-2.433404900	9.848023826	-4.864802027
1	1.259502597	11.887702839	-5.923648221
1	-4.106583836	10.398591255	-2.369999607
1	-2.957623525	13.076225960	0.861367632
1	-4.879579111	12.289231143	2.189581572
1	-6.016876420	9.619183104	-1.051215950
1	3.375214766	2.896326612	-11.003189169
1	12.086234363	-3.091129585	-0.052745123
1	11.909427698	-0.976509283	3.596093085
1	10.641030044	-4.996960383	3.421639396
1	-12.086234363	3.091129585	0.052745123
1	-10.641030044	4.996960383	-3.421639396
1	-11.909427698	0.976509283	-3.596093085
1	7.961048618	-9.604886682	0.052745123
1	-5.430601924	1.518141385	-11.562888510
1	9.060951648	-7.790198568	-3.596093085
1	5.671634525	-10.297266401	-3.421639396
1	8.040650857	8.576075287	3.421639396
1	6.674690752	10.539480375	-0.052745123
1	11.594889382	4.603333950	0.052745123
1	11.545913763	2.212014660	-3.421639396
1	10.208907157	6.210173359	-3.596093085
1	4.608931069	11.024780855	3.596093085
1	2.751500033	-11.628296781	3.596093085
1	-1.464132613	-11.664366645	3.421639396
1	0.795012885	-12.449903522	-0.052745123
1	3.505812102	-7.750380472	-8.969195207
1	6.842910088	-5.175920900	-9.098000389
1	3.501108129	-4.420229901	-11.562888510
1	-2.808017793	-8.107441752	-9.098000389
1	-3.121986540	-4.695677857	-11.562888510
1	-6.287694333	-5.729224721	-8.969195207
1	-4.608931069	-11.024780855	-3.596093085
1	-6.674690752	-10.539480375	0.052745123

1	-8.040650857	-8.576075287	-3.421639396
1	-8.578360523	0.165246338	-9.098000389
1	-7.391820912	4.209524867	-8.969195207
1	-2.493700578	8.209569601	-9.098000389
1	-0.234310028	5.633940833	-11.562888510
1	1.719297771	8.330854169	-8.969195207
1	-11.545913763	-2.212014660	3.421639396
1	-10.208907157	-6.210173359	3.596093085
1	-11.594889382	-4.603333950	-0.052745123
1	5.285790363	1.963825540	-11.562888510
1	8.454405372	0.939226163	-8.969195207
1	7.037168807	4.908546708	-9.098000389
1	-2.751500033	11.628296781	-3.596093085
1	1.464132613	11.664366645	-3.421639396
1	-0.795012885	12.449903522	0.052745123
1	-7.961048618	9.604886682	-0.052745123
1	-9.060951648	7.790198568	3.596093085
1	-5.671634525	10.297266401	3.421639396
6	10.623129502	-2.827806546	5.205434729
6	10.053988142	-1.580995492	5.564221662
6	10.472721384	-3.882751369	6.140247052
6	9.763633258	-3.708727028	7.341236404
6	9.346631812	-1.407687393	6.760405978
6	9.162036710	-2.470079958	7.678771165
1	10.117990701	-0.728901136	4.864802027
1	10.916669546	-4.871360354	5.923648221
1	9.672477629	-4.565759312	8.033040002
1	8.880220705	-0.426023564	6.956605169
6	5.972131393	9.229356259	5.205434729
6	4.610469260	9.073356462	5.564221662
6	6.928964876	8.760313760	6.140247052
6	6.544337610	8.139707356	7.341236404
6	5.180410689	7.950318031	7.678771165
6	4.227058340	8.454175762	6.760405978
5	4.675639089	7.111533773	8.922080308
7	3.366250842	7.341229106	9.486976534
7	5.412967018	5.981491967	9.440265588
5	4.843065008	5.025292237	10.361850130
5	2.719708667	6.427404874	10.396203581
7	3.536835197	5.344334999	10.887336643
6	1.159814684	6.482004383	10.662242077
6	0.362256594	7.643029364	10.497080995
6	0.463767015	5.268240136	10.881197425
6	-0.935584154	5.205130437	10.864255079
6	-1.733636538	6.351297152	10.627788844
6	-1.041179733	7.579802218	10.479814481
5	-3.282747535	6.158929242	10.361850130
7	-4.016038111	6.996420227	9.440265588
7	-3.989822448	5.015220501	10.887336643
5	-5.272389093	4.572773985	10.396203581
7	-5.941695065	5.470059354	9.486976534
5	-5.318618599	6.644381818	8.922080308

6	-5.960366830	7.383646721	7.678771165
6	-6.734166089	6.632655362	6.760405978
6	-5.719010182	8.739342831	7.341236404
6	-6.190385585	9.296923023	6.140247052
6	-6.932149316	8.531862406	5.205434729
6	-7.204561435	7.188638179	5.564221662
6	5.504719389	3.611445083	10.627788844
6	4.661261814	2.498267171	10.864255079
6	5.153706002	1.186907093	10.881197425
6	6.523154957	0.900000201	10.662242077
6	7.380896323	2.017299467	10.497080995
6	6.887078062	3.332508468	10.479814481
1	3.819857251	9.397538150	4.864802027
1	8.006375422	8.877036571	5.923648221
1	7.331255110	7.788175656	8.033040002
1	3.149311596	8.313943246	6.956605169
1	0.848485299	8.625798718	10.357043618
1	1.028188040	4.327078915	11.003189169
1	-1.415186940	4.216667770	10.974168655
1	-1.612067590	8.513760983	10.327102812
1	-6.933839096	5.564323068	6.956605169
1	-5.141512790	9.379116579	8.033040002
1	-5.968457405	10.357670677	5.923648221
1	-7.757189089	6.536899124	4.864802027
1	3.572972542	2.648944762	10.974168655
1	4.433024174	0.359275987	11.003189169
1	8.465818456	1.858560924	10.357043618
1	7.598911581	4.164064218	10.327102812
6	-10.754833570	-3.014499342	6.140247052
6	-8.864119974	-3.386973397	7.678771165
6	-8.389001869	-4.354969313	6.760405978
6	-10.256435286	-3.956375303	5.205434729
6	-9.063133102	-4.630533735	5.564221662
5	-7.962726158	-3.005079977	8.922080308
7	-7.895015071	-1.657466468	9.440265588
7	-7.038420343	-3.960546505	9.486976534
6	-4.748336370	-4.562329736	10.662242077
6	-4.785531683	-5.970411561	10.497080995
6	-3.471789254	-3.989500391	10.881197425
6	-2.302595422	-4.760961549	10.864255079
6	-2.330657379	-6.157313320	10.627788844
6	-3.612963859	-6.744178901	10.479814481
5	-0.964329231	-6.912229010	10.361850130
7	-0.863349544	-8.020790835	9.440265588
7	0.279961515	-6.402557409	10.887336643
5	1.577643266	-6.798484414	10.396203581
5	0.397383189	-8.501623382	8.922080308
7	1.591712062	-7.917811709	9.486976534
5	-6.871914562	-1.218864631	10.361850130
7	-6.002681081	-2.244758270	10.887336643
5	-5.978224324	-3.601275127	10.396203581
6	2.871716795	-5.925773672	10.662242077

6	4.199388201	-6.396269731	10.497080995
6	5.297628059	-5.520198718	10.479814481
6	5.135740218	-4.119301342	10.627788844
6	3.816402385	-3.661116411	10.864255079
6	2.721398463	-4.534691210	10.881197425
6	-6.576165696	0.313872431	10.627788844
6	-7.530562524	1.352066927	10.479814481
6	-7.157009435	2.706352456	10.497080995
6	-5.806350061	3.106098824	10.662242077
6	-5.239484624	0.718680353	10.864255079
6	-4.867082220	2.069044371	10.881197425
1	-11.695084956	-2.475644050	5.923648221
1	-7.434659826	-4.875002464	6.956605169
1	-8.614063763	-5.357512312	4.864802027
1	-5.756556297	-6.479690608	10.357043618
1	-3.375214766	-2.896326612	11.003189169
1	-1.333584840	-4.243181900	10.974168655
1	-3.700073070	-7.835326878	10.327102812
1	4.383678253	-7.477144896	10.357043618
1	6.308453227	-5.940227767	10.327102812
1	1.711571577	-4.105034141	11.003189169
1	-8.595224143	1.097729445	10.327102812
1	-7.941425699	3.472475867	10.357043618
1	-4.447606172	-0.042900765	10.974168655
1	-3.797569020	2.315005856	11.003189169
6	-10.078880285	-2.738496444	7.341236404
1	-10.508884770	-1.991562826	8.033040002
5	6.953261483	-0.600419313	10.396203581
7	6.175706812	-1.712239822	10.887336643
5	6.275926320	-3.053127832	10.361850130
7	8.022152498	-0.932930245	9.486976534
7	7.361435713	-3.299654885	9.440265588
5	8.208322478	-2.249212233	8.922080308
6	0.482039404	-9.476911403	7.678771165
6	1.549477801	-9.324174418	6.760405978
6	-0.510080401	-10.431826709	7.341236404
6	-0.456467104	-11.159986076	6.140247052
6	0.593323708	-10.977036817	5.205434729
6	1.603237130	-10.050465414	5.564221662
1	2.338966626	-8.577240287	6.956605169
1	-1.353335180	-10.609970097	8.033040002
1	-1.259502597	-11.887702839	5.923648221
1	2.433404900	-9.848023826	4.864802027
1	3.623405415	-2.579529868	10.974168655
1	-5.285790363	-1.963825540	11.562888510
1	2.808017793	8.107441752	9.098000389
1	6.287694333	5.729224721	8.969195207
1	3.121986540	4.695677857	11.562888510
1	-3.505812102	7.750380472	8.969195207
1	-3.501108129	4.420229901	11.562888510
1	-6.842910088	5.175920900	9.098000389
1	-8.454405372	-0.939226163	8.969195207

1	-7.037168807	-4.908546708	9.098000389
1	-1.719297771	-8.330854169	8.969195207
1	0.234310028	-5.633940833	11.562888510
1	2.493700578	-8.209569601	9.098000389
1	5.430601924	-1.518141385	11.562888510
1	8.578360523	-0.165246338	9.098000389
1	7.391820912	-4.209524867	8.969195207

Cartesian coordinates for COBNC-60-BDBA

6	-8.772124000	13.840989000	14.840015000
6	-7.373964000	13.889484000	14.618370000
6	-9.533987000	14.924789000	14.336729000
6	-8.938686000	15.968342000	13.612625000
6	-7.546563000	15.990051000	13.346543000
6	-6.778853000	14.932476000	13.892530000
5	-6.893133000	17.052914000	12.376319000
7	-7.643111000	17.602228000	11.271946000
5	-7.053656000	18.399528000	10.223821000
7	-5.659516000	18.733519000	10.385119000
5	-4.839148000	18.246011000	11.468216000
7	-5.505847000	17.441868000	12.464853000
1	-6.735584000	13.063367000	14.979422000
1	-10.624981000	14.951336000	14.510245000
1	-9.578357000	16.787493000	13.237169000
1	-5.690934000	14.892598000	13.704104000
6	-3.278708000	18.496775000	11.501012000
6	-2.525372000	18.551699000	12.700102000
6	-2.550998000	18.590970000	10.289199000
6	-1.153715000	18.703740000	10.275349000
6	-0.396927000	18.729719000	11.472503000
6	-1.125917000	18.664986000	12.686191000
1	-3.045376000	18.509299000	13.674321000
1	-3.086990000	18.533973000	9.325082000
1	-0.634681000	18.732172000	9.300702000
1	-0.586918000	18.708847000	13.649921000
6	3.666336000	20.964680000	-6.469255000
6	4.755814000	20.059676000	-6.475325000
6	3.125234000	21.316909000	-7.730958000
6	3.611743000	20.757901000	-8.922245000
6	4.668328000	19.813234000	-8.922927000
6	5.242181000	19.500790000	-7.666350000
1	5.206416000	19.741567000	-5.518225000
1	2.294809000	22.044038000	-7.784399000
1	3.150518000	21.060879000	-9.879655000
1	6.060178000	18.760612000	-7.608767000
6	5.532656000	21.331604000	1.188580000
6	5.880655000	20.648085000	-0.002330000
6	4.434435000	22.224047000	1.110611000
6	3.710792000	22.399977000	-0.079815000
6	4.042274000	21.693571000	-1.262820000

6	5.157867000	20.823433000	-1.190560000
5	3.184331000	21.768446000	-2.588574000
7	3.777644000	21.533628000	-3.883517000
7	1.756435000	21.982080000	-2.567594000
5	3.018402000	21.432288000	-5.105914000
7	1.600311000	21.674064000	-4.996342000
5	0.920963000	21.892581000	-3.741404000
1	6.716554000	19.926080000	0.007095000
1	4.136920000	22.803147000	2.003604000
1	2.865796000	23.112168000	-0.087418000
1	5.448006000	20.233106000	-2.077972000
6	9.835104000	19.544196000	3.668174000
6	10.255065000	18.777479000	2.553668000
6	10.729707000	19.614783000	4.765103000
6	11.947207000	18.918307000	4.766875000
6	12.342933000	18.108242000	3.672991000
6	11.472544000	18.079591000	2.556060000
1	9.589912000	18.680253000	1.676993000
1	10.464523000	20.226433000	5.646411000
1	12.606487000	19.001832000	5.649832000
1	11.725890000	17.453895000	1.681395000
6	5.900114000	15.209106000	14.825926000
6	6.423059000	16.425681000	14.320200000
6	4.526442000	14.959982000	14.586631000
6	3.735247000	15.849328000	13.842473000
6	4.271144000	17.040145000	13.293575000
6	5.632740000	17.314111000	13.576472000
5	3.424598000	17.942538000	12.309651000
7	1.990375000	18.072322000	12.405392000
5	1.182635000	18.741083000	11.412824000
7	1.892903000	19.353004000	10.314781000
5	3.323411000	19.276709000	10.144445000
7	4.054289000	18.606264000	11.192703000
1	7.481121000	16.683706000	14.507461000
1	4.070579000	14.020673000	14.947972000
1	2.682231000	15.581359000	13.642377000
1	6.090905000	18.246274000	13.199268000
6	4.052821000	19.796343000	8.841998000
6	3.410333000	19.745787000	7.580566000
6	5.403768000	20.224746000	8.850421000
6	6.084013000	20.536866000	7.663964000
6	5.455160000	20.438604000	6.397339000
6	4.091055000	20.057159000	6.393567000
5	6.257234000	20.611645000	5.046026000
7	7.649550000	20.239794000	4.961036000
7	5.633488000	21.039536000	3.816540000
5	8.384470000	20.168964000	3.721151000
7	7.681620000	20.602259000	2.538296000
5	6.298213000	21.015649000	2.535065000
1	2.361006000	19.406371000	7.516336000
1	5.941923000	20.312449000	9.811580000
1	7.138487000	20.861692000	7.725266000

1	3.555724000	19.951553000	5.432854000
6	5.287636000	8.848092000	19.473978000
6	6.334331000	8.586715000	18.556008000
6	4.885871000	10.200243000	19.611083000
6	5.476502000	11.224792000	18.854169000
6	6.504541000	10.957605000	17.916098000
6	6.924628000	9.609623000	17.800806000
5	7.118845000	12.061718000	16.966485000
7	6.339914000	13.176359000	16.480752000
5	6.825444000	14.120724000	15.502887000
7	8.197710000	13.959880000	15.083806000
5	9.038528000	12.864034000	15.501105000
7	8.471401000	11.960814000	16.472128000
1	6.672892000	7.547592000	18.395614000
1	4.091667000	10.463595000	20.333016000
1	5.129525000	12.263400000	19.002942000
1	7.709438000	9.343111000	17.070611000
6	10.452828000	12.619887000	14.840015000
6	10.931004000	11.305143000	14.618370000
6	11.248153000	13.679374000	14.336729000
6	12.424590000	13.435685000	13.612625000
6	12.875426000	12.118406000	13.346543000
6	12.106848000	11.061461000	13.892530000
1	10.342591000	10.442723000	14.979422000
1	10.936265000	14.725174000	14.510245000
1	13.005980000	14.297180000	13.237169000
1	12.405107000	10.014465000	13.704104000
6	16.021429000	15.174479000	1.262820000
6	16.412509000	13.814793000	1.190560000
6	16.168470000	15.940813000	0.079815000
6	16.650500000	15.373136000	-1.110611000
6	17.014415000	14.005617000	-1.188580000
6	16.894190000	13.248089000	0.002330000
1	16.300251000	13.166670000	2.077972000
1	15.903469000	17.013664000	0.087418000
1	16.750192000	16.016513000	-2.003604000
1	17.146062000	12.172646000	-0.007095000
6	17.690364000	6.165301000	11.472503000
6	17.403529000	6.838609000	12.686191000
6	17.431796000	6.877022000	10.275349000
6	16.892762000	8.171069000	10.289199000
6	16.578302000	8.834054000	11.501012000
6	16.863331000	8.134562000	12.700102000
5	15.857609000	10.240631000	11.468216000
7	14.886801000	10.626205000	12.464853000
5	14.088190000	11.825400000	12.376319000
7	14.378862000	12.708418000	11.271946000
5	15.319291000	12.394192000	10.223821000
7	16.067748000	11.171495000	10.385119000
1	17.611803000	6.339544000	13.649921000
1	17.619227000	6.392177000	9.300702000
1	16.672923000	8.663215000	9.325082000

1	16.662316000	8.616013000	13.674321000
6	15.422683000	13.285268000	8.922927000
6	15.123150000	14.670565000	8.922245000
6	15.703290000	12.695193000	7.666350000
6	15.638316000	13.433221000	6.475325000
6	15.288858000	14.805764000	6.469255000
6	15.058132000	15.408776000	7.730958000
5	15.039521000	15.564913000	5.105914000
7	15.713327000	15.200628000	3.883517000
7	14.034374000	16.594047000	4.996342000
5	15.371350000	15.739340000	2.588574000
7	14.341728000	16.751469000	2.567594000
5	13.613212000	17.170142000	3.741404000
1	14.928097000	15.186781000	9.879655000
1	15.929998000	11.615570000	7.608767000
1	15.815881000	12.911009000	5.518225000
1	14.813700000	16.485146000	7.784399000
6	-2.019553000	6.284022000	21.198525000
6	-1.211217000	5.610538000	22.148081000
6	-1.351082000	7.134162000	20.283823000
6	0.045989000	7.264040000	20.284591000
6	0.857835000	6.551397000	21.200612000
6	0.185579000	5.740430000	22.149079000
5	2.432637000	6.563351000	21.067252000
7	3.160461000	7.691912000	20.538452000
5	4.566803000	7.652424000	20.215032000
7	5.260511000	6.424558000	20.523713000
5	4.612968000	5.252431000	21.060433000
7	3.209378000	5.384530000	21.368767000
1	-1.686087000	4.960154000	22.904852000
1	-1.932842000	7.672696000	19.514552000
1	0.519121000	7.900163000	19.515084000
1	0.770854000	5.188583000	22.906757000
6	5.352384000	3.862578000	21.198525000
6	6.367484000	3.489532000	20.283823000
6	4.961652000	2.885687000	22.148081000
6	5.516821000	1.597394000	22.149079000
6	6.495835000	1.208644000	21.200612000
6	6.922724000	2.200974000	20.284591000
1	6.699886000	4.209236000	19.514552000
1	4.196357000	3.136335000	22.904852000
1	5.172843000	0.870235000	22.906757000
1	7.673919000	1.947571000	19.515084000
6	-8.357197000	18.397766000	-8.841998000
6	-8.847265000	17.979221000	-7.580566000
6	-7.516067000	19.538418000	-8.850421000
6	-7.149197000	20.190767000	-7.663964000
6	-7.600192000	19.741641000	-6.397339000
6	-8.479569000	18.631244000	-6.393567000
5	-7.053012000	20.353081000	-5.046026000
7	-5.708037000	20.870630000	-4.961036000
5	-5.071841000	21.245302000	-3.721151000

7	-5.895143000	21.182720000	-2.538296000
5	-7.257328000	20.704014000	-2.535065000
7	-7.809141000	20.332623000	-3.816540000
1	-9.496684000	17.087849000	-7.516336000
1	-7.132241000	19.925691000	-9.811580000
1	-6.487038000	21.073361000	-7.725266000
1	-8.850588000	18.231148000	-5.432854000
6	-8.062389000	20.509644000	-1.188580000
6	-9.475434000	20.586127000	-1.110611000
6	-7.379090000	20.161215000	0.002330000
6	-8.066904000	19.878229000	1.190560000
6	-9.480893000	19.926457000	1.262820000
6	-10.164282000	20.303111000	0.079815000
1	-10.056515000	20.879754000	-2.003604000
1	-6.278449000	20.068429000	-0.007095000
1	-7.485192000	19.571184000	2.077972000
1	-11.266514000	20.382609000	0.087418000
6	10.687932000	15.385968000	-11.472503000
6	10.060408000	15.809780000	-10.275349000
6	10.060117000	15.762089000	-12.686191000
6	8.861346000	16.493016000	-12.700102000
6	8.219601000	16.891382000	-11.501012000
6	8.863698000	16.539850000	-10.289199000
5	6.809783000	17.605713000	-11.468216000
7	6.432642000	18.482315000	-10.385119000
5	5.108443000	19.031566000	-10.223821000
7	4.162923000	18.733009000	-11.271946000
5	4.446790000	17.847780000	-12.376319000
7	5.797749000	17.347023000	-12.464853000
1	10.497027000	15.527702000	-9.300702000
1	10.521957000	15.480757000	-13.649921000
1	8.415732000	16.764364000	-13.674321000
1	8.396568000	16.808787000	-9.325082000
6	3.293418000	17.371982000	-13.346543000
6	3.292882000	16.065137000	-13.892530000
6	2.154407000	18.172688000	-13.612625000
6	1.059413000	17.678345000	-14.336729000
6	1.038732000	16.353721000	-14.840015000
6	2.198372000	15.571136000	-14.618370000
1	4.149588000	15.393412000	-13.704104000
1	2.118387000	19.211384000	-13.237169000
1	0.192384000	18.341092000	-14.510245000
1	2.229253000	14.527563000	-14.979422000
6	19.444557000	10.030657000	-3.668174000
6	19.333647000	9.163524000	-2.553668000
6	20.209795000	9.561929000	-4.765103000
6	20.785395000	8.282840000	-4.766875000
6	20.629400000	7.394882000	-3.672991000
6	19.908400000	7.883304000	-2.556060000
1	18.738379000	9.475833000	-1.676993000
1	20.354776000	10.212636000	-5.646411000
1	21.367859000	7.962898000	-5.649832000

1	19.745586000	7.228192000	-1.681395000
6	13.712981000	8.836425000	-14.825926000
6	12.455226000	9.442304000	-14.586631000
6	14.851137000	9.513275000	-14.320200000
6	14.733962000	10.696568000	-13.576472000
6	13.471374000	11.275252000	-13.293575000
6	12.337880000	10.626853000	-13.842473000
5	13.316917000	12.502890000	-12.309651000
7	12.232892000	13.450903000	-12.405392000
5	11.972503000	14.466719000	-11.412824000
7	12.906801000	14.544289000	-10.314781000
5	14.019261000	13.641733000	-10.144445000
7	14.216476000	12.669732000	-11.192703000
1	11.534313000	8.950337000	-14.947972000
1	15.858790000	9.100109000	-14.507461000
1	15.652536000	11.181402000	-13.199268000
1	11.328463000	11.029008000	-13.642377000
6	14.914799000	13.633389000	-8.841998000
6	16.259547000	13.185908000	-8.850421000
6	14.365300000	13.970133000	-7.580566000
6	15.099035000	13.821921000	-6.393567000
6	16.426827000	13.328715000	-6.397339000
6	16.993337000	13.038581000	-7.663964000
5	17.177430000	12.997261000	-5.046026000
7	18.085268000	11.878045000	-4.961036000
7	16.924316000	13.710061000	-3.816540000
5	18.638198000	11.388767000	-3.721151000
7	18.324265000	12.152435000	-2.538296000
5	17.448050000	13.300021000	-2.535065000
1	16.746475000	12.940542000	-9.811580000
1	13.316873000	14.312319000	-7.516336000
1	14.603870000	14.051144000	-5.432854000
1	18.037352000	12.681566000	-7.725266000
6	21.626849000	-3.314251000	3.668174000
6	21.027433000	-3.950586000	2.553668000
6	21.970429000	-4.143256000	4.765103000
6	21.684269000	-5.516391000	4.766875000
6	21.036138000	-6.143072000	3.672991000
6	20.739924000	-5.324137000	2.556060000
1	20.729422000	-3.348033000	1.676993000
1	22.470196000	-3.702041000	5.646411000
1	21.967435000	-6.117593000	5.649832000
1	20.223140000	-5.758434000	1.681395000
6	16.287955000	-0.911470000	14.825926000
6	17.606585000	-1.032878000	14.320200000
6	15.626536000	0.317987000	14.586631000
6	16.227862000	1.345281000	13.842473000
6	17.525997000	1.203595000	13.293575000
6	18.207311000	-0.006700000	13.576472000
5	18.122626000	2.287563000	12.309651000
7	17.802860000	3.691696000	12.405392000
5	18.189283000	4.666561000	11.412824000

7	18.990740000	4.180150000	10.314781000
5	19.360230000	2.796079000	10.144445000
7	18.948453000	1.893794000	11.192703000
1	18.178941000	-1.959420000	14.507461000
1	14.592331000	0.461275000	14.947972000
1	15.647608000	2.263951000	13.642377000
1	19.235431000	-0.154386000	13.199268000
6	20.079831000	2.262945000	8.841998000
6	20.904732000	1.110501000	8.850421000
6	19.833210000	2.858364000	7.580566000
6	20.339697000	2.307179000	6.393567000
6	21.124004000	1.127710000	6.397339000
6	21.411784000	0.560001000	7.663964000
5	21.536431000	0.418365000	5.046026000
7	21.613029000	-1.020714000	4.961036000
7	21.750631000	1.143809000	3.816540000
5	21.772768000	-1.741552000	3.721151000
7	21.967664000	-0.939206000	2.538296000
5	21.933325000	0.504237000	2.535065000
1	21.154442000	0.625787000	9.811580000
1	19.186147000	3.751448000	7.516336000
1	20.073834000	2.783675000	5.432854000
1	22.046562000	-0.342487000	7.725266000
6	15.232327000	-6.041470000	14.840015000
6	14.129696000	-6.902521000	14.618370000
6	16.485728000	-6.470471000	14.336729000
6	16.617505000	-7.664632000	13.612625000
6	15.504014000	-8.500464000	13.346543000
6	14.261297000	-8.096117000	13.892530000
1	13.127656000	-6.609409000	14.979422000
1	17.383965000	-5.850677000	14.510245000
1	17.616495000	-7.951350000	13.237169000
1	13.357712000	-8.703318000	13.704104000
6	3.156812000	-5.804414000	21.200612000
6	4.232489000	-5.903763000	20.284591000
6	3.224003000	-4.753186000	22.149079000
6	4.277686000	-3.827085000	22.148081000
6	5.327508000	-3.896817000	21.198525000
6	5.286403000	-4.977512000	20.283823000
5	6.420844000	-2.764103000	21.060433000
7	6.112745000	-1.388389000	21.368767000
5	6.993844000	-0.285389000	21.067252000
7	8.292079000	-0.628846000	20.538452000
5	8.689108000	-1.978558000	20.215032000
7	7.735705000	-3.017746000	20.523713000
1	4.223621000	-6.696498000	19.515084000
1	2.426139000	-4.650749000	22.906757000
1	4.279578000	-3.021792000	22.904852000
1	6.073600000	-5.071245000	19.514552000
6	10.049005000	-2.294630000	19.473978000
6	10.123867000	-3.370865000	18.556008000
6	11.210825000	-1.494691000	19.611083000

6	12.367744000	-1.739811000	18.854169000
6	12.431316000	-2.800100000	17.916098000
6	11.279122000	-3.616176000	17.800806000
5	13.671219000	-3.043148000	16.966485000
7	13.993217000	-4.360686000	16.472128000
7	14.490603000	-1.957898000	16.480752000
5	15.027482000	-4.620946000	15.501105000
7	15.809866000	-3.482645000	15.083806000
5	15.538785000	-2.127840000	15.502887000
1	9.240223000	-4.013963000	18.395614000
1	11.215865000	-0.657978000	20.333016000
1	13.248297000	-1.088869000	19.002942000
1	11.268174000	-4.444931000	17.070611000
6	-13.524662000	13.037029000	-11.501012000
6	-12.991299000	13.540972000	-10.289199000
6	-12.947484000	13.524263000	-12.700102000
6	-11.881890000	14.438494000	-12.686191000
6	-11.330173000	14.919353000	-11.472503000
6	-11.927158000	14.453507000	-10.275349000
5	-10.058960000	15.856989000	-11.412824000
7	-9.844020000	16.769530000	-10.314781000
5	-8.641869000	17.548637000	-10.144445000
7	-7.656499000	17.435835000	-11.192703000
5	-7.775801000	16.528746000	-12.309651000
7	-9.012397000	15.790729000	-12.405392000
1	-13.391424000	13.179812000	-9.325082000
1	-13.343254000	13.184310000	-13.674321000
1	-11.471611000	14.790793000	-13.649921000
1	-11.523963000	14.781589000	-9.300702000
6	-1.178435000	12.688162000	-17.916098000
6	-2.167184000	12.300054000	-18.854169000
6	-0.046253000	11.844543000	-17.800806000
6	0.077437000	10.670025000	-18.556008000
6	-0.922990000	10.266251000	-19.473978000
6	-2.042800000	11.124013000	-19.611083000
1	-3.058373000	12.936359000	-19.002942000
1	0.745323000	12.090230000	-17.070611000
1	0.962120000	10.028357000	-18.395614000
1	-2.840118000	10.870248000	-20.333016000
6	-5.327508000	3.896817000	-21.198525000
6	-5.286403000	4.977512000	-20.283823000
6	-4.277686000	3.827085000	-22.148081000
6	-3.224003000	4.753186000	-22.149079000
6	-3.156812000	5.804414000	-21.200612000
6	-4.232489000	5.903763000	-20.284591000
5	-1.889796000	6.739731000	-21.067252000
7	-1.964326000	8.080560000	-20.538452000
5	-0.803361000	8.875241000	-20.215032000
7	0.479582000	8.289627000	-20.523713000
5	0.644669000	6.960740000	-21.060433000
7	-0.568506000	6.242601000	-21.368767000
1	-6.073600000	5.071245000	-19.514552000

1	-4.279578000	3.021792000	-22.904852000
1	-2.426139000	4.650749000	-22.906757000
1	-4.223621000	6.696498000	-19.515084000
6	2.059803000	6.270944000	-21.198525000
6	2.317897000	5.250956000	-22.148081000
6	3.100307000	6.565804000	-20.283823000
6	4.306901000	5.849700000	-20.284591000
6	4.544818000	4.795969000	-21.200612000
6	3.524277000	4.535025000	-22.149079000
1	1.551433000	5.003905000	-22.904852000
1	2.946195000	7.343438000	-19.514552000
1	5.063578000	6.086235000	-19.515084000
1	3.673407000	3.744555000	-22.906757000
6	18.604655000	-2.597581000	-11.501012000
6	18.424097000	-3.331019000	-12.700102000
6	18.469365000	-3.318783000	-10.289199000
6	18.144831000	-4.682525000	-10.275349000
6	17.935678000	-5.410302000	-11.472503000
6	18.099385000	-4.696987000	-12.686191000
1	18.544462000	-2.823363000	-13.674321000
1	18.580788000	-2.791410000	-9.325082000
1	18.011482000	-5.184942000	-9.300702000
1	17.974538000	-5.223159000	-13.649921000
6	5.965664000	-2.840342000	-21.200612000
6	6.894301000	-2.288450000	-20.284591000
6	5.402126000	-1.950387000	-22.149079000
6	5.710225000	-0.581816000	-22.148081000
6	6.600536000	-0.021161000	-21.198525000
6	7.202498000	-0.919622000	-20.283823000
5	6.819271000	1.537871000	-21.060433000
7	5.761389000	2.469751000	-21.368767000
5	5.825886000	3.879994000	-21.067252000
7	7.078059000	4.365215000	-20.538452000
5	8.192603000	3.506642000	-20.215032000
7	8.032103000	2.105526000	-20.523713000
1	7.353085000	-2.934999000	-19.515084000
1	4.696429000	-2.336486000	-22.906757000
1	5.238416000	0.070792000	-22.904852000
1	7.894449000	-0.532751000	-19.514552000
6	15.874299000	4.065685000	-14.840015000
6	17.140482000	4.455347000	-14.336729000
6	15.488364000	2.720970000	-14.618370000
6	16.296410000	1.832684000	-13.892530000
6	17.539459000	2.236011000	-13.346543000
6	17.949002000	3.566707000	-13.612625000
5	18.348381000	1.286119000	-12.376319000
7	18.289602000	-0.153462000	-12.464853000
7	19.102564000	1.829643000	-11.271946000
5	18.848367000	-1.036024000	-11.468216000
7	19.565522000	-0.406456000	-10.385119000
5	19.678691000	1.022659000	-10.223821000
1	17.502865000	5.484741000	-14.510245000

1	14.505411000	2.369118000	-14.979422000
1	15.922298000	0.810334000	-13.704104000
1	18.925730000	3.921938000	-13.237169000
6	-15.232327000	6.041470000	-14.840015000
6	-16.485728000	6.470471000	-14.336729000
6	-14.129696000	6.902521000	-14.618370000
6	-14.261297000	8.096117000	-13.892530000
6	-15.504014000	8.500464000	-13.346543000
6	-16.617505000	7.664632000	-13.612625000
5	-15.600114000	9.744416000	-12.376319000
7	-14.706396000	10.874512000	-12.464853000
5	-14.639690000	11.916953000	-11.468216000
7	-15.589930000	11.829155000	-10.385119000
5	-16.521499000	10.739496000	-10.223821000
7	-16.529736000	9.747993000	-11.271946000
1	-17.383965000	5.850677000	-14.510245000
1	-13.127656000	6.609409000	-14.979422000
1	-13.357712000	8.703318000	-13.704104000
1	-17.616495000	7.951350000	-13.237169000
6	-17.400912000	10.562470000	-8.922927000
6	-18.625847000	9.849516000	-8.922245000
6	-16.926430000	11.011686000	-7.666350000
6	-17.608258000	10.721829000	-6.475325000
6	-18.805635000	9.965335000	-6.469255000
6	-19.307835000	9.559561000	-7.730958000
1	-19.056523000	9.504490000	-9.879655000
1	-15.969704000	11.560920000	-7.608767000
1	-17.166475000	11.052075000	-5.518225000
1	-20.255991000	8.994475000	-7.784399000
6	0.922990000	-10.266251000	19.473978000
6	-0.077437000	-10.670025000	18.556008000
6	2.042800000	-11.124013000	19.611083000
6	2.167184000	-12.300054000	18.854169000
6	1.178435000	-12.688162000	17.916098000
6	0.046253000	-11.844543000	17.800806000
1	-0.962120000	-10.028357000	18.395614000
1	2.840118000	-10.870248000	20.333016000
1	3.058373000	-12.936359000	19.002942000
1	-0.745323000	-12.090230000	17.070611000
6	-11.703003000	-5.041616000	17.916098000
6	-11.250537000	-3.704154000	17.800806000
6	-11.028350000	-5.862041000	18.854169000
6	-9.948305000	-5.380327000	19.611083000
6	-9.478565000	-4.050262000	19.473978000
6	-10.171726000	-3.223572000	18.556008000
5	-8.192603000	-3.506642000	20.215032000
7	-8.032103000	-2.105526000	20.523713000
5	-6.819271000	-1.537871000	21.060433000
7	-5.761389000	-2.469751000	21.368767000
5	-5.825886000	-3.879994000	21.067252000
7	-7.078059000	-4.365215000	20.538452000
1	-11.728810000	-3.027242000	17.070611000

1	-11.358119000	-6.906240000	19.002942000
1	-9.460575000	-6.060205000	20.333016000
1	-9.834846000	-2.183903000	18.395614000
6	-4.544818000	-4.795969000	21.200612000
6	-4.306901000	-5.849700000	20.284591000
6	-3.524277000	-4.535025000	22.149079000
6	-2.317897000	-5.250956000	22.148081000
6	-2.059803000	-6.270944000	21.198525000
6	-3.100307000	-6.565804000	20.283823000
5	-0.644669000	-6.960740000	21.060433000
7	-0.479582000	-8.289627000	20.523713000
7	0.568506000	-6.242601000	21.368767000
5	0.803361000	-8.875241000	20.215032000
7	1.964326000	-8.080560000	20.538452000
5	1.889796000	-6.739731000	21.067252000
1	-5.063578000	-6.086235000	19.515084000
1	-3.673407000	-3.744555000	22.906757000
1	-1.551433000	-5.003905000	22.904852000
1	-2.946195000	-7.343438000	19.514552000
6	-3.293418000	-17.371982000	13.346543000
6	-3.292882000	-16.065137000	13.892530000
6	-2.154407000	-18.172688000	13.612625000
6	-1.059413000	-17.678345000	14.336729000
6	-1.038732000	-16.353721000	14.840015000
6	-2.198372000	-15.571136000	14.618370000
5	0.248967000	-15.719936000	15.501105000
7	0.176883000	-14.655867000	16.472128000
5	1.330433000	-13.942487000	16.966485000
7	2.615771000	-14.386406000	16.480752000
5	2.778053000	-15.435801000	15.502887000
7	1.573325000	-16.112273000	15.083806000
1	-4.149588000	-15.393412000	13.704104000
1	-2.118387000	-19.211384000	13.237169000
1	-0.192384000	-18.341092000	14.510245000
1	-2.229253000	-14.527563000	14.979422000
6	4.166395000	-15.772426000	14.825926000
6	5.131288000	-14.763456000	14.586631000
6	4.458409000	-17.064034000	14.320200000
6	5.619997000	-17.318252000	13.576472000
6	6.560518000	-16.296282000	13.293575000
6	6.294123000	-15.017899000	13.842473000
1	4.947977000	-13.735590000	14.947972000
1	3.754082000	-17.894694000	14.507461000
1	5.797245000	-18.341690000	13.199268000
1	6.988522000	-14.182160000	13.642377000
6	8.357197000	-18.397766000	8.841998000
6	8.847265000	-17.979221000	7.580566000
6	7.516067000	-19.538418000	8.850421000
6	7.149197000	-20.190767000	7.663964000
6	7.600192000	-19.741641000	6.397339000
6	8.479569000	-18.631244000	6.393567000
1	9.496684000	-17.087849000	7.516336000

1	7.132241000	-19.925691000	9.811580000
1	6.487038000	-21.073361000	7.725266000
1	8.850588000	-18.231148000	5.432854000
6	18.577876000	-11.853697000	-1.188580000
6	19.766009000	-11.085006000	-1.110611000
6	17.820274000	-11.973445000	0.002330000
6	18.210393000	-11.340218000	1.190560000
6	19.382681000	-10.548113000	1.262820000
6	20.156946000	-10.451147000	0.079815000
5	19.719010000	-9.755299000	2.588574000
7	20.363432000	-8.463305000	2.567594000
5	20.536489000	-7.641068000	3.741404000
7	20.118737000	-8.219640000	4.996342000
5	19.450579000	-9.493612000	5.105914000
7	19.312341000	-10.247010000	3.883517000
1	20.408703000	-10.981004000	-2.003604000
1	16.875298000	-12.545320000	-0.007095000
1	17.559301000	-11.433735000	2.077972000
1	21.095398000	-9.867586000	0.087418000
6	18.805635000	-9.965335000	6.469255000
6	17.608258000	-10.721829000	6.475325000
6	19.307835000	-9.559561000	7.730958000
6	18.625847000	-9.849516000	8.922245000
6	17.400912000	-10.562470000	8.922927000
6	16.926430000	-11.011686000	7.666350000
5	16.521499000	-10.739496000	10.223821000
7	15.589930000	-11.829155000	10.385119000
7	16.529736000	-9.747993000	11.271946000
5	14.639690000	-11.916953000	11.468216000
7	14.706396000	-10.874512000	12.464853000
5	15.600114000	-9.744416000	12.376319000
1	17.166475000	-11.052075000	5.518225000
1	20.255991000	-8.994475000	7.784399000
1	19.056523000	-9.504490000	9.879655000
1	15.969704000	-11.560920000	7.608767000
6	8.772124000	-13.840989000	-14.840015000
6	7.373964000	-13.889484000	-14.618370000
6	9.533987000	-14.924789000	-14.336729000
6	8.938686000	-15.968342000	-13.612625000
6	7.546563000	-15.990051000	-13.346543000
6	6.778853000	-14.932476000	-13.892530000
1	6.735584000	-13.063367000	-14.979422000
1	10.624981000	-14.951336000	-14.510245000
1	9.578357000	-16.787493000	-13.237169000
1	5.690934000	-14.892598000	-13.704104000
6	-0.857835000	-6.551397000	-21.200612000
6	-0.185579000	-5.740430000	-22.149079000
6	-0.045989000	-7.264040000	-20.284591000
6	1.351082000	-7.134162000	-20.283823000
6	2.019553000	-6.284022000	-21.198525000
6	1.211217000	-5.610538000	-22.148081000
5	3.569873000	-6.010284000	-21.060433000

7	4.129240000	-4.716211000	-21.368767000
5	5.490391000	-4.341762000	-21.067252000
7	6.338807000	-5.382709000	-20.538452000
5	5.866668000	-6.708017000	-20.215032000
7	4.484530000	-6.988341000	-20.523713000
1	-0.519121000	-7.900163000	-19.515084000
1	1.932842000	-7.672696000	-19.514552000
1	1.686087000	-4.960154000	-22.904852000
6	6.781066000	-7.763051000	-19.473978000
6	8.191191000	-7.798788000	-19.611083000
6	6.209036000	-8.677747000	-18.556008000
6	6.999467000	-9.555250000	-17.800806000
6	8.411288000	-9.572272000	-17.916098000
6	8.983080000	-8.677114000	-18.854169000
5	9.271531000	-10.497700000	-16.966485000
7	8.757603000	-11.752876000	-16.472128000
7	10.572321000	-10.101335000	-16.480752000
5	9.441365000	-12.571356000	-15.501105000
7	10.743403000	-12.110326000	-15.083806000
5	11.320428000	-10.854927000	-15.502887000
1	8.687076000	-7.124836000	-20.333016000
1	5.116150000	-8.678631000	-18.395614000
1	6.503480000	-10.219292000	-17.070611000
1	10.078076000	-8.668068000	-19.002942000
5	6.893133000	-17.052914000	-12.376319000
7	7.643111000	-17.602228000	-11.271946000
7	5.505847000	-17.441868000	-12.464853000
5	4.839148000	-18.246011000	-11.468216000
7	5.659516000	-18.733519000	-10.385119000
5	7.053656000	-18.399528000	-10.223821000
6	7.869170000	-18.773217000	-8.922927000
6	7.221263000	-18.857747000	-7.666350000
6	9.279227000	-18.916424000	-8.922245000
6	10.001398000	-19.082708000	-7.730958000
6	9.356601000	-19.115800000	-6.469255000
6	7.943247000	-19.024016000	-6.475325000
5	10.155644000	-19.113257000	-5.105914000
7	11.445017000	-18.475325000	-4.996342000
5	12.123061000	-18.252799000	-3.741404000
7	11.499756000	-18.816283000	-2.567594000
5	10.218994000	-19.482746000	-2.588574000
7	9.600971000	-19.641514000	-3.883517000
1	6.124424000	-18.739737000	-7.608767000
1	9.830452000	-18.890437000	-9.879655000
1	11.100621000	-19.182856000	-7.784399000
1	7.391723000	-19.031518000	-5.518225000
6	-6.084013000	-20.536866000	-7.663964000
6	-4.052821000	-19.796343000	-8.841998000
6	-3.410333000	-19.745787000	-7.580566000
6	-5.455160000	-20.438604000	-6.397339000
6	-4.091055000	-20.057159000	-6.393567000
5	-3.323411000	-19.276709000	-10.144445000

7	-1.892903000	-19.353004000	-10.314781000
7	-4.054289000	-18.606264000	-11.192703000
5	-6.257234000	-20.611645000	-5.046026000
7	-5.633488000	-21.039536000	-3.816540000
5	-6.298213000	-21.015649000	-2.535065000
7	-7.681620000	-20.602259000	-2.538296000
5	-8.384470000	-20.168964000	-3.721151000
7	-7.649550000	-20.239794000	-4.961036000
6	-4.271144000	-17.040145000	-13.293575000
6	-3.735247000	-15.849328000	-13.842473000
6	-5.632740000	-17.314111000	-13.576472000
6	-6.423059000	-16.425681000	-14.320200000
6	-5.900114000	-15.209106000	-14.825926000
6	-4.526442000	-14.959982000	-14.586631000
5	-1.182635000	-18.741083000	-11.412824000
7	-1.990375000	-18.072322000	-12.405392000
5	-3.424598000	-17.942538000	-12.309651000
6	0.396927000	-18.729719000	-11.472503000
6	1.153715000	-18.703740000	-10.275349000
6	2.550998000	-18.590970000	-10.289199000
6	3.278708000	-18.496775000	-11.501012000
6	1.125917000	-18.664986000	-12.686191000
6	2.525372000	-18.551699000	-12.700102000
6	-5.532656000	-21.331604000	-1.188580000
6	-4.434435000	-22.224047000	-1.110611000
6	-5.880655000	-20.648085000	0.002330000
6	-5.157867000	-20.823433000	1.190560000
6	-4.042274000	-21.693571000	1.262820000
6	-3.710792000	-22.399977000	0.079815000
1	-7.138487000	-20.861692000	-7.725266000
1	-2.361006000	-19.406371000	-7.516336000
1	-3.555724000	-19.951553000	-5.432854000
1	-2.682231000	-15.581359000	-13.642377000
1	-6.090905000	-18.246274000	-13.199268000
1	-7.481121000	-16.683706000	-14.507461000
1	0.634681000	-18.732172000	-9.300702000
1	3.086990000	-18.533973000	-9.325082000
1	0.586918000	-18.708847000	-13.649921000
1	3.045376000	-18.509299000	-13.674321000
1	-4.136920000	-22.803147000	-2.003604000
1	-6.716554000	-19.926080000	-0.007095000
1	-5.448006000	-20.233106000	2.077972000
1	-2.865796000	-23.112168000	0.087418000
6	-5.403768000	-20.224746000	-8.850421000
1	-5.941923000	-20.312449000	-9.811580000
5	-3.018402000	-21.432288000	5.105914000
7	-3.777644000	-21.533628000	3.883517000
7	-1.600311000	-21.674064000	4.996342000
5	-0.920963000	-21.892581000	3.741404000
5	-3.184331000	-21.768446000	2.588574000
7	-1.756435000	-21.982080000	2.567594000
6	0.658115000	-21.904870000	3.672991000

6	1.454408000	-22.327624000	4.766875000
6	2.848765000	-22.175456000	4.765103000
6	3.531024000	-21.592516000	3.668174000
6	2.740604000	-21.219075000	2.553668000
6	1.345434000	-21.370089000	2.556060000
1	0.970134000	-22.782712000	5.649832000
1	3.422822000	-22.514420000	5.646411000
1	3.221575000	-20.749451000	1.676993000
1	0.772697000	-21.012803000	1.681395000
6	-3.666336000	-20.964680000	6.469255000
6	-4.755814000	-20.059676000	6.475325000
6	-3.125234000	-21.316909000	7.730958000
6	-3.611743000	-20.757901000	8.922245000
6	-4.668328000	-19.813234000	8.922927000
6	-5.242181000	-19.500790000	7.666350000
1	-5.206416000	-19.741567000	5.518225000
1	-2.294809000	-22.044038000	7.784399000
1	-3.150518000	-21.060879000	9.879655000
1	-6.060178000	-18.760612000	7.608767000
6	-9.835104000	-19.544196000	-3.668174000
6	-10.729707000	-19.614783000	-4.765103000
6	-10.255065000	-18.777479000	-2.553668000
6	-11.472544000	-18.079591000	-2.556060000
6	-11.947207000	-18.918307000	-4.766875000
6	-12.342933000	-18.108242000	-3.672991000
1	-10.464523000	-20.226433000	-5.646411000
1	-11.725890000	-17.453895000	-1.681395000
1	-12.606487000	-19.001832000	-5.649832000
5	-4.446790000	-17.847780000	12.376319000
7	-4.162923000	-18.733009000	11.271946000
7	-5.797749000	-17.347023000	12.464853000
6	-14.914799000	-13.633389000	8.841998000
6	-16.259547000	-13.185908000	8.850421000
6	-14.365300000	-13.970133000	7.580566000
6	-15.099035000	-13.821921000	6.393567000
6	-16.426827000	-13.328715000	6.397339000
6	-16.993337000	-13.038581000	7.663964000
5	-17.177430000	-12.997261000	5.046026000
7	-18.085268000	-11.878045000	4.961036000
7	-16.924316000	-13.710061000	3.816540000
5	-17.448050000	-13.300021000	2.535065000
5	-18.638198000	-11.388767000	3.721151000
7	-18.324265000	-12.152435000	2.538296000
6	-19.444557000	-10.030657000	3.668174000
6	-20.209795000	-9.561929000	4.765103000
6	-19.333647000	-9.163524000	2.553668000
6	-19.908400000	-7.883304000	2.556060000
6	-20.629400000	-7.394882000	3.672991000
6	-20.785395000	-8.282840000	4.766875000
5	-21.105676000	-5.889291000	3.741404000
7	-21.107783000	-5.175668000	4.996342000
7	-21.448968000	-5.122367000	2.567594000

5	-21.687035000	-3.698341000	2.588574000
7	-21.647053000	-3.061504000	3.883517000
5	-21.316055000	-3.752270000	5.105914000
5	-5.108443000	-19.031566000	10.223821000
7	-6.432642000	-18.482315000	10.385119000
5	-6.809783000	-17.605713000	11.468216000
6	-17.014415000	-14.005617000	1.188580000
6	-16.894190000	-13.248089000	-0.002330000
6	-16.412509000	-13.814793000	-1.190560000
6	-16.021429000	-15.174479000	-1.262820000
6	-16.168470000	-15.940813000	-0.079815000
6	-16.650500000	-15.373136000	1.110611000
1	-16.746475000	-12.940542000	9.811580000
1	-13.316873000	-14.312319000	7.516336000
1	-14.603870000	-14.051144000	5.432854000
1	-18.037352000	-12.681566000	7.725266000
1	-20.354776000	-10.212636000	5.646411000
1	-18.738379000	-9.475833000	1.676993000
1	-19.745586000	-7.228192000	1.681395000
1	-21.367859000	-7.962898000	5.649832000
1	-17.146062000	-12.172646000	0.007095000
1	-16.300251000	-13.166670000	-2.077972000
1	-15.903469000	-17.013664000	-0.087418000
1	-16.750192000	-16.016513000	2.003604000
6	-17.606585000	1.032878000	-14.320200000
6	-17.525997000	-1.203595000	-13.293575000
6	-16.227862000	-1.345281000	-13.842473000
6	-16.287955000	0.911470000	-14.825926000
6	-15.626536000	-0.317987000	-14.586631000
5	-18.122626000	-2.287563000	-12.309651000
7	-18.948453000	-1.893794000	-11.192703000
7	-17.802860000	-3.691696000	-12.405392000
5	-15.538785000	2.127840000	-15.502887000
7	-15.809866000	3.482645000	-15.083806000
5	-15.027482000	4.620946000	-15.501105000
7	-13.993217000	4.360686000	-16.472128000
5	-13.671219000	3.043148000	-16.966485000
7	-14.490603000	1.957898000	-16.480752000
6	-17.690364000	-6.165301000	-11.472503000
6	-17.403529000	-6.838609000	-12.686191000
6	-17.431796000	-6.877022000	-10.275349000
6	-16.892762000	-8.171069000	-10.289199000
6	-16.578302000	-8.834054000	-11.501012000
6	-16.863331000	-8.134562000	-12.700102000
5	-15.857609000	-10.240631000	-11.468216000
7	-14.886801000	-10.626205000	-12.464853000
7	-16.067748000	-11.171495000	-10.385119000
5	-15.319291000	-12.394192000	-10.223821000
5	-14.088190000	-11.825400000	-12.376319000
7	-14.378862000	-12.708418000	-11.271946000
5	-19.360230000	-2.796079000	-10.144445000
7	-18.990740000	-4.180150000	-10.314781000

5	-18.189283000	-4.666561000	-11.412824000
6	-15.422683000	-13.285268000	-8.922927000
6	-15.123150000	-14.670565000	-8.922245000
6	-15.058132000	-15.408776000	-7.730958000
6	-15.288858000	-14.805764000	-6.469255000
6	-15.638316000	-13.433221000	-6.475325000
6	-15.703290000	-12.695193000	-7.666350000
6	-20.079831000	-2.262945000	-8.841998000
6	-19.833210000	-2.858364000	-7.580566000
6	-20.339697000	-2.307179000	-6.393567000
6	-21.124004000	-1.127710000	-6.397339000
6	-20.904732000	-1.110501000	-8.850421000
6	-21.411784000	-0.560001000	-7.663964000
1	-18.178941000	1.959420000	-14.507461000
1	-15.647608000	-2.263951000	-13.642377000
1	-14.592331000	-0.461275000	-14.947972000
1	-17.611803000	-6.339544000	-13.649921000
1	-17.619227000	-6.392177000	-9.300702000
1	-16.672923000	-8.663215000	-9.325082000
1	-16.662316000	-8.616013000	-13.674321000
1	-14.928097000	-15.186781000	-9.879655000
1	-14.813700000	-16.485146000	-7.784399000
1	-15.929998000	-11.615570000	-7.608767000
1	-19.186147000	-3.751448000	-7.516336000
1	-20.073834000	-2.783675000	-5.432854000
1	-21.154442000	-0.625787000	-9.811580000
1	-22.046562000	0.342487000	-7.725266000
6	-18.207311000	0.006700000	-13.576472000
1	-19.235431000	0.154386000	-13.199268000
5	-15.371350000	-15.739340000	-2.588574000
7	-15.713327000	-15.200628000	-3.883517000
5	-15.039521000	-15.564913000	-5.105914000
7	-14.341728000	-16.751469000	-2.567594000
7	-14.034374000	-16.594047000	-4.996342000
5	-13.613212000	-17.170142000	-3.741404000
6	-6.495835000	-1.208644000	-21.200612000
6	-6.922724000	-2.200974000	-20.284591000
6	-5.516821000	-1.597394000	-22.149079000
6	-4.961652000	-2.885687000	-22.148081000
6	-6.367484000	-3.489532000	-20.283823000
6	-5.352384000	-3.862578000	-21.198525000
6	-12.875426000	-12.118406000	-13.346543000
6	-12.106848000	-11.061461000	-13.892530000
6	-12.424590000	-13.435685000	-13.612625000
6	-11.248153000	-13.679374000	-14.336729000
6	-10.452828000	-12.619887000	-14.840015000
6	-10.931004000	-11.305143000	-14.618370000
5	-9.038528000	-12.864034000	-15.501105000
7	-8.471401000	-11.960814000	-16.472128000
7	-8.197710000	-13.959880000	-15.083806000
5	-6.825444000	-14.120724000	-15.502887000
7	-6.339914000	-13.176359000	-16.480752000

5	-7.118845000	-12.061718000	-16.966485000
6	-6.504541000	-10.957605000	-17.916098000
6	-5.476502000	-11.224792000	-18.854169000
6	-6.924628000	-9.609623000	-17.800806000
6	-6.334331000	-8.586715000	-18.556008000
6	-5.287636000	-8.848092000	-19.473978000
6	-4.885871000	-10.200243000	-19.611083000
5	-4.566803000	-7.652424000	-20.215032000
7	-5.260511000	-6.424558000	-20.523713000
5	-4.612968000	-5.252431000	-21.060433000
7	-3.209378000	-5.384530000	-21.368767000
5	-2.432637000	-6.563351000	-21.067252000
7	-3.160461000	-7.691912000	-20.538452000
1	-7.673919000	-1.947571000	-19.515084000
1	-5.172843000	-0.870235000	-22.906757000
1	-4.196357000	-3.136335000	-22.904852000
1	-6.699886000	-4.209236000	-19.514552000
1	-12.405107000	-10.014465000	-13.704104000
1	-13.005980000	-14.297180000	-13.237169000
1	-10.936265000	-14.725174000	-14.510245000
1	-10.342591000	-10.442723000	-14.979422000
1	-5.129525000	-12.263400000	-19.002942000
1	-7.709438000	-9.343111000	-17.070611000
1	-6.672892000	-7.547592000	-18.395614000
1	-4.091667000	-10.463595000	-20.333016000
1	-15.815881000	-12.911009000	-5.518225000
6	9.480893000	-19.926457000	-1.262820000
6	8.066904000	-19.878229000	-1.190560000
6	10.164282000	-20.303111000	-0.079815000
6	9.475434000	-20.586127000	1.110611000
6	8.062389000	-20.509644000	1.188580000
6	7.379090000	-20.161215000	-0.002330000
5	7.257328000	-20.704014000	2.535065000
7	5.895143000	-21.182720000	2.538296000
7	7.809141000	-20.332623000	3.816540000
5	7.053012000	-20.353081000	5.046026000
5	5.071841000	-21.245302000	3.721151000
7	5.708037000	-20.870630000	4.961036000
1	7.485192000	-19.571184000	-2.077972000
1	11.266514000	-20.382609000	-0.087418000
1	10.056515000	-20.879754000	2.003604000
1	6.278449000	-20.068429000	0.007095000
6	-8.219601000	-16.891382000	11.501012000
6	-8.863698000	-16.539850000	10.289199000
6	-8.861346000	-16.493016000	12.700102000
6	-10.060117000	-15.762089000	12.686191000
6	-10.687932000	-15.385968000	11.472503000
6	-10.060408000	-15.809780000	10.275349000
5	-11.972503000	-14.466719000	11.412824000
7	-12.906801000	-14.544289000	10.314781000
7	-12.232892000	-13.450903000	12.405392000
5	-13.316917000	-12.502890000	12.309651000

7	-14.216476000	-12.669732000	11.192703000
5	-14.019261000	-13.641733000	10.144445000
6	-21.880943000	-2.859251000	1.262820000
6	-22.450342000	-3.392800000	0.079815000
6	-21.398130000	-1.529371000	1.190560000
6	-21.454719000	-0.787774000	0.002330000
6	-21.997246000	-1.329959000	-1.188580000
6	-22.506640000	-2.650210000	-1.110611000
5	-21.933325000	-0.504237000	-2.535065000
7	-21.750631000	-1.143809000	-3.816540000
7	-21.967664000	0.939206000	-2.538296000
5	-21.772768000	1.741552000	-3.721151000
7	-21.613029000	1.020714000	-4.961036000
5	-21.536431000	-0.418365000	-5.046026000
6	-12.431316000	2.800100000	-17.916098000
6	-12.367744000	1.739811000	-18.854169000
6	-11.279122000	3.616176000	-17.800806000
6	-11.210825000	1.494691000	-19.611083000
6	-10.049005000	2.294630000	-19.473978000
6	-10.123867000	3.370865000	-18.556008000
5	-8.689108000	1.978558000	-20.215032000
7	-8.292079000	0.628846000	-20.538452000
7	-7.735705000	3.017746000	-20.523713000
5	-6.420844000	2.764103000	-21.060433000
7	-6.112745000	1.388389000	-21.368767000
5	-6.993844000	0.285389000	-21.067252000
1	-8.396568000	-16.808787000	9.325082000
1	-8.415732000	-16.764364000	13.674321000
1	-10.521957000	-15.480757000	13.649921000
1	-10.497027000	-15.527702000	9.300702000
1	-22.866557000	-4.416519000	0.087418000
1	-20.926354000	-1.071012000	2.077972000
1	-21.026357000	0.230325000	-0.007095000
1	-22.965460000	-3.112115000	-2.003604000
1	-13.248297000	1.088869000	-19.002942000
1	-11.268174000	4.444931000	-17.070611000
1	-11.215865000	0.657978000	-20.333016000
1	-9.240223000	4.013963000	-18.395614000
6	15.548420000	-15.393229000	-3.668174000
6	14.689454000	-15.555706000	-2.553668000
6	15.339105000	-16.265859000	-4.765103000
6	14.300489000	-17.208547000	-4.766875000
6	13.407786000	-17.334581000	-3.672991000
6	13.649502000	-16.497939000	-2.556060000
1	14.802530000	-14.893064000	-1.676993000
1	16.002766000	-16.202664000	-5.646411000
1	14.176197000	-17.861371000	-5.649832000
1	12.976141000	-16.545534000	-1.681395000
6	12.641484000	-10.311215000	-14.825926000
6	12.829041000	-8.927791000	-14.586631000
6	13.636916000	-11.184507000	-14.320200000
6	14.726086000	-10.707409000	-13.576472000

6	14.886285000	-9.327794000	-13.293575000
6	13.919352000	-8.450143000	-13.842473000
5	16.006108000	-8.801535000	-12.309651000
7	16.572740000	-7.477614000	-12.405392000
5	17.458374000	-6.916065000	-11.412824000
7	17.820862000	-7.780665000	-10.314781000
5	17.306249000	-9.117582000	-10.144445000
7	16.442764000	-9.605510000	-11.192703000
1	12.076575000	-8.203977000	-14.947972000
1	13.555354000	-12.270518000	-14.507461000
1	15.471045000	-11.431204000	-13.199268000
1	13.989898000	-7.365858000	-13.642377000
6	17.575050000	-9.971868000	-8.841998000
6	17.725508000	-9.345203000	-7.580566000
6	17.565020000	-11.389079000	-8.850421000
6	17.651657000	-12.132481000	-7.663964000
6	17.752530000	-11.504042000	-6.397339000
6	17.811286000	-10.088827000	-6.393567000
5	17.669248000	-12.320332000	-5.046026000
7	16.885347000	-13.529594000	-4.961036000
7	18.268944000	-11.859340000	-3.816540000
5	16.590881000	-14.206657000	-3.721151000
7	17.220162000	-13.672102000	-2.538296000
5	18.040815000	-12.484149000	-2.535065000
1	17.726964000	-8.242349000	-7.516336000
1	17.482132000	-11.927997000	-9.811580000
1	17.634735000	-13.235722000	-7.725266000
1	17.876276000	-9.547063000	-5.432854000
6	13.524662000	-13.037029000	11.501012000
6	12.991299000	-13.540972000	10.289199000
6	11.927158000	-14.453507000	10.275349000
6	11.330173000	-14.919353000	11.472503000
6	11.881890000	-14.438494000	12.686191000
6	12.947484000	-13.524263000	12.700102000
5	10.058960000	-15.856989000	11.412824000
7	9.012397000	-15.790729000	12.405392000
5	7.775801000	-16.528746000	12.309651000
7	7.656499000	-17.435835000	11.192703000
5	8.641869000	-17.548637000	10.144445000
7	9.844020000	-16.769530000	10.314781000
1	13.391424000	-13.179812000	9.325082000
1	11.523963000	-14.781589000	9.300702000
1	11.471611000	-14.790793000	13.649921000
1	13.343254000	-13.184310000	13.674321000
5	-12.848966000	-5.573782000	16.966485000
7	-13.883898000	-4.697137000	16.472128000
5	-14.873612000	-5.094509000	15.501105000
7	-14.837498000	-6.475287000	15.083806000
5	-13.821854000	-7.412010000	15.502887000
7	-12.873968000	-6.933390000	16.480752000
6	-13.712981000	-8.836425000	14.825926000
6	-12.455226000	-9.442304000	14.586631000

6	-14.851137000	-9.513275000	14.320200000
6	-14.733962000	-10.696568000	13.576472000
6	-13.471374000	-11.275252000	13.293575000
6	-12.337880000	-10.626853000	13.842473000
1	-11.534313000	-8.950337000	14.947972000
1	-15.858790000	-9.100109000	14.507461000
1	-15.652536000	-11.181402000	13.199268000
1	-11.328463000	-11.029008000	13.642377000
6	-21.071555000	-2.991550000	6.469255000
6	-21.239336000	-3.615014000	7.730958000
6	-20.547513000	-1.675733000	6.475325000
6	-20.166276000	-1.040465000	7.666350000
6	-20.286098000	-1.682782000	8.922927000
6	-20.858027000	-2.979572000	8.922245000
5	-19.678691000	-1.022659000	10.223821000
7	-19.565522000	0.406456000	10.385119000
5	-18.848367000	1.036024000	11.468216000
7	-18.289602000	0.153462000	12.464853000
5	-18.348381000	-1.286119000	12.376319000
7	-19.102564000	-1.829643000	11.271946000
1	-21.674261000	-4.629489000	7.784399000
1	-20.384217000	-1.148884000	5.518225000
1	-19.715100000	-0.033776000	7.608767000
1	-21.003650000	-3.511849000	9.879655000
6	-17.539459000	-2.236011000	13.346543000
6	-17.949002000	-3.566707000	13.612625000
6	-16.296410000	-1.832684000	13.892530000
6	-15.488364000	-2.720970000	14.618370000
6	-15.874299000	-4.065685000	14.840015000
6	-17.140482000	-4.455347000	14.336729000
1	-18.925730000	-3.921938000	13.237169000
1	-15.922298000	-0.810334000	13.704104000
1	-14.505411000	-2.369118000	14.979422000
1	-17.502865000	-5.484741000	14.510245000
6	-17.752530000	11.504042000	6.397339000
6	-17.811286000	10.088827000	6.393567000
6	-17.651657000	12.132481000	7.663964000
6	-17.565020000	11.389079000	8.850421000
6	-17.725508000	9.345203000	7.580566000
1	-17.876276000	9.547063000	5.432854000
1	-17.634735000	13.235722000	7.725266000
1	-17.482132000	11.927997000	9.811580000
1	-17.726964000	8.242349000	7.516336000
6	-21.626849000	3.314251000	-3.668174000
6	-21.970429000	4.143256000	-4.765103000
6	-21.027433000	3.950586000	-2.553668000
6	-20.739924000	5.324137000	-2.556060000
6	-21.036138000	6.143072000	-3.672991000
6	-21.684269000	5.516391000	-4.766875000
5	-20.536489000	7.641068000	-3.741404000
7	-20.118737000	8.219640000	-4.996342000
5	-19.450579000	9.493612000	-5.105914000

7	-19.312341000	10.247010000	-3.883517000
5	-19.719010000	9.755299000	-2.588574000
7	-20.363432000	8.463305000	-2.567594000
1	-22.470196000	3.702041000	-5.646411000
1	-20.729422000	3.348033000	-1.676993000
1	-20.223140000	5.758434000	-1.681395000
1	-21.967435000	6.117593000	-5.649832000
6	-19.382681000	10.548113000	-1.262820000
6	-20.156946000	10.451147000	-0.079815000
6	-18.210393000	11.340218000	-1.190560000
6	-17.820274000	11.973445000	-0.002330000
6	-18.577876000	11.853697000	1.188580000
6	-19.766009000	11.085006000	1.110611000
5	-18.040815000	12.484149000	2.535065000
7	-18.268944000	11.859340000	3.816540000
7	-17.220162000	13.672102000	2.538296000
5	-17.669248000	12.320332000	5.046026000
7	-16.885347000	13.529594000	4.961036000
5	-16.590881000	14.206657000	3.721151000
1	-21.095398000	9.867586000	-0.087418000
1	-17.559301000	11.433735000	-2.077972000
1	-16.875298000	12.545320000	0.007095000
1	-20.408703000	10.981004000	2.003604000
6	-17.575050000	9.971868000	8.841998000
5	-17.306249000	9.117582000	10.144445000
7	-16.442764000	9.605510000	11.192703000
5	-16.006108000	8.801535000	12.309651000
7	-16.572740000	7.477614000	12.405392000
5	-17.458374000	6.916065000	11.412824000
7	-17.820862000	7.780665000	10.314781000
6	-17.935678000	5.410302000	11.472503000
6	-18.144831000	4.682525000	10.275349000
6	-18.099385000	4.696987000	12.686191000
6	-18.424097000	3.331019000	12.700102000
6	-18.604655000	2.597581000	11.501012000
6	-18.469365000	3.318783000	10.289199000
1	-18.011482000	5.184942000	9.300702000
1	-17.974538000	5.223159000	13.649921000
1	-18.544462000	2.823363000	13.674321000
1	-18.580788000	2.791410000	9.325082000
5	14.873612000	5.094509000	-15.501105000
7	14.837498000	6.475287000	-15.083806000
7	13.883898000	4.697137000	-16.472128000
5	13.821854000	7.412010000	-15.502887000
7	12.873968000	6.933390000	-16.480752000
5	12.848966000	5.573782000	-16.966485000
6	11.703003000	5.041616000	-17.916098000
6	11.250537000	3.704154000	-17.800806000
6	11.028350000	5.862041000	-18.854169000
6	10.171726000	3.223572000	-18.556008000
6	9.948305000	5.380327000	-19.611083000
6	9.478565000	4.050262000	-19.473978000

1	11.728810000	3.027242000	-17.070611000
1	11.358119000	6.906240000	-19.002942000
1	9.834846000	2.183903000	-18.395614000
1	9.460575000	6.060205000	-20.333016000
5	-1.330433000	13.942487000	-16.966485000
7	-2.615771000	14.386406000	-16.480752000
7	-0.176883000	14.655867000	-16.472128000
5	-0.248967000	15.719936000	-15.501105000
7	-1.573325000	16.112273000	-15.083806000
5	-2.778053000	15.435801000	-15.502887000
6	-4.166395000	15.772426000	-14.825926000
6	-5.131288000	14.763456000	-14.586631000
6	-4.458409000	17.064034000	-14.320200000
6	-5.619997000	17.318252000	-13.576472000
6	-6.560518000	16.296282000	-13.293575000
6	-6.294123000	15.017899000	-13.842473000
1	-4.947977000	13.735590000	-14.947972000
1	-3.754082000	17.894694000	-14.507461000
1	-5.797245000	18.341690000	-13.199268000
1	-6.988522000	14.182160000	-13.642377000
6	21.997246000	1.329959000	1.188580000
6	22.506640000	2.650210000	1.110611000
6	21.454719000	0.787774000	-0.002330000
6	21.398130000	1.529371000	-1.190560000
6	21.880943000	2.859251000	-1.262820000
6	22.450342000	3.392800000	-0.079815000
5	21.687035000	3.698341000	-2.588574000
7	21.448968000	5.122367000	-2.567594000
7	21.647053000	3.061504000	-3.883517000
5	21.105676000	5.889291000	-3.741404000
7	21.107783000	5.175668000	-4.996342000
5	21.316055000	3.752270000	-5.105914000
6	21.071555000	2.991550000	-6.469255000
6	21.239336000	3.615014000	-7.730958000
6	20.547513000	1.675733000	-6.475325000
6	20.166276000	1.040465000	-7.666350000
6	20.286098000	1.682782000	-8.922927000
6	20.858027000	2.979572000	-8.922245000
1	22.965460000	3.112115000	2.003604000
1	21.026357000	-0.230325000	0.007095000
1	20.926354000	1.071012000	-2.077972000
1	22.866557000	4.416519000	-0.087418000
1	21.674261000	4.629489000	-7.784399000
1	20.384217000	1.148884000	-5.518225000
1	19.715100000	0.033776000	-7.608767000
1	21.003650000	3.511849000	-9.879655000
5	-10.218994000	19.482746000	2.588574000
7	-9.600971000	19.641514000	3.883517000
7	-11.499756000	18.816283000	2.567594000
5	-12.123061000	18.252799000	3.741404000
7	-11.445017000	18.475325000	4.996342000
5	-10.155644000	19.113257000	5.105914000

6	-14.300489000	17.208547000	4.766875000
6	-13.649502000	16.497939000	2.556060000
6	-14.689454000	15.555706000	2.553668000
6	-15.339105000	16.265859000	4.765103000
6	-15.548420000	15.393229000	3.668174000
6	-13.407786000	17.334581000	3.672991000
1	-14.176197000	17.861371000	5.649832000
1	-12.976141000	16.545534000	1.681395000
1	-14.802530000	14.893064000	1.676993000
1	-16.002766000	16.202664000	5.646411000
1	-0.770854000	-5.188583000	-22.906757000
6	-5.965664000	2.840342000	21.200612000
6	-5.402126000	1.950387000	22.149079000
6	-6.894301000	2.288450000	20.284591000
6	-7.202498000	0.919622000	20.283823000
6	-6.600536000	0.021161000	21.198525000
6	-5.710225000	0.581816000	22.148081000
1	-4.696429000	2.336486000	22.906757000
1	-7.353085000	2.934999000	19.515084000
1	-7.894449000	0.532751000	19.514552000
1	-5.238416000	-0.070792000	22.904852000
6	-14.886285000	9.327794000	13.293575000
6	-14.726086000	10.707409000	13.576472000
6	-13.919352000	8.450143000	13.842473000
6	-12.829041000	8.927791000	14.586631000
6	-12.641484000	10.311215000	14.825926000
6	-13.636916000	11.184507000	14.320200000
5	-11.320428000	10.854927000	15.502887000
7	-10.743403000	12.110326000	15.083806000
5	-9.441365000	12.571356000	15.501105000
7	-8.757603000	11.752876000	16.472128000
5	-9.271531000	10.497700000	16.966485000
7	-10.572321000	10.101335000	16.480752000
1	-13.989898000	7.365858000	13.642377000
1	-12.076575000	8.203977000	14.947972000
1	-13.555354000	12.270518000	14.507461000
6	-8.411288000	9.572272000	17.916098000
6	-6.999467000	9.555250000	17.800806000
6	-8.983080000	8.677114000	18.854169000
6	-8.191191000	7.798788000	19.611083000
6	-6.781066000	7.763051000	19.473978000
6	-6.209036000	8.677747000	18.556008000
5	-5.866668000	6.708017000	20.215032000
7	-6.338807000	5.382709000	20.538452000
7	-4.484530000	6.988341000	20.523713000
5	-5.490391000	4.341762000	21.067252000
7	-4.129240000	4.716211000	21.368767000
5	-3.569873000	6.010284000	21.060433000
1	-6.503480000	10.219292000	17.070611000
1	-10.078076000	8.668068000	19.002942000
1	-8.687076000	7.124836000	20.333016000
1	-5.116150000	8.678631000	18.395614000

1	-15.471045000	11.431204000	13.199268000
1	-4.070579000	-14.020673000	-14.947972000
1	-9.589912000	-18.680253000	-1.676993000
6	-9.356601000	19.115800000	6.469255000
6	-7.943247000	19.024016000	6.475325000
6	-7.221263000	18.857747000	7.666350000
6	-7.869170000	18.773217000	8.922927000
6	-9.279227000	18.916424000	8.922245000
6	-10.001398000	19.082708000	7.730958000
1	-7.391723000	19.031518000	5.518225000
1	-6.124424000	18.739737000	7.608767000
1	-9.830452000	18.890437000	9.879655000
1	-11.100621000	19.182856000	7.784399000
6	-3.531024000	21.592516000	-3.668174000
6	-2.740604000	21.219075000	-2.553668000
6	-2.848765000	22.175456000	-4.765103000
6	-1.345434000	21.370089000	-2.556060000
6	-0.658115000	21.904870000	-3.672991000
6	-1.454408000	22.327624000	-4.766875000
1	-3.221575000	20.749451000	-1.676993000
1	-3.422822000	22.514420000	-5.646411000
1	-0.772697000	21.012803000	-1.681395000
1	-0.970134000	22.782712000	-5.649832000
1	-8.599704000	17.262284000	11.131225000
1	-5.223485000	19.343067000	9.686520000
1	-4.941954000	17.054375000	13.227766000
1	4.794834000	21.422121000	-3.934701000
1	1.302231000	22.119062000	-1.659232000
1	1.022676000	21.506099000	-5.826097000
1	1.512162000	17.600911000	13.179465000
1	1.349757000	19.872641000	9.618488000
1	5.056666000	18.447837000	11.049187000
1	8.087626000	19.840803000	5.797260000
1	4.641921000	21.297167000	3.840357000
1	8.192306000	20.617380000	1.650127000
1	5.365704000	13.255100000	16.788807000
1	8.534425000	14.555475000	14.320701000
1	9.061841000	11.205258000	16.833157000
1	14.692526000	9.970170000	13.227766000
1	13.759953000	13.513144000	11.131225000
1	16.782205000	10.945166000	9.686520000
1	16.470708000	14.512527000	3.934701000
1	13.468330000	16.797686000	5.826097000
1	14.054785000	17.129265000	1.659232000
1	2.634259000	8.538229000	20.299640000
1	6.268537000	6.381541000	20.345304000
1	2.701826000	4.539853000	21.650388000
1	-5.119105000	20.805334000	-5.797260000
1	-5.490877000	21.495128000	-1.650127000
1	-8.762768000	19.958223000	-3.840357000
1	7.143682000	18.719157000	-9.686520000
1	3.189209000	19.020260000	-11.131225000

1	6.026185000	16.702087000	-13.227766000
1	11.568921000	13.350609000	-13.179465000
1	12.772822000	15.283937000	-9.618488000
1	14.934296000	11.952380000	-11.049187000
1	18.205158000	11.297760000	-5.797260000
1	16.273553000	14.501318000	-3.840357000
1	18.746307000	11.864494000	-1.650127000
1	17.206745000	4.000829000	13.179465000
1	19.317103000	4.857288000	9.618488000
1	19.107532000	0.891520000	11.049187000
1	21.368939000	-1.560644000	5.797260000
1	21.689242000	2.166458000	3.840357000
1	22.139856000	-1.420226000	1.650127000
1	5.152567000	-1.166698000	21.650388000
1	8.934369000	0.133129000	20.299640000
1	8.006291000	-3.989729000	20.345304000
1	13.457097000	-5.155707000	16.833157000
1	14.264443000	-1.007037000	16.788807000
1	16.480361000	-3.618831000	14.320701000
1	-10.588869000	16.870672000	-9.618488000
1	-6.752438000	17.896848000	-11.049187000
1	-9.122191000	15.128263000	-13.179465000
1	-2.887485000	8.455951000	-20.299640000
1	1.320377000	8.847329000	-20.345304000
1	-0.482635000	5.260912000	-21.650388000
1	4.854282000	2.084724000	-21.650388000
1	7.149805000	5.359194000	-20.299640000
1	8.822329000	1.478222000	-20.345304000
1	17.746822000	-0.570014000	-13.227766000
1	19.074862000	2.844465000	-11.131225000
1	20.010496000	-1.009507000	-9.686520000
1	-14.022435000	10.892471000	-13.227766000
1	-15.595458000	12.578583000	-9.686520000
1	-17.103823000	8.910702000	-11.131225000
1	-8.822329000	-1.478222000	20.345304000
1	-4.854282000	-2.084724000	21.650388000
1	-7.149805000	-5.359194000	20.299640000
1	-1.320377000	-8.847329000	20.345304000
1	0.482635000	-5.260912000	21.650388000
1	2.887485000	-8.455951000	20.299640000
1	-0.744898000	-14.391661000	16.833157000
1	3.450206000	-13.877483000	16.788807000
1	1.650999000	-16.792035000	14.320701000
1	20.634067000	-8.073661000	1.659232000
1	20.137491000	-7.618372000	5.826097000
1	18.891962000	-11.179957000	3.934701000
1	15.595458000	-12.578583000	9.686520000
1	17.103823000	-8.910702000	11.131225000
1	14.022435000	-10.892471000	13.227766000
1	3.482746000	-3.972481000	-21.650388000
1	7.306308000	-5.143787000	-20.299640000
1	4.132122000	-7.933738000	-20.345304000

1	7.856571000	-12.080938000	-16.833157000
1	10.948256000	-9.199139000	-16.788807000
1	11.205797000	-12.614609000	-14.320701000
1	8.599704000	-17.262284000	-11.131225000
1	4.941954000	-17.054375000	-13.227766000
1	5.223485000	-19.343067000	-9.686520000
1	11.813606000	-17.999913000	-5.826097000
1	11.947732000	-18.660129000	-1.659232000
1	8.712505000	-20.149192000	-3.934701000
1	-1.349757000	-19.872641000	-9.618488000
1	-5.056666000	-18.447837000	-11.049187000
1	-4.641921000	-21.297167000	-3.840357000
1	-8.192306000	-20.617380000	-1.650127000
1	-8.087626000	-19.840803000	-5.797260000
1	-1.512162000	-17.600911000	-13.179465000
1	-4.794834000	-21.422121000	3.934701000
1	-1.022676000	-21.506099000	5.826097000
1	-1.302231000	-22.119062000	1.659232000
1	-3.189209000	-19.020260000	11.131225000
1	-6.026185000	-16.702087000	13.227766000
1	-18.205158000	-11.297760000	5.797260000
1	-16.273553000	-14.501318000	3.840357000
1	-18.746307000	-11.864494000	1.650127000
1	-20.769540000	-5.673128000	5.826097000
1	-21.438889000	-5.596671000	1.659232000
1	-21.855332000	-2.059642000	3.934701000
1	-7.143682000	-18.719157000	9.686520000
1	-19.107532000	-0.891520000	-11.049187000
1	-17.206745000	-4.000829000	-13.179465000
1	-16.480361000	3.618831000	-14.320701000
1	-13.457097000	5.155707000	-16.833157000
1	-14.264443000	1.007037000	-16.788807000
1	-14.692526000	-9.970170000	-13.227766000
1	-16.782205000	-10.945166000	-9.686520000
1	-13.759953000	-13.513144000	-11.131225000
1	-19.317103000	-4.857288000	-9.618488000
1	-16.470708000	-14.512527000	-3.934701000
1	-14.054785000	-17.129265000	-1.659232000
1	-13.468330000	-16.797686000	-5.826097000
1	-9.061841000	-11.205258000	-16.833157000
1	-8.534425000	-14.555475000	-14.320701000
1	-5.365704000	-13.255100000	-16.788807000
1	-6.268537000	-6.381541000	-20.345304000
1	-2.701826000	-4.539853000	-21.650388000
1	-2.634259000	-8.538229000	-20.299640000
1	5.490877000	-21.495128000	1.650127000
1	8.762768000	-19.958223000	3.840357000
1	5.119105000	-20.805334000	5.797260000
1	-12.772822000	-15.283937000	9.618488000
1	-11.568921000	-13.350609000	13.179465000
1	-14.934296000	-11.952380000	11.049187000
1	-21.689242000	-2.166458000	-3.840357000

1	-22.139856000	1.420226000	-1.650127000
1	-21.368939000	1.560644000	-5.797260000
1	-8.934369000	-0.133129000	-20.299640000
1	-8.006291000	3.989729000	-20.345304000
1	-5.152567000	1.166698000	-21.650388000
1	16.272177000	-6.877132000	-13.179465000
1	18.482907000	-7.424679000	-9.618488000
1	15.982340000	-10.509871000	-11.049187000
1	16.370511000	-13.822934000	-5.797260000
1	18.820377000	-10.995915000	-3.840357000
1	17.076732000	-14.162467000	-1.650127000
1	9.122191000	-15.128263000	13.179465000
1	6.752438000	-17.896848000	11.049187000
1	10.588869000	-16.870672000	9.618488000
1	-13.917469000	-3.738828000	16.833157000
1	-15.459988000	-6.759217000	14.320701000
1	-12.132098000	-7.569719000	16.788807000
1	-20.010496000	1.009507000	9.686520000
1	-17.746822000	0.570014000	13.227766000
1	-19.074862000	-2.844465000	11.131225000
1	-20.137491000	7.618372000	-5.826097000
1	-18.891962000	11.179957000	-3.934701000
1	-20.634067000	8.073661000	-1.659232000
1	-18.820377000	10.995915000	3.840357000
1	-17.076732000	14.162467000	1.650127000
1	-16.370511000	13.822934000	5.797260000
1	-15.982340000	10.509871000	11.049187000
1	-16.272177000	6.877132000	13.179465000
1	-18.482907000	7.424679000	9.618488000
1	15.459988000	6.759217000	-14.320701000
1	13.917469000	3.738828000	-16.833157000
1	12.132098000	7.569719000	-16.788807000
1	-3.450206000	13.877483000	-16.788807000
1	0.744898000	14.391661000	-16.833157000
1	-1.650999000	16.792035000	-14.320701000
1	21.438889000	5.596671000	-1.659232000
1	21.855332000	2.059642000	-3.934701000
1	20.769540000	5.673128000	-5.826097000
1	-8.712505000	20.149192000	3.934701000
1	-11.947732000	18.660129000	1.659232000
1	-11.813606000	17.999913000	5.826097000
1	-11.205797000	12.614609000	14.320701000
1	-7.856571000	12.080938000	16.833157000
1	-10.948256000	9.199139000	16.788807000
1	-7.306308000	5.143787000	20.299640000
1	-4.132122000	7.933738000	20.345304000
1	-3.482746000	3.972481000	21.650388000

Cartesian coordinates for COBOC-20-BPDBA

6	-16.934368000	0.035573000	-6.230166000

6	-16.391220000	1.216455000	-6.792427000
6	-16.872846000	-1.143865000	-7.012411000
6	-16.267158000	-1.152208000	-8.274475000
6	-15.761787000	1.206876000	-8.042806000
6	-15.662471000	0.015835000	-8.805437000
6	-5.199176000	16.116534000	-6.230166000
6	-3.908248000	15.964882000	-6.792427000
6	-6.301877000	15.693556000	-7.012411000
6	-6.122643000	15.114935000	-8.274475000
6	-4.824910000	14.900789000	-8.805437000
6	-3.722853000	15.363295000	-8.042806000
5	-3.741552000	11.453918000	-13.470852000
8	-4.700462000	10.565763000	-13.944033000
8	-2.433136000	11.329264000	-13.924560000
5	-2.052646000	10.264800000	-14.733493000
8	-3.031628000	9.390323000	-15.191998000
5	-4.346760000	9.491941000	-14.753441000
6	-5.377614000	8.360901000	-15.058741000
6	-4.943126000	7.101072000	-15.537992000
6	-6.752083000	8.497030000	-14.743986000
6	-7.639090000	7.420866000	-14.865732000
6	-7.187542000	6.141562000	-15.279707000
6	-5.821276000	6.015328000	-15.636680000
5	-10.396707000	1.219815000	-14.733493000
8	-11.526651000	1.186885000	-13.924560000
5	-12.049526000	-0.018972000	-13.470852000
8	-11.501161000	-1.205405000	-13.944033000
5	-10.370595000	-1.200843000	-14.753441000
8	-9.867553000	0.018520000	-15.191998000
6	8.172978000	-2.839391000	-15.537992000
6	9.424763000	-0.743896000	-15.279707000
6	10.542030000	-1.513462000	-14.865732000
6	9.264995000	-3.603228000	-15.058741000
6	10.456979000	-2.905467000	-14.743986000
5	9.128102000	5.124180000	-14.733493000
8	7.993904000	5.785020000	-15.191998000
8	10.022890000	5.814985000	-13.924560000
5	9.095825000	-5.124180000	-14.753441000
8	7.972133000	-5.814985000	-15.191998000
5	7.694124000	-7.097882000	-14.733493000
8	8.627623000	-7.735406000	-13.924560000
5	9.759423000	-7.067185000	-13.470852000
8	10.013154000	-5.785020000	-13.944033000
6	10.614194000	7.755633000	-12.360345000
6	10.130431000	8.863810000	-11.623087000
6	11.851228000	7.196526000	-11.955487000
6	12.550230000	7.694069000	-10.849331000
6	12.029102000	8.759227000	-10.071029000
6	10.813114000	9.347967000	-10.500918000
5	7.684150000	7.067185000	-14.753441000
8	8.596115000	7.735406000	-13.944033000
5	9.737120000	7.097882000	-13.470852000

6	2.213249000	9.218980000	-15.263057000
6	1.808283000	10.240929000	-14.367696000
6	0.464696000	10.618650000	-14.258935000
6	-0.545131000	9.982551000	-15.021338000
6	1.212527000	8.629440000	-16.077236000
6	-0.133245000	8.992203000	-15.946717000
6	2.204925000	-9.193359000	-15.279707000
6	1.818278000	-10.493751000	-14.865732000
6	1.173791000	-8.288163000	-15.636680000
6	-0.174832000	-8.650384000	-15.537992000
6	-0.563833000	-9.924993000	-15.058741000
6	0.468121000	-10.843017000	-14.743986000
6	8.245233000	-1.444842000	-15.636680000
5	-3.705465000	-11.465643000	-13.470852000
8	-2.407646000	-11.310745000	-13.944033000
8	-4.690726000	-10.595729000	-13.924560000
5	-4.372872000	-9.510913000	-14.733493000
5	-2.062620000	-10.234103000	-14.753441000
8	-3.066855000	-9.378878000	-15.191998000
6	-8.062045000	-4.937912000	-15.279707000
6	-7.519790000	-3.677524000	-15.636680000
6	-8.281030000	-2.506840000	-15.537992000
6	-9.613463000	-2.530755000	-15.058741000
6	-10.167664000	-3.795886000	-14.743986000
6	-9.418272000	-4.972033000	-14.865732000
6	-4.855030000	-14.891002000	-8.805437000
6	-3.931013000	-15.827038000	-8.274475000
6	-6.018467000	-14.617405000	-8.042806000
6	-6.222083000	-15.213071000	-6.792427000
6	-5.266839000	-16.094549000	-6.230166000
6	-4.126116000	-16.400504000	-7.012411000
6	12.661898000	-9.218980000	-8.805437000
6	13.837658000	-8.629440000	-8.274475000
6	12.042170000	-10.240929000	-8.042806000
6	12.545761000	-10.618650000	-6.792427000
6	14.322766000	-8.992203000	-7.012411000
6	13.679283000	-9.982551000	-6.230166000
6	-12.766018000	-12.373680000	0.457977000
6	-11.070943000	-14.147106000	0.377620000
6	-11.242020000	-14.313576000	1.775801000
6	-12.887251000	-12.491321000	1.864069000
6	-12.116378000	-13.495037000	2.500524000
5	-6.653001000	-16.589823000	-2.677803000
8	-6.589866000	-16.380748000	-4.050990000
8	-5.545014000	-17.125729000	-2.031740000
5	-13.721969000	-11.453918000	2.677803000
8	-14.574033000	-10.565763000	2.031740000
5	-15.139809000	-9.491941000	2.710079000
8	-14.943922000	-9.390323000	4.082497000
5	-14.096008000	-10.264800000	4.753076000
8	-13.542637000	-11.329264000	4.050990000
6	-12.047717000	-8.733606000	10.071029000

6	-12.231881000	-7.395201000	10.500918000
6	-11.195729000	-9.558380000	10.849331000
6	-10.506534000	-9.047339000	11.955487000
6	-11.560460000	-6.895544000	11.623087000
6	-10.656012000	-7.698076000	12.360345000
6	0.624471000	-17.952586000	0.404559000
6	0.594564000	-17.636130000	1.786228000
6	1.893179000	-18.187820000	-0.184453000
6	3.072904000	-18.039543000	0.554464000
6	3.046387000	-17.680626000	1.924589000
6	1.775802000	-17.514194000	2.527535000
5	4.348914000	-17.331985000	2.710079000
8	4.312803000	-17.114284000	4.082497000
8	5.545014000	-17.125729000	2.031740000
5	6.653001000	-16.589823000	2.677803000
5	5.406499000	-16.578098000	4.753076000
8	6.589866000	-16.380748000	4.050990000
6	5.266839000	-16.094549000	6.230166000
6	6.222083000	-15.213071000	6.792427000
6	4.126116000	-16.400504000	7.012411000
6	3.931013000	-15.827038000	8.274475000
6	4.855030000	-14.891002000	8.805437000
6	6.018467000	-14.617405000	8.042806000
5	3.705465000	-11.465643000	13.470852000
8	2.407646000	-11.310745000	13.944033000
8	4.690726000	-10.595729000	13.924560000
5	4.372872000	-9.510913000	14.733493000
8	3.066855000	-9.378878000	15.191998000
5	2.062620000	-10.234103000	14.753441000
6	-3.628224000	-8.759227000	15.263057000
6	-4.091303000	-7.694069000	16.077236000
6	-4.556535000	-9.347967000	14.367696000
6	-5.865539000	-8.863810000	14.258935000
6	-6.308617000	-7.755633000	15.021338000
6	-5.393282000	-7.196526000	15.946717000
5	-7.694124000	-7.097882000	14.733493000
8	-8.627623000	-7.735406000	13.924560000
5	-9.759423000	-7.067185000	13.470852000
8	-10.013154000	-5.785020000	13.944033000
5	-9.095825000	-5.124180000	14.753441000
8	-7.972133000	-5.814985000	15.191998000
5	-5.406499000	-16.578098000	-4.753076000
8	-4.312803000	-17.114284000	-4.082497000
5	-4.348914000	-17.331985000	-2.710079000
6	7.897572000	-16.116534000	1.864069000
6	9.090376000	-15.693556000	2.500524000
6	10.139045000	-15.114935000	1.775801000
6	10.033588000	-14.900789000	0.377620000
6	8.858615000	-15.363295000	-0.267031000
6	7.823153000	-15.964882000	0.457977000
6	-11.873899000	-13.172563000	-0.267031000
6	7.832393000	3.819825000	16.077236000

6	9.662425000	2.566327000	15.021338000
6	9.955338000	3.723296000	14.258935000
6	8.083840000	4.953747000	15.263057000
6	9.180912000	4.884400000	14.367696000
5	10.396707000	1.219815000	14.733493000
8	9.867553000	0.018520000	15.191998000
8	11.526651000	1.186885000	13.924560000
5	4.346760000	9.491941000	14.753441000
8	3.031628000	9.390323000	15.191998000
5	2.052646000	10.264800000	14.733493000
8	2.433136000	11.329264000	13.924560000
5	3.741552000	11.453918000	13.470852000
8	4.700462000	10.565763000	13.944033000
6	15.662471000	0.015835000	8.805437000
6	15.761787000	1.206876000	8.042806000
6	16.267158000	-1.152208000	8.274475000
6	16.872846000	-1.143865000	7.012411000
6	16.934368000	0.035573000	6.230166000
6	16.391220000	1.216455000	6.792427000
5	17.437408000	0.018972000	4.753076000
8	17.615398000	1.205405000	4.050990000
8	17.609380000	-1.186885000	4.082497000
5	17.827586000	-1.219815000	2.710079000
5	17.833750000	1.200843000	2.677803000
8	18.001040000	-0.018520000	2.031740000
5	10.370595000	-1.200843000	14.753441000
8	11.501161000	-1.205405000	13.944033000
5	12.049526000	-0.018972000	13.470852000
6	17.756661000	-2.566327000	1.924589000
6	17.205742000	-3.723296000	2.527535000
6	16.956687000	-4.884400000	1.786228000
6	17.266896000	-4.953747000	0.404559000
6	17.882669000	-3.819825000	-0.184453000
6	18.106204000	-2.652020000	0.554464000
6	9.613463000	-2.530755000	15.058741000
6	10.167664000	-3.795886000	14.743986000
6	9.418272000	-4.972033000	14.865732000
6	8.062045000	-4.937912000	15.279707000
6	8.281030000	-2.506840000	15.537992000
6	7.519790000	-3.677524000	15.636680000
6	-3.619899000	8.733606000	15.279707000
6	-3.922043000	7.395201000	15.636680000
6	-4.697054000	9.558380000	14.865732000
6	-5.994648000	9.047339000	14.743986000
6	-6.289915000	7.698076000	15.058741000
6	-5.226011000	6.895544000	15.537992000
6	8.593269000	2.652020000	15.946717000
5	-9.737120000	7.097882000	13.470852000
8	-8.596115000	7.735406000	13.944033000
8	-10.022890000	5.814985000	13.924560000
5	-9.128102000	5.124180000	14.733493000
5	-7.684150000	7.067185000	14.753441000

8	-7.993904000	5.785020000	15.191998000
6	-9.424763000	-0.743896000	15.279707000
6	-8.245233000	-1.444842000	15.636680000
6	-8.172978000	-2.839391000	15.537992000
6	-9.264995000	-3.603228000	15.058741000
6	-10.456979000	-2.905467000	14.743986000
6	-10.542030000	-1.513462000	14.865732000
6	-15.873890000	-8.360901000	1.924589000
6	-16.207045000	-8.497030000	0.554464000
6	-16.108236000	-7.101072000	2.527535000
6	-16.589226000	-6.015328000	1.786228000
6	-16.880952000	-6.141562000	0.404559000
6	-16.712620000	-7.420866000	-0.184453000
5	-17.827586000	-1.219815000	-2.710079000
8	-17.609380000	-1.186885000	-4.082497000
5	-17.437408000	0.018972000	-4.753076000
8	-17.615398000	1.205405000	-4.050990000
5	-17.833750000	1.200843000	-2.677803000
8	-18.001040000	-0.018520000	-2.031740000
6	-10.614194000	7.755633000	12.360345000
6	-10.130431000	8.863810000	11.623087000
6	-11.851228000	7.196526000	11.955487000
6	-12.550230000	7.694069000	10.849331000
6	-12.029102000	8.759227000	10.071029000
6	-10.813114000	9.347967000	10.500918000
5	-13.705831000	11.465643000	2.710079000
8	-13.548654000	11.310745000	4.082497000
8	-14.552262000	10.595729000	2.031740000
5	-15.133645000	9.510913000	2.677803000
5	-14.118311000	10.234103000	4.753076000
8	-14.959675000	9.378878000	4.050990000
6	-17.272041000	4.937912000	-0.377620000
6	-17.508294000	4.972033000	-1.775801000
6	-17.734540000	3.795886000	-2.500524000
6	-17.768219000	2.530755000	-1.864069000
6	-17.600992000	2.506840000	-0.457977000
6	-17.348824000	3.677524000	0.267031000
5	13.721969000	-11.453918000	-2.677803000
8	14.574033000	-10.565763000	-2.031740000
5	15.139809000	-9.491941000	-2.710079000
8	13.542637000	-11.329264000	-4.050990000
8	14.943922000	-9.390323000	-4.082497000
5	14.096008000	-10.264800000	-4.753076000
6	4.096079000	12.491321000	12.360345000
6	5.299509000	12.373680000	11.623087000
6	3.182072000	13.495037000	11.955487000
6	3.439260000	14.313576000	10.849331000
6	5.549009000	13.172563000	10.500918000
6	4.613323000	14.147106000	10.071029000
6	17.768219000	2.530755000	1.864069000
6	17.734540000	3.795886000	2.500524000
6	17.600992000	2.506840000	0.457977000

6	17.348824000	3.677524000	-0.267031000
6	17.272041000	4.937912000	0.377620000
6	17.508294000	4.972033000	1.775801000
5	15.133645000	9.510913000	-2.677803000
8	14.552262000	10.595729000	-2.031740000
8	14.959675000	9.378878000	-4.050990000
5	14.118311000	10.234103000	-4.753076000
8	13.548654000	11.310745000	-4.082497000
5	13.705831000	11.465643000	-2.710079000
6	12.856991000	12.513306000	-1.924589000
6	13.089408000	12.788089000	-0.554464000
6	11.731239000	13.125491000	-2.527535000
6	10.847269000	13.918453000	-1.786228000
6	11.057473000	14.156892000	-0.404559000
6	12.222147000	13.601473000	0.184453000
5	6.669140000	16.578098000	2.710079000
8	6.570393000	16.380748000	4.082497000
5	5.370412000	16.589823000	4.753076000
8	4.297049000	17.125729000	4.050990000
5	4.368862000	17.331985000	2.677803000
8	5.580240000	17.114284000	2.031740000
6	-12.856991000	12.513306000	1.924589000
6	-13.089408000	12.788089000	0.554464000
6	-11.731239000	13.125491000	2.527535000
6	-10.847269000	13.918453000	1.786228000
6	-12.222147000	13.601473000	-0.184453000
6	-11.057473000	14.156892000	0.404559000
6	-0.641121000	17.952586000	-0.377620000
6	-1.863548000	17.636130000	0.267031000
6	-0.681676000	18.187820000	-1.775801000
6	-1.870172000	18.039543000	-2.500524000
6	-3.083791000	17.680626000	-1.864069000
6	-3.054859000	17.514194000	-0.457977000
5	-4.368862000	17.331985000	-2.677803000
8	-5.580240000	17.114284000	-2.031740000
5	-6.669140000	16.578098000	-2.710079000
8	-6.570393000	16.380748000	-4.082497000
5	-5.370412000	16.589823000	-4.753076000
8	-4.297049000	17.125729000	-4.050990000
6	-17.266896000	-4.953747000	-0.404559000
6	-17.882669000	-3.819825000	0.184453000
6	-16.956687000	-4.884400000	-1.786228000
6	-17.205742000	-3.723296000	-2.527535000
6	-17.756661000	-2.566327000	-1.924589000
6	-18.106204000	-2.652020000	-0.554464000
6	-14.880293000	-0.015835000	-10.071029000
6	-14.242590000	-1.206876000	-10.500918000
6	-13.405707000	-1.216455000	-11.623087000
6	-13.145710000	-0.035573000	-12.360345000
6	-13.817857000	1.143865000	-11.955487000
6	-14.675810000	1.152208000	-10.849331000
6	-7.209337000	-6.157396000	-15.263057000

6	-6.053212000	-6.268658000	-16.077236000
6	-5.177687000	-7.353165000	-15.946717000
6	-5.426576000	-8.396473000	-15.021338000
6	-6.617433000	-8.317527000	-14.258935000
6	-7.482398000	-7.222203000	-14.367696000
6	-4.583204000	-14.156892000	-10.071029000
6	-5.630890000	-13.601473000	-10.849331000
6	-3.253395000	-13.918453000	-10.500918000
6	-10.033588000	-14.900789000	-0.377620000
6	-10.139045000	-15.114935000	-1.775801000
6	-8.858615000	-15.363295000	0.267031000
6	-7.823153000	-15.964882000	-0.457977000
6	-7.897572000	-16.116534000	-1.864069000
6	-9.090376000	-15.693556000	-2.500524000
6	-0.624471000	-17.952586000	-0.404559000
6	-0.594564000	-17.636130000	-1.786228000
6	-1.775802000	-17.514194000	-2.527535000
6	-3.046387000	-17.680626000	-1.924589000
6	-3.072904000	-18.039543000	-0.554464000
6	-1.893179000	-18.187820000	0.184453000
6	4.583204000	-14.156892000	10.071029000
6	5.630890000	-13.601473000	10.849331000
6	5.357833000	-12.788089000	11.955487000
6	4.028416000	-12.513306000	12.360345000
6	2.985674000	-13.125491000	11.623087000
6	3.253395000	-13.918453000	10.500918000
6	-2.204925000	-9.193359000	15.279707000
6	-1.818278000	-10.493751000	14.865732000
6	-1.173791000	-8.288163000	15.636680000
6	0.174832000	-8.650384000	15.537992000
6	0.563833000	-9.924993000	15.058741000
6	-0.468121000	-10.843017000	14.743986000
6	-12.661898000	-9.218980000	8.805437000
6	-13.837658000	-8.629440000	8.274475000
6	-14.322766000	-8.992203000	7.012411000
6	-12.042170000	-10.240929000	8.042806000
6	-2.213249000	9.218980000	15.263057000
6	-1.212527000	8.629440000	16.077236000
6	0.133245000	8.992203000	15.946717000
6	0.545131000	9.982551000	15.021338000
6	-0.464696000	10.618650000	14.258935000
6	-1.808283000	10.240929000	14.367696000
6	5.199176000	16.116534000	6.230166000
6	6.301877000	15.693556000	7.012411000
6	6.122643000	15.114935000	8.274475000
6	4.824910000	14.900789000	8.805437000
6	3.722853000	15.363295000	8.042806000
6	3.908248000	15.964882000	6.792427000
6	0.641121000	17.952586000	0.377620000
6	0.681676000	18.187820000	1.775801000
6	1.870172000	18.039543000	2.500524000
6	3.083791000	17.680626000	1.864069000

6	3.054859000	17.514194000	0.457977000
6	1.863548000	17.636130000	-0.267031000
6	-4.096079000	12.491321000	-12.360345000
6	-5.299509000	12.373680000	-11.623087000
6	-5.549009000	13.172563000	-10.500918000
6	-4.613323000	14.147106000	-10.071029000
6	-3.439260000	14.313576000	-10.849331000
6	-3.182072000	13.495037000	-11.955487000
6	-9.662425000	2.566327000	-15.021338000
6	-8.593269000	2.652020000	-15.946717000
6	-7.832393000	3.819825000	-16.077236000
6	-8.083840000	4.953747000	-15.263057000
6	-9.180912000	4.884400000	-14.367696000
6	-9.955338000	3.723296000	-14.258935000
6	-10.047058000	14.891002000	-0.404559000
6	-9.158918000	15.827038000	0.184453000
6	-8.117346000	16.400504000	-0.554464000
6	-7.927832000	16.094549000	-1.924589000
6	-8.857931000	15.213071000	-2.527535000
6	-9.885245000	14.617405000	-1.786228000
6	11.070943000	-14.147106000	-0.377620000
6	11.873899000	-13.172563000	0.267031000
6	12.766018000	-12.373680000	-0.457977000
6	12.887251000	-12.491321000	-1.864069000
6	12.116378000	-13.495037000	-2.500524000
6	11.242020000	-14.313576000	-1.775801000
6	-12.680513000	9.193359000	8.805437000
6	-12.483156000	10.493751000	8.274475000
6	-13.460937000	8.288163000	8.042806000
6	-13.975790000	8.650384000	6.792427000
6	-13.721101000	9.924993000	6.230166000
6	-12.978072000	10.843017000	7.012411000
6	12.047717000	-8.733606000	-10.071029000
6	12.231881000	-7.395201000	-10.500918000
6	11.560460000	-6.895544000	-11.623087000
6	10.656012000	-7.698076000	-12.360345000
6	10.506534000	-9.047339000	-11.955487000
6	11.195729000	-9.558380000	-10.849331000
6	-9.451703000	0.743896000	15.263057000
6	-8.581776000	1.513462000	16.077236000
6	-8.510919000	2.905467000	15.946717000
6	-9.325515000	3.603228000	15.021338000
6	-10.242536000	2.839391000	14.258935000
6	-10.298492000	1.444842000	14.367696000
6	7.209337000	-6.157396000	15.263057000
6	7.482398000	-7.222203000	14.367696000
6	6.053212000	-6.268658000	16.077236000
6	5.177687000	-7.353165000	15.946717000
6	5.426576000	-8.396473000	15.021338000
6	6.617433000	-8.317527000	14.258935000
6	14.880293000	-0.015835000	10.071029000
6	14.242590000	-1.206876000	10.500918000

6	13.405707000	-1.216455000	11.623087000
6	13.145710000	-0.035573000	12.360345000
6	13.817857000	1.143865000	11.955487000
6	14.675810000	1.152208000	10.849331000
6	7.187542000	6.141562000	15.279707000
6	5.821276000	6.015328000	15.636680000
6	4.943126000	7.101072000	15.537992000
6	5.377614000	8.360901000	15.058741000
6	6.752083000	8.497030000	14.743986000
6	7.639090000	7.420866000	14.865732000
6	10.047058000	14.891002000	0.404559000
6	9.885245000	14.617405000	1.786228000
6	8.857931000	15.213071000	2.527535000
6	7.927832000	16.094549000	1.924589000
6	8.117346000	16.400504000	0.554464000
6	9.158918000	15.827038000	-0.184453000
6	12.680513000	9.193359000	-8.805437000
6	13.460937000	8.288163000	-8.042806000
6	13.975790000	8.650384000	-6.792427000
6	13.721101000	9.924993000	-6.230166000
6	12.978072000	10.843017000	-7.012411000
6	12.483156000	10.493751000	-8.274475000
6	9.451703000	0.743896000	-15.263057000
6	8.581776000	1.513462000	-16.077236000
6	8.510919000	2.905467000	-15.946717000
6	9.325515000	3.603228000	-15.021338000
6	10.242536000	2.839391000	-14.258935000
6	10.298492000	1.444842000	-14.367696000
6	3.628224000	-8.759227000	-15.263057000
6	4.091303000	-7.694069000	-16.077236000
6	5.393282000	-7.196526000	-15.946717000
6	6.308617000	-7.755633000	-15.021338000
6	5.865539000	-8.863810000	-14.258935000
6	4.556535000	-9.347967000	-14.367696000
6	-16.875807000	6.157396000	0.377620000
6	-17.086995000	6.268658000	1.775801000
6	-16.578709000	7.353165000	2.500524000
6	-15.862331000	8.396473000	1.864069000
6	-15.712985000	8.317527000	0.457977000
6	-16.197088000	7.222203000	-0.267031000
6	16.880952000	-6.141562000	-0.404559000
6	16.712620000	-7.420866000	0.184453000
6	16.207045000	-8.497030000	-0.554464000
6	15.873890000	-8.360901000	-1.924589000
6	16.108236000	-7.101072000	-2.527535000
6	16.589226000	-6.015328000	-1.786228000
6	16.875807000	6.157396000	-0.377620000
6	17.086995000	6.268658000	-1.775801000
6	16.197088000	7.222203000	0.267031000
6	15.712985000	8.317527000	-0.457977000
6	15.862331000	8.396473000	-1.864069000
6	16.578709000	7.353165000	-2.500524000

6	3.619899000	8.733606000	-15.279707000
6	3.922043000	7.395201000	-15.636680000
6	5.226011000	6.895544000	-15.537992000
6	6.289915000	7.698076000	-15.058741000
6	5.994648000	9.047339000	-14.743986000
6	4.697054000	9.558380000	-14.865732000
6	-5.357833000	-12.788089000	-11.955487000
6	-4.028416000	-12.513306000	-12.360345000
6	-2.985674000	-13.125491000	-11.623087000
6	-12.545761000	-10.618650000	6.792427000
6	-13.679283000	-9.982551000	6.230166000
1	-16.424306000	2.153375000	-6.211872000
1	-17.299775000	-2.079005000	-6.613011000
1	-16.255230000	-2.086725000	-8.858029000
1	-15.289339000	2.131647000	-8.410415000
1	-3.027409000	16.285872000	-6.211872000
1	-7.323176000	15.810616000	-6.613011000
1	-7.007736000	14.814809000	-8.858029000
1	-2.697348000	15.199741000	-8.410415000
1	-3.880000000	6.960597000	-15.794869000
1	-7.129165000	9.468386000	-14.382665000
1	-8.703850000	7.573124000	-14.626770000
1	-5.425922000	5.034544000	-15.945395000
1	7.230322000	-3.350635000	-15.794869000
1	11.492933000	-1.010791000	-14.626770000
1	11.332993000	-3.469667000	-14.382665000
1	9.171601000	9.325275000	-11.912312000
1	12.266346000	6.342230000	-12.515961000
1	13.517474000	7.241685000	-10.577689000
1	10.364618000	10.165196000	-9.913948000
1	2.551106000	10.711387000	-13.704048000
1	0.179746000	11.396081000	-13.530677000
1	1.493136000	7.866389000	-16.820882000
1	-0.890759000	8.486602000	-16.568661000
1	2.590192000	-11.242781000	-14.626770000
1	1.430433000	-7.262309000	-15.945395000
1	-0.952351000	-7.911848000	-15.794869000
1	0.202239000	-11.850503000	-14.382665000
1	7.348894000	-0.883755000	-15.945395000
1	-6.464838000	-3.604599000	-15.945395000
1	-7.818907000	-1.539156000	-15.794869000
1	-11.208003000	-3.854347000	-14.382665000
1	-9.892106000	-5.937630000	-14.626770000
1	-3.038549000	-16.104476000	-8.858029000
1	-6.751983000	-13.882310000	-8.410415000
1	-7.123371000	-14.955014000	-6.211872000
1	-3.368673000	-17.095512000	-6.613011000
1	14.377304000	-7.866389000	-8.858029000
1	11.116384000	-10.711387000	-8.410415000
1	12.021821000	-11.396081000	-6.211872000
1	15.217821000	-8.486602000	-6.613011000
1	-13.353488000	-11.604379000	-0.070237000

1	-10.672977000	-15.093685000	2.306479000
1	-12.196960000	-13.625845000	3.592619000
1	-12.870519000	-6.716119000	9.913948000
1	-11.064381000	-10.618077000	10.577689000
1	-9.822329000	-9.706131000	12.515961000
1	-11.703044000	-5.841042000	11.912312000
1	-0.368907000	-17.427505000	2.277905000
1	1.956887000	-18.484466000	-1.243670000
1	4.044472000	-18.192733000	0.055604000
1	1.718124000	-17.237123000	3.593303000
1	7.123371000	-14.955014000	6.211872000
1	3.368673000	-17.095512000	6.613011000
1	3.038549000	-16.104476000	8.858029000
1	6.751983000	-13.882310000	8.410415000
1	-3.415775000	-7.241685000	16.820882000
1	-4.232107000	-10.165196000	13.704048000
1	-6.553031000	-9.325275000	13.530677000
1	-5.708939000	-6.342230000	16.568661000
1	9.189881000	-15.810616000	3.592619000
1	11.056817000	-14.814809000	2.306479000
1	8.728795000	-15.199741000	-1.348670000
1	6.909965000	-16.285872000	-0.070237000
1	-11.758466000	-12.998555000	-1.348670000
1	7.019976000	3.850905000	16.820882000
1	10.782773000	3.692531000	13.530677000
1	9.398799000	5.736247000	13.704048000
1	15.289339000	2.131647000	8.410415000
1	16.255230000	-2.086725000	8.858029000
1	17.299775000	-2.079005000	6.613011000
1	16.424306000	2.153375000	6.211872000
1	16.924408000	-3.692531000	3.593303000
1	16.460544000	-5.736247000	2.277905000
1	18.552128000	-1.775342000	0.055604000
1	11.208003000	-3.854347000	14.382665000
1	9.892106000	-5.937630000	14.626770000
1	7.818907000	-1.539156000	15.794869000
1	6.464838000	-3.604599000	15.945395000
1	-3.111434000	6.716119000	15.945395000
1	-4.512831000	10.618077000	14.626770000
1	-6.801937000	9.706131000	14.382665000
1	-5.420935000	5.841042000	15.794869000
1	8.346498000	1.775342000	16.568661000
1	-7.348894000	-0.883755000	15.945395000
1	-7.230322000	-3.350635000	15.794869000
1	-11.332993000	-3.469667000	14.382665000
1	-11.492933000	-1.010791000	14.626770000
1	-16.052507000	-9.468386000	0.055604000
1	-15.862549000	-6.960597000	3.593303000
1	-16.688541000	-5.034544000	2.277905000
1	-16.975061000	-7.573124000	-1.243670000
1	-9.171601000	9.325275000	11.912312000
1	-12.266346000	6.342230000	12.515961000

1	-13.517474000	7.241685000	10.577689000
1	-10.364618000	10.165196000	9.913948000
1	-17.506465000	5.937630000	-2.306479000
1	-17.876618000	3.854347000	-3.592619000
1	-17.624082000	1.539156000	0.070237000
1	-17.153158000	3.604599000	1.348670000
1	6.034683000	11.604379000	11.912312000
1	2.241310000	13.625845000	12.515961000
1	2.710123000	15.093685000	10.577689000
1	6.464833000	12.998555000	9.913948000
1	17.876618000	3.854347000	3.592619000
1	17.624082000	1.539156000	-0.070237000
1	17.153158000	3.604599000	-1.348670000
1	17.506465000	5.937630000	2.306479000
1	13.965467000	12.340949000	-0.055604000
1	11.521718000	12.935238000	-3.593303000
1	9.945179000	14.315986000	-2.277905000
1	12.448051000	13.804018000	1.243670000
1	18.184483000	-3.850905000	-1.243670000
1	-13.965467000	12.340949000	0.055604000
1	-11.521718000	12.935238000	3.593303000
1	-9.945179000	14.315986000	2.277905000
1	-12.448051000	13.804018000	-1.243670000
1	-1.872440000	17.427505000	1.348670000
1	0.237226000	18.484466000	-2.306479000
1	-1.858477000	18.192733000	-3.592619000
1	-3.982316000	17.237123000	0.070237000
1	-18.184483000	-3.850905000	1.243670000
1	-16.460544000	-5.736247000	-2.277905000
1	-16.924408000	-3.692531000	-3.593303000
1	-18.552128000	-1.775342000	-0.055604000
1	-14.360104000	-2.131647000	-9.913948000
1	-12.901240000	-2.153375000	-11.912312000
1	-13.651552000	2.079005000	-12.515961000
1	-15.192422000	2.086725000	-10.577689000
1	-5.831720000	-5.486399000	-16.820882000
1	-4.267660000	-7.389380000	-16.568661000
1	-6.843866000	-9.113972000	-13.530677000
1	-8.359883000	-7.166191000	-13.704048000
1	-6.679310000	-13.804018000	-10.577689000
1	-2.410199000	-14.315986000	-9.913948000
1	-11.056817000	-14.814809000	-2.306479000
1	-8.728795000	-15.199741000	1.348670000
1	-6.909965000	-16.285872000	0.070237000
1	-9.189881000	-15.810616000	-3.592619000
1	0.368907000	-17.427505000	-2.277905000
1	-1.718124000	-17.237123000	-3.593303000
1	-4.044472000	-18.192733000	-0.055604000
1	-1.956887000	-18.484466000	1.243670000
1	6.679310000	-13.804018000	10.577689000
1	6.195813000	-12.340949000	12.515961000
1	1.938721000	-12.935238000	11.912312000

1	2.410199000	-14.315986000	9.913948000
1	-2.590192000	-11.242781000	14.626770000
1	-1.430433000	-7.262309000	15.945395000
1	0.952351000	-7.911848000	15.794869000
1	-0.202239000	-11.850503000	14.382665000
1	-14.377304000	-7.866389000	8.858029000
1	-11.116384000	-10.711387000	8.410415000
1	-1.493136000	7.866389000	16.820882000
1	0.890759000	8.486602000	16.568661000
1	-0.179746000	11.396081000	13.530677000
1	-2.551106000	10.711387000	13.704048000
1	7.323176000	15.810616000	6.613011000
1	7.007736000	14.814809000	8.858029000
1	2.697348000	15.199741000	8.410415000
1	3.027409000	16.285872000	6.211872000
1	-0.237226000	18.484466000	2.306479000
1	1.858477000	18.192733000	3.592619000
1	3.982316000	17.237123000	-0.070237000
1	1.872440000	17.427505000	-1.348670000
1	-6.034683000	11.604379000	-11.912312000
1	-6.464833000	12.998555000	-9.913948000
1	-2.710123000	15.093685000	-10.577689000
1	-2.241310000	13.625845000	-12.515961000
1	-8.346498000	1.775342000	-16.568661000
1	-7.019976000	3.850905000	-16.820882000
1	-9.398799000	5.736247000	-13.704048000
1	-10.782773000	3.692531000	-13.530677000
1	-9.281742000	16.104476000	1.243670000
1	-7.421373000	17.095512000	-0.055604000
1	-8.741735000	14.955014000	-3.593303000
1	-10.542082000	13.882310000	-2.277905000
1	11.758466000	-12.998555000	1.348670000
1	13.353488000	-11.604379000	0.070237000
1	12.196960000	-13.625845000	-3.592619000
1	10.672977000	-15.093685000	-2.306479000
1	-11.924211000	11.242781000	8.858029000
1	-13.622286000	7.262309000	8.410415000
1	-14.553264000	7.911848000	6.211872000
1	-12.773803000	11.850503000	6.613011000
1	12.870519000	-6.716119000	-9.913948000
1	11.703044000	-5.841042000	-11.912312000
1	9.822329000	-9.706131000	-12.515961000
1	11.064381000	-10.618077000	-10.577689000
1	-7.942785000	1.010791000	16.820882000
1	-7.795978000	3.469667000	16.568661000
1	-10.893862000	3.350635000	13.530677000
1	-10.975469000	0.883755000	13.704048000
1	8.359883000	-7.166191000	13.704048000
1	5.831720000	-5.486399000	16.820882000
1	4.267660000	-7.389380000	16.568661000
1	6.843866000	-9.113972000	13.530677000
1	14.360104000	-2.131647000	9.913948000

1	12.901240000	-2.153375000	11.912312000
1	13.651552000	2.079005000	12.515961000
1	15.192422000	2.086725000	10.577689000
1	5.425922000	5.034544000	15.945395000
1	3.880000000	6.960597000	15.794869000
1	7.129165000	9.468386000	14.382665000
1	8.703850000	7.573124000	14.626770000
1	10.542082000	13.882310000	2.277905000
1	8.741735000	14.955014000	3.593303000
1	7.421373000	17.095512000	0.055604000
1	9.281742000	16.104476000	-1.243670000
1	13.622286000	7.262309000	-8.410415000
1	14.553264000	7.911848000	-6.211872000
1	12.773803000	11.850503000	-6.613011000
1	11.924211000	11.242781000	-8.858029000
1	7.942785000	1.010791000	-16.820882000
1	7.795978000	3.469667000	-16.568661000
1	10.893862000	3.350635000	-13.530677000
1	10.975469000	0.883755000	-13.704048000
1	3.415775000	-7.241685000	-16.820882000
1	5.708939000	-6.342230000	-16.568661000
1	6.553031000	-9.325275000	-13.530677000
1	4.232107000	-10.165196000	-13.704048000
1	-17.653079000	5.486399000	2.306479000
1	-16.728017000	7.389380000	3.592619000
1	-15.162875000	9.113972000	-0.070237000
1	-15.995927000	7.166191000	-1.348670000
1	16.975061000	-7.573124000	1.243670000
1	16.052507000	-9.468386000	-0.055604000
1	15.862549000	-6.960597000	-3.593303000
1	16.688541000	-5.034544000	-2.277905000
1	17.653079000	5.486399000	-2.306479000
1	15.995927000	7.166191000	1.348670000
1	15.162875000	9.113972000	0.070237000
1	16.728017000	7.389380000	-3.592619000
1	3.111434000	6.716119000	-15.945395000
1	5.420935000	5.841042000	-15.794869000
1	6.801937000	9.706131000	-14.382665000
1	4.512831000	10.618077000	-14.626770000
1	-6.195813000	-12.340949000	-12.515961000
1	-1.938721000	-12.935238000	-11.912312000
1	-15.217821000	-8.486602000	6.613011000
1	-12.021821000	-11.396081000	6.211872000

Cartesian coordinates for COBOC-20-TPDBA

6	10.801549000	20.968524000	3.678568000
6	11.868425000	20.449019000	2.934533000
6	5.081563000	15.931932000	17.210889000
6	4.070589000	16.772392000	16.682755000
6	6.366251000	16.007188000	16.618739000

6	6.619327000	16.848788000	15.528446000
6	4.318080000	17.616192000	15.593790000
6	5.593116000	17.655192000	14.975750000
6	-17.607913000	15.968917000	3.057616000
6	-17.777132000	16.116636000	1.658703000
6	-16.604389000	16.752514000	3.678568000
6	-15.780627000	17.606637000	2.934533000
6	-15.922051000	17.714086000	1.528388000
6	-16.958272000	16.969937000	0.910557000
5	-8.457765000	22.180347000	-3.834172000
8	-7.334283000	22.668983000	-3.177312000
8	-8.401992000	22.004665000	-5.212156000
5	-7.216376000	22.191836000	-5.913371000
8	-6.112163000	22.694430000	-5.234862000
5	-6.144199000	22.896452000	-3.859443000
6	-4.845515000	23.280648000	-3.085760000
6	-3.610791000	23.443672000	-3.761111000
6	-4.832784000	23.377401000	-1.672524000
6	-3.641829000	23.583991000	-0.965676000
6	-2.402430000	23.700970000	-1.643170000
6	-2.417461000	23.651886000	-3.059781000
5	6.183123000	22.896001000	3.827087000
8	6.095680000	22.636925000	5.189821000
5	7.160173000	22.073142000	5.884649000
8	8.356109000	21.849981000	5.211004000
5	8.474966000	22.113912000	3.851200000
8	7.399824000	22.692867000	3.186582000
1	3.063747000	16.749575000	17.131874000
1	7.175003000	15.364805000	17.005107000
1	7.617020000	16.843551000	15.059628000
1	3.510783000	18.263485000	15.213910000
1	-18.561133000	15.533472000	1.147723000
1	-16.446246000	16.660759000	4.766058000
1	-14.974076000	18.160395000	3.442538000
1	-17.122605000	17.066100000	-0.175169000
1	-3.589189000	23.390586000	-4.862335000
1	-5.777115000	23.253995000	-1.116627000
1	-3.661459000	23.600736000	0.136334000
1	-1.475747000	23.782910000	-3.617565000
6	4.101655000	10.497314000	-20.428577000
6	2.436075000	12.299958000	-20.395395000
6	3.506318000	13.227360000	-20.406187000
6	5.172731000	11.423221000	-20.404878000
6	4.840331000	12.799970000	-20.411116000
5	-3.235502000	13.700164000	-19.568354000
8	-3.595949000	14.736260000	-18.714193000
8	-4.228040000	12.850936000	-20.044376000
5	10.603272000	9.267757000	-19.588245000
8	11.499370000	9.911387000	-18.742958000
5	12.631449000	9.260954000	-18.264341000
8	12.903807000	7.973706000	-18.714193000
5	12.029805000	7.310729000	-19.568354000

8	10.915430000	7.992262000	-20.044376000
6	-9.265667000	8.449530000	-20.404878000
6	-10.677750000	8.558837000	-20.411116000
6	-8.716058000	7.144754000	-20.428577000
6	-9.533608000	6.009663000	-20.424212000
6	-10.945167000	6.117741000	-20.395395000
6	-11.496455000	7.422186000	-20.406187000
5	-4.904359000	14.875014000	-18.264341000
8	-5.872788000	13.999337000	-18.742958000
5	-5.537570000	12.948205000	-19.588245000
6	-6.173758000	18.480834000	-13.767341000
6	-7.102411000	18.025583000	-12.800179000
6	-7.326440000	18.734574000	-11.615177000
6	-6.633865000	19.937003000	-11.333197000
6	-5.505675000	19.701020000	-13.501606000
6	-5.729838000	20.410839000	-12.314990000
6	15.668522000	11.582485000	-13.767341000
6	14.948583000	12.325006000	-12.800179000
6	17.035436000	11.324158000	-13.501606000
6	17.641244000	11.756696000	-12.314990000
6	16.911240000	12.470054000	-11.333197000
6	15.553644000	12.757161000	-11.615177000
1	4.315872000	9.417095000	-20.390819000
1	3.289701000	14.307892000	-20.430115000
1	5.644162000	13.553694000	-20.438928000
1	-11.146187000	9.556239000	-20.438928000
1	-7.622512000	7.014680000	-20.390819000
1	-9.063860000	5.013718000	-20.383464000
1	-12.591040000	7.550073000	-20.430115000
1	-7.618854000	17.064196000	-12.952635000
1	-8.013583000	18.314211000	-10.863313000
1	-4.808861000	20.113673000	-14.249342000
1	-5.204233000	21.366379000	-12.154720000
1	13.874659000	12.519087000	-12.952635000
1	17.643219000	10.788966000	-14.249342000
1	18.712438000	11.552094000	-12.154720000
1	14.941516000	13.280773000	-10.863313000
6	2.769482000	10.924088000	-20.424212000
5	19.877158000	12.918874000	-3.859443000
8	19.694923000	12.825977000	-5.234862000
8	19.293066000	13.980419000	-3.177312000
5	18.481171000	14.897917000	-3.834172000
5	18.875707000	13.720836000	-5.913371000
8	18.331322000	14.790585000	-5.212156000
6	14.967291000	18.537942000	-0.799872000
6	13.662772000	18.754932000	-1.305938000
6	12.700255000	19.448867000	-0.565413000
6	12.989809000	19.963330000	0.721589000
6	14.303815000	19.775194000	1.215112000
6	15.267871000	19.080483000	0.473298000
1	13.377284000	18.320545000	-2.277525000
1	11.680575000	19.544035000	-0.972094000

1	14.583386000	20.198706000	2.193658000
1	16.284917000	18.972784000	0.884435000
6	23.349143000	4.906800000	1.449479000
6	23.212530000	4.929780000	2.860004000
6	23.708446000	3.678019000	0.841040000
6	23.867702000	2.515637000	1.604291000
6	23.682292000	2.522929000	3.008931000
6	23.373692000	3.763777000	3.618664000
1	22.918345000	5.866461000	3.361283000
1	23.865547000	3.637693000	-0.249279000
1	24.125537000	1.568948000	1.101021000
1	23.221821000	3.803707000	4.710164000
6	12.450744000	1.484051000	-20.395395000
6	11.245242000	0.741795000	-20.424212000
6	13.663479000	0.752772000	-20.406187000
6	13.669239000	-0.648020000	-20.411116000
6	11.251020000	-0.657057000	-20.428577000
6	12.462591000	-1.389590000	-20.404878000
1	10.279806000	1.271420000	-20.383464000
1	14.624187000	1.292690000	-20.430115000
1	14.634471000	-1.179596000	-20.438928000
1	10.289867000	-1.194595000	-20.390819000
6	17.209374000	1.311594000	16.682755000
6	18.519453000	0.136385000	14.975750000
6	18.069634000	-1.088793000	15.528446000
6	16.722457000	0.090384000	17.210889000
6	17.191020000	-1.108171000	16.618739000
5	23.205420000	0.011247000	5.884649000
8	23.412664000	1.197858000	5.189821000
8	23.362746000	-1.195116000	5.211004000
5	15.628789000	0.073865000	18.322972000
8	15.104044000	1.265385000	18.811186000
5	14.015675000	1.271407000	19.675715000
8	13.527224000	0.056207000	20.141843000
5	14.020112000	-1.150083000	19.657103000
8	15.102086000	-1.127419000	18.784246000
6	8.387268000	-9.637254000	20.423160000
6	8.761713000	-10.762689000	19.647326000
6	7.112496000	-9.657929000	21.042855000
6	6.233312000	-10.729381000	20.847429000
6	7.878829000	-11.832829000	19.456977000
6	6.584137000	-11.833587000	20.031997000
6	21.779767000	-9.689168000	-1.562539000
6	21.783132000	-9.697308000	-2.980063000
6	21.243979000	-10.820372000	-0.897662000
6	20.689902000	-11.885059000	-1.619344000
6	20.645260000	-11.873486000	-3.035181000
6	21.225333000	-10.762605000	-3.696011000
5	19.879757000	-12.972928000	-3.834172000
8	19.258054000	-14.028609000	-3.177312000
8	19.731372000	-12.863581000	-5.212156000
5	18.882205000	-13.711893000	-5.913371000

5	18.428958000	-14.912149000	-3.859443000
8	18.284295000	-14.767540000	-5.234862000
6	17.604126000	-15.986318000	-3.085760000
6	16.701035000	-16.843959000	-3.761111000
6	17.650696000	-16.072076000	-1.672524000
6	16.808624000	-16.939236000	-0.965676000
6	15.874687000	-17.762375000	-1.643170000
6	15.857997000	-17.713830000	-3.059781000
5	8.455680000	-22.157602000	3.827087000
8	8.374142000	-21.896608000	5.189821000
8	7.351949000	-22.708423000	3.186582000
5	6.141840000	-22.871991000	3.851200000
8	6.082863000	-22.588603000	5.211004000
5	7.181565000	-22.066191000	5.884649000
6	6.584690000	-19.789381000	11.297500000
6	7.272620000	-18.586335000	11.587891000
6	5.689165000	-20.278518000	12.279564000
6	5.470365000	-19.583970000	13.476079000
6	6.135663000	-18.364579000	13.751908000
6	7.053981000	-17.892557000	12.782912000
5	4.899811000	-14.841036000	18.322972000
8	5.870859000	-13.973774000	18.811186000
5	5.540262000	-12.936813000	19.675715000
8	4.233598000	-12.847786000	20.141843000
5	3.238659000	-13.689314000	19.657103000
8	3.594562000	-14.711329000	18.784246000
5	23.686081000	1.194754000	3.827087000
8	23.868871000	-0.025170000	3.186582000
5	23.650489000	-1.226597000	3.851200000
6	17.085586000	-12.230087000	-11.333197000
6	16.632727000	-13.144801000	-12.314990000
6	16.034154000	-12.702305000	-13.501606000
6	15.857437000	-11.322465000	-13.767341000
6	16.341143000	-10.408311000	-12.800179000
6	16.939121000	-10.850215000	-11.615177000
1	16.876543000	2.262107000	17.131874000
1	18.372957000	-2.039273000	15.059628000
1	16.829996000	-2.075848000	17.005107000
1	9.739891000	-10.770241000	19.139161000
1	6.809148000	-8.817817000	21.688862000
1	5.239023000	-10.707135000	21.323770000
1	8.183054000	-12.677073000	18.816069000
1	22.233242000	-8.852546000	-3.526692000
1	21.211927000	-10.837563000	0.204089000
1	20.246591000	-12.734984000	-1.074203000
1	21.222176000	-10.731068000	-4.798248000
1	16.652356000	-16.813709000	-4.862335000
1	18.342139000	-15.417175000	-1.116627000
1	16.834347000	-16.941244000	0.136334000
1	15.173149000	-18.373356000	-3.617565000
1	7.952629000	-18.154127000	10.836193000
1	5.167437000	-21.234920000	12.111994000

1	4.781301000	-20.008915000	14.224103000
1	7.567147000	-16.930700000	12.943379000
1	16.769155000	-14.226793000	-12.154720000
1	15.712971000	-13.445725000	-14.249342000
1	16.193865000	-9.326974000	-12.952635000
1	17.247948000	-10.106242000	-10.863313000
6	18.088354000	1.336965000	15.593790000
1	18.454498000	2.304774000	15.213910000
6	-10.916020000	-21.220463000	-0.965676000
6	-9.763918000	-21.682562000	-3.085760000
6	-10.858653000	-21.088698000	-3.761111000
6	-11.987474000	-20.586601000	-1.643170000
6	-11.946463000	-20.555726000	-3.059781000
5	-8.487435000	-22.135089000	-3.859443000
8	-7.390935000	-22.650576000	-3.177312000
8	-8.394608000	-21.952819000	-5.234862000
5	-18.460183000	-14.888905000	3.827087000
8	-18.237160000	-14.730706000	5.189821000
5	-18.766969000	-13.648903000	5.884649000
8	-19.603330000	-12.765408000	5.211004000
5	-19.854623000	-12.909070000	3.851200000
8	-19.325116000	-14.009407000	3.186582000
6	-5.626239000	-17.750790000	-14.976363000
6	-4.349541000	-17.702850000	-15.590393000
6	-6.649014000	-16.931225000	-15.515984000
6	-6.390834000	-16.068499000	-16.588478000
6	-5.104090000	-15.983872000	-17.174899000
6	-4.096796000	-16.837590000	-16.661143000
5	-4.775604000	-14.916849000	-18.264341000
8	-5.752572000	-14.035531000	-18.714193000
8	-3.477418000	-14.777640000	-18.742958000
5	-3.130776000	-13.730220000	-19.588245000
5	-5.435178000	-12.985446000	-19.568354000
8	-4.133035000	-12.881805000	-20.044376000
5	-6.194805000	-22.915628000	-3.834172000
8	-6.136663000	-22.740715000	-5.212156000
5	-7.205863000	-22.195252000	-5.913371000
6	-1.636613000	-13.446105000	-19.935485000
6	-0.589594000	-14.216541000	-19.372386000
6	0.754144000	-13.867543000	-19.556200000
6	1.114024000	-12.724579000	-20.313092000
6	0.071337000	-11.980901000	-20.920731000
6	-1.270039000	-12.333190000	-20.731949000
6	-1.212464000	-23.795063000	-0.799872000
6	-1.136741000	-24.410664000	0.473298000
6	0.051538000	-24.406040000	1.215112000
6	1.225175000	-23.785891000	0.721589000
6	-0.029542000	-23.203835000	-1.305938000
6	1.157035000	-23.199487000	-0.565413000
6	-16.786038000	-12.377667000	11.297500000
6	-17.527968000	-11.677125000	12.279564000
6	-15.429292000	-12.660166000	11.587891000

6	-14.837033000	-12.237839000	12.782912000
6	-15.569729000	-11.510329000	13.751908000
6	-16.935026000	-11.254405000	13.476079000
1	-10.909981000	-21.245548000	0.136334000
1	-10.844926000	-21.033054000	-4.862335000
1	-12.785340000	-20.108201000	-3.617565000
1	-3.544830000	-18.359662000	-15.221537000
1	-7.648022000	-16.932782000	-15.049942000
1	-7.197047000	-15.416702000	-16.964237000
1	-3.088556000	-16.808070000	-17.106705000
1	-0.840578000	-15.089003000	-18.746380000
1	1.540413000	-14.457891000	-19.057820000
1	-2.060654000	-11.722369000	-21.198586000
1	-2.022852000	-24.921338000	0.884435000
1	0.074294000	-24.912995000	2.193658000
1	-0.053904000	-22.684602000	-2.277525000
1	2.037912000	-22.677126000	-0.972094000
1	-18.598784000	-11.476476000	12.111994000
1	-14.808104000	-13.173333000	10.836193000
1	-13.763676000	-12.428659000	12.943379000
1	-17.552106000	-10.730382000	14.224103000
6	-9.831087000	-21.753354000	-1.672524000
1	-8.994571000	-22.208580000	-1.116627000
5	-12.018511000	-7.310379000	19.657103000
8	-12.880525000	-7.964682000	18.784246000
8	-10.910716000	-7.996575000	20.141843000
5	-10.591605000	-9.266797000	19.675715000
5	-12.600539000	-9.246129000	18.322972000
8	-11.475654000	-9.901652000	18.811186000
6	-5.168883000	-11.428863000	20.520312000
6	-4.835779000	-12.805418000	20.509584000
6	-3.501600000	-13.232035000	20.504946000
6	-2.431823000	-12.304022000	20.511282000
6	-2.766087000	-10.928904000	20.559557000
6	-4.098559000	-10.502861000	20.563779000
1	-5.639330000	-13.559838000	20.522619000
1	-3.284514000	-14.312686000	20.514353000
1	-1.964293000	-10.173458000	20.533377000
1	-4.313224000	-9.422310000	20.540569000
6	10.899431000	6.207951000	20.520312000
6	9.489231000	6.087921000	20.563779000
6	11.439063000	7.517401000	20.509584000
6	10.610449000	8.646752000	20.504946000
6	9.199509000	8.524773000	20.511282000
6	8.661660000	7.215804000	20.559557000
5	5.426247000	12.978524000	19.657103000
8	5.739041000	14.014546000	18.784246000
5	4.759312000	14.886687000	18.322972000
8	3.463954000	14.755825000	18.811186000
5	3.121901000	13.722586000	19.675715000
8	4.126686000	12.882524000	20.141843000
1	9.027766000	5.087559000	20.540569000

1	12.532587000	7.655424000	20.522619000
1	11.070013000	9.648617000	20.514353000
1	7.568955000	7.075918000	20.533377000
6	-12.453295000	-1.489351000	20.511282000
6	-11.248773000	-0.746512000	20.559557000
6	-13.666467000	-0.758704000	20.504946000
6	-13.673014000	0.642008000	20.509584000
6	-12.466767000	1.384187000	20.520312000
6	-11.255339000	0.652399000	20.563779000
1	-10.282533000	-1.275618000	20.533377000
1	-14.627144000	-1.299104000	20.514353000
1	-14.638821000	1.173102000	20.522619000
1	-10.294009000	1.190466000	20.540569000
5	-12.687372000	9.126614000	18.322972000
8	-12.963203000	7.854216000	18.811186000
8	-11.555164000	9.788885000	18.784246000
5	-10.666507000	9.171252000	19.657103000
5	-12.086234000	7.209617000	19.675715000
8	-10.976792000	7.905630000	20.141843000
6	-1.120807000	12.726615000	20.423160000
6	-0.762225000	13.857204000	19.647326000
6	0.581056000	14.204019000	19.456977000
6	1.628929000	13.443631000	20.031997000
6	1.263717000	12.344100000	20.847429000
6	-0.077342000	11.994049000	21.042855000
1	-1.549148000	14.438272000	19.139161000
1	0.831166000	15.065846000	18.816069000
1	2.055038000	11.741674000	21.323770000
1	-0.325734000	11.136081000	21.688862000
5	12.711023000	-9.151430000	-18.264341000
8	11.570941000	-9.808239000	-18.714193000
5	10.670330000	-9.181885000	-19.568354000
8	12.979790000	-7.873763000	-18.742958000
8	10.974146000	-7.911446000	-20.044376000
5	12.090752000	-7.220416000	-19.588245000
6	-20.628487000	-11.811454000	3.057616000
6	-20.821268000	-11.926743000	1.658703000
6	-21.063626000	-10.614901000	3.678568000
6	-21.621389000	-9.567518000	2.934533000
6	-21.379764000	-10.884276000	0.910557000
6	-21.767282000	-9.668817000	1.528388000
6	-6.487211000	-11.885419000	-19.910268000
6	-7.784109000	-11.898470000	-19.340486000
6	-6.141096000	-10.772560000	-20.715978000
6	-7.027516000	-9.706730000	-20.908258000
6	-8.305828000	-9.701093000	-20.295813000
6	-8.674835000	-10.834152000	-19.528469000
5	-14.025678000	-1.265326000	-19.588245000
8	-15.128952000	-1.259321000	-18.742958000
8	-13.528502000	-0.049947000	-20.044376000
5	-14.029455000	1.156438000	-19.568354000
8	-15.126226000	1.133804000	-18.714193000

5	-15.662509000	-0.067690000	-18.264341000
6	-16.778816000	-0.085010000	-17.174899000
6	-17.256927000	1.112605000	-16.588478000
6	-17.279479000	-1.306817000	-16.661143000
6	-18.180493000	-1.333822000	-15.590393000
6	-18.620608000	-0.134424000	-14.976363000
6	-18.157210000	1.091552000	-15.515984000
5	-23.335673000	-0.005527000	-5.913371000
8	-23.524038000	-1.190954000	-5.212156000
5	-23.708357000	-1.189709000	-3.834172000
8	-23.825903000	0.029783000	-3.177312000
5	-23.674482000	1.231912000	-3.859443000
8	-23.472448000	1.199952000	-5.234862000
1	-20.508916000	-12.852580000	1.147723000
1	-20.927493000	-10.492852000	4.766058000
1	-21.898806000	-8.629322000	3.442538000
1	-21.522001000	-11.010850000	-0.175169000
1	-8.084916000	-12.749671000	-18.707154000
1	-5.145034000	-10.739845000	-21.187896000
1	-6.728008000	-8.859912000	-21.547290000
1	-9.655891000	-10.852919000	-19.026106000
1	-16.886165000	2.080776000	-16.964237000
1	-16.939841000	-2.256588000	-17.106705000
1	-18.556489000	-2.302113000	-15.221537000
1	-18.467402000	2.041184000	-15.049942000
1	0.320670000	-11.112563000	-21.552272000
6	-13.581878000	9.756091000	17.210889000
6	-14.693611000	9.054314000	16.682755000
6	-13.256460000	11.001157000	16.618739000
6	-13.978665000	11.501916000	15.528446000
6	-15.419634000	9.550441000	15.593790000
6	-15.062717000	10.775124000	14.975750000
6	-22.984734000	6.283898000	-0.888808000
6	-23.590913000	6.571049000	0.358530000
6	-22.025512000	7.206619000	-1.371902000
6	-21.664568000	8.336861000	-0.630739000
6	-22.243112000	8.605037000	0.633464000
6	-23.229202000	7.702368000	1.100846000
5	-19.864701000	12.955753000	3.827087000
8	-19.645326000	12.792531000	5.189821000
5	-18.780190000	13.630705000	5.884649000
8	-18.198387000	14.699147000	5.211004000
5	-18.412671000	14.893747000	3.851200000
8	-19.295528000	14.050133000	3.186582000
1	-14.983043000	8.089700000	17.131874000
1	-12.395600000	11.571819000	17.005107000
1	-13.665380000	12.449160000	15.059628000
1	-16.284715000	8.982680000	15.213910000
1	-24.376263000	5.903063000	0.748355000
1	-21.506813000	7.003999000	-2.322775000
1	-20.870506000	8.996641000	-1.015983000
1	-23.737355000	7.899798000	2.058893000

6	8.303572000	9.705507000	20.406825000
6	7.030434000	9.718602000	21.029863000
6	8.665934000	10.829023000	19.622478000
6	7.773292000	11.890475000	19.428260000
6	6.480935000	11.884062000	20.008380000
6	6.141935000	10.781631000	20.831248000
6	5.830573000	18.463709000	13.751908000
6	7.085550000	19.059164000	13.476079000
6	7.316783000	19.749673000	12.279564000
6	6.304780000	19.880329000	11.297500000
6	5.041101000	19.311399000	11.587891000
6	4.810191000	18.621609000	12.782912000
6	15.945264000	17.719672000	-1.562539000
6	15.954046000	17.720357000	-2.980063000
6	16.794834000	16.860664000	-3.696011000
6	17.672093000	15.965700000	-3.035181000
6	17.696894000	16.004581000	-1.619344000
6	16.855536000	16.860546000	-0.897662000
6	23.052970000	6.112552000	0.633464000
6	23.320157000	7.422436000	1.100846000
6	22.427288000	5.989452000	-0.630739000
6	19.361777000	0.160395000	13.751908000
6	19.196633000	1.179631000	12.782912000
6	20.315898000	-0.849153000	13.476079000
6	21.044066000	-0.855689000	12.279564000
6	20.855601000	0.147158000	11.297500000
6	19.924018000	1.173179000	11.587891000
6	22.255778000	-8.506201000	-0.799872000
6	22.059029000	-7.198476000	-1.305938000
6	22.421567000	-6.068630000	-0.565413000
6	23.000326000	-6.185034000	0.721589000
6	23.227449000	-7.492866000	1.215112000
6	22.864649000	-8.624415000	0.473298000
6	14.901458000	-18.593868000	-0.888808000
6	13.583069000	-18.776548000	-1.371902000
6	12.626720000	-19.478776000	-0.630739000
6	12.937142000	-20.035795000	0.633464000
6	14.265481000	-19.885129000	1.100846000
6	15.223084000	-19.182481000	0.358530000
6	6.765603000	-20.466848000	9.987507000
6	5.680186000	-21.110362000	9.342135000
6	8.012085000	-20.439181000	9.313157000
6	8.151176000	-20.987428000	8.032857000
6	7.057669000	-21.592848000	7.365760000
6	5.825208000	-21.657544000	8.061223000
6	9.272222000	-8.447613000	20.520312000
6	8.722290000	-7.143524000	20.563779000
6	9.539237000	-6.007922000	20.559557000
6	10.684338000	-8.556191000	20.509584000
6	-17.374443000	-12.759074000	9.987507000
6	-16.962945000	-13.936000000	9.313157000
6	-17.441378000	-14.237701000	8.032857000

6	-18.355079000	-13.384799000	7.365760000
6	-18.797460000	-12.232651000	8.061223000
6	-18.321873000	-11.925638000	9.342135000
6	-23.005122000	-6.199957000	-0.799872000
6	-22.077287000	-7.142283000	-1.305938000
6	-21.706480000	-8.269442000	-0.565413000
6	-22.243127000	-8.515455000	0.721589000
6	-23.195597000	-7.590896000	1.215112000
6	-23.567194000	-6.462206000	0.473298000
6	-23.283354000	5.039156000	-1.643170000
6	-23.241317000	5.009692000	-3.059781000
6	-23.412052000	3.810427000	-3.761111000
6	-23.638559000	2.585757000	-3.085760000
6	-23.726642000	2.627763000	-1.672524000
6	-23.555095000	3.824269000	-0.965676000
6	-13.005483000	19.963279000	-0.799872000
6	-13.428586000	20.416802000	0.473298000
6	-14.387205000	19.714608000	1.215112000
6	-14.972183000	18.523050000	0.721589000
6	-14.572378000	18.088691000	-0.565413000
6	-13.614972000	18.789661000	-1.305938000
6	1.310377000	23.813560000	0.633464000
6	1.234106000	23.180460000	-0.630739000
6	0.047645000	23.174474000	-1.371902000
6	-1.126331000	23.801613000	-0.888808000
6	-1.040554000	24.466857000	0.358530000
6	0.147169000	24.472447000	1.100846000
6	-15.758285000	11.250805000	13.751908000
6	-16.223772000	10.329157000	12.782912000
6	-16.808446000	10.761922000	11.587891000
6	-16.959032000	12.139562000	11.297500000
6	-16.522045000	13.061659000	12.279564000
6	-15.936786000	12.628364000	13.476079000
6	15.143401000	-10.836168000	-14.976363000
6	15.492329000	-9.607141000	-15.590393000
6	14.747520000	-9.099386000	-16.661143000
6	13.624315000	-9.793566000	-17.174899000
6	13.307175000	-11.043483000	-16.588478000
6	14.047894000	-11.555625000	-15.515984000
6	-12.450079000	2.866790000	20.423160000
6	-11.430919000	3.632809000	21.042855000
6	-13.414525000	3.557192000	19.647326000
6	-13.329269000	4.941901000	19.456977000
6	-12.282286000	5.703514000	20.031997000
6	-11.349427000	5.016403000	20.847429000
6	12.421715000	2.966306000	-20.295813000
6	13.386243000	3.666073000	-19.528469000
6	13.291222000	5.050680000	-19.340486000
6	12.234337000	5.802419000	-19.910268000
6	11.300203000	5.105538000	-20.715978000
6	11.390853000	3.722239000	-20.908258000
6	-6.573765000	-10.954842000	20.423160000

6	-6.987354000	-9.748850000	21.042855000
6	-8.278048000	-9.243793000	20.847429000
6	-9.219800000	-9.918665000	20.031997000
6	-8.818997000	-11.149756000	19.456977000
6	-7.528407000	-11.658738000	19.647326000
6	2.469131000	-23.689744000	1.528388000
6	3.744851000	-23.696790000	0.910557000
6	2.417874000	-23.519689000	2.934533000
6	3.586352000	-23.312884000	3.678568000
6	4.858807000	-23.268796000	3.057616000
6	4.908881000	-23.487769000	1.658703000
6	-5.868086000	-18.580153000	-13.767341000
6	-4.849201000	-18.757696000	-12.800179000
6	-5.084692000	-19.462962000	-11.615177000
6	-6.351767000	-20.028663000	-11.333197000
6	-7.361654000	-19.880630000	-12.314990000
6	-7.125784000	-19.174614000	-13.501606000
6	-13.079016000	-19.917950000	-0.888808000
6	-13.660160000	-18.720539000	-1.371902000
6	-14.623545000	-18.027997000	-0.630739000
6	-15.057377000	-18.495354000	0.633464000
6	-14.503605000	-19.712121000	1.100846000
6	-13.539432000	-20.405726000	0.358530000
6	-19.484114000	-0.160701000	-13.767341000
6	-20.438131000	0.851742000	-13.501606000
6	-21.182479000	0.857896000	-12.314990000
6	-21.011194000	-0.148308000	-11.333197000
6	-20.081633000	-1.178557000	-11.615177000
6	-19.338114000	-1.184583000	-12.800179000
6	-11.792929000	4.901509000	-20.295813000
6	-11.403271000	3.684021000	-20.908258000
6	-12.143016000	2.511626000	-20.715978000
6	-13.308363000	2.496908000	-19.910268000
6	-13.721540000	3.726298000	-19.340486000
6	-12.984562000	4.902321000	-19.528469000
6	1.017397000	12.730392000	-20.295813000
6	-0.020093000	11.983580000	-20.908258000
6	-1.363701000	12.324830000	-20.715978000
6	-1.737810000	13.428592000	-19.910268000
6	-0.696269000	14.201449000	-19.340486000
6	0.649934000	13.863953000	-19.528469000
6	14.985376000	11.053670000	-14.976363000
6	15.331090000	9.789456000	-15.515984000
6	14.615120000	9.243251000	-16.588478000
6	13.524380000	9.931115000	-17.174899000
6	13.211264000	11.213860000	-16.661143000
6	13.924327000	11.765310000	-15.590393000
6	-2.536003000	12.284337000	20.520312000
6	-3.614608000	13.202200000	20.509584000
6	-4.944741000	12.763130000	20.504946000
6	-5.264737000	11.383553000	20.511282000
6	-4.186037000	10.467534000	20.559557000

6	-2.857623000	10.906066000	20.563779000
6	2.529573000	-12.282036000	-20.404878000
6	2.851858000	-10.903398000	-20.428577000
6	4.180460000	-10.465633000	-20.424212000
6	5.258908000	-11.382764000	-20.395395000
6	4.938176000	-12.762121000	-20.406187000
6	3.607723000	-13.200468000	-20.411116000
1	6.736439000	8.879729000	21.681812000
1	9.642681000	10.841646000	19.111637000
1	8.068399000	12.733539000	18.781524000
1	5.149663000	10.754442000	21.311462000
1	7.892791000	18.997931000	14.224103000
1	8.301028000	20.216755000	12.111994000
1	4.236916000	19.361435000	10.836193000
1	3.829665000	18.145082000	12.943379000
1	15.289721000	18.409483000	-3.526692000
1	16.763866000	16.867406000	-4.798248000
1	18.368231000	15.320326000	-1.074203000
1	16.861981000	16.824750000	0.204089000
1	23.847308000	7.561396000	2.058893000
1	22.172687000	4.988940000	-1.015983000
1	18.440430000	1.964910000	12.943379000
1	20.507112000	-1.635807000	14.224103000
1	21.792435000	-1.647426000	12.111994000
1	19.723099000	1.953466000	10.836193000
1	21.557682000	-7.061193000	-2.277525000
1	22.196978000	-5.069447000	-0.972094000
1	23.716625000	-7.627881000	2.193658000
1	23.076505000	-9.624963000	0.884435000
1	13.282530000	-18.307742000	-2.322775000
1	11.596501000	-19.545811000	-1.015983000
1	14.560538000	-20.343538000	2.058893000
1	16.251078000	-19.103686000	0.748355000
1	4.693453000	-21.133109000	9.833040000
1	8.886066000	-19.983373000	9.806617000
1	9.128643000	-20.935685000	7.525165000
1	4.953843000	-22.120843000	7.569203000
1	7.628290000	-7.013773000	20.540569000
1	11.153523000	-9.553542000	20.522619000
1	-16.259371000	-14.626352000	9.806617000
1	-17.090114000	-15.151338000	7.525165000
1	-19.507350000	-11.547101000	7.569203000
1	-18.648425000	-10.994229000	9.833040000
1	-21.590996000	-6.958662000	-2.277525000
1	-20.937479000	-8.945787000	-0.972094000
1	-23.670708000	-7.769197000	2.193658000
1	-24.326697000	-5.777271000	0.884435000
1	-23.074923000	5.945805000	-3.617565000
1	-23.354889000	3.814567000	-4.862335000
1	-23.901090000	1.691517000	-1.116627000
1	-23.577087000	3.810774000	0.136334000
1	-13.011873000	21.350789000	0.884435000

1	-14.703597000	20.111367000	2.193658000
1	-14.977986000	17.148326000	-0.972094000
1	-13.290066000	18.383912000	-2.277525000
1	2.106973000	22.629146000	-1.015983000
1	0.015229000	22.618549000	-2.322775000
1	-1.918533000	25.007351000	0.748355000
1	0.177909000	25.016738000	2.058893000
1	-16.073566000	9.249367000	12.943379000
1	-17.104540000	10.012560000	10.836193000
1	-16.662118000	14.142068000	12.111994000
1	-15.629099000	13.377174000	14.224103000
1	16.365663000	-9.044782000	-15.221537000
1	15.031008000	-8.131371000	-17.106705000
1	12.438145000	-11.608822000	-16.964237000
1	13.740664000	-12.506219000	-15.049942000
1	-10.691700000	3.131447000	21.688862000
1	-14.210326000	2.988344000	19.139161000
1	-14.071626000	5.446089000	18.816069000
1	-10.531954000	5.582834000	21.323770000
1	14.190965000	3.104605000	-19.026106000
1	14.034903000	5.562506000	-18.707154000
1	10.475143000	5.664542000	-21.187896000
1	10.650799000	3.213195000	-21.547290000
1	-6.282100000	-9.200740000	21.688862000
1	-8.564143000	-8.291293000	21.323770000
1	-9.527910000	-11.699978000	18.816069000
1	-7.233316000	-12.591374000	19.139161000
1	3.821277000	-23.871180000	-0.175169000
1	1.439870000	-23.493610000	3.442538000
1	3.512344000	-23.145698000	4.766058000
1	5.885927000	-23.476803000	1.147723000
1	-3.866300000	-18.283474000	-12.952635000
1	-4.281698000	-19.526774000	-10.863313000
1	-8.348530000	-20.344735000	-12.154720000
1	-7.932070000	-19.098881000	-14.249342000
1	-13.307170000	-18.289840000	-2.322775000
1	-15.005655000	-17.068916000	-1.015983000
1	-14.848401000	-20.134394000	2.058893000
1	-13.146826000	-21.359057000	0.748355000
1	-20.615259000	1.641968000	-14.249342000
1	-21.928831000	1.653055000	-12.154720000
1	-19.894183000	-1.961968000	-10.863313000
1	-18.583370000	-1.972834000	-12.952635000
1	-10.505346000	3.660853000	-21.547290000
1	-11.804103000	1.574423000	-21.187896000
1	-14.624034000	3.749347000	-18.707154000
1	-13.305573000	5.829561000	-19.026106000
1	0.235347000	11.122444000	-21.547290000
1	-2.150303000	11.712892000	-21.187896000
1	-0.953234000	15.066895000	-18.707154000
1	1.432594000	14.455786000	-19.026106000
1	16.140219000	9.203514000	-15.049942000

1	14.884244000	8.242056000	-16.964237000
1	12.378230000	11.782606000	-17.106705000
1	13.659367000	12.769679000	-15.221537000
1	-3.407959000	14.284855000	20.522619000
1	-5.755558000	13.509795000	20.514353000
1	-4.390662000	9.385083000	20.533377000
1	-2.048823000	10.158058000	20.540569000
1	2.043616000	-10.155396000	-20.390819000
1	4.385827000	-9.383786000	-20.383464000
1	5.748544000	-13.508965000	-20.430115000
1	3.400438000	-14.282724000	-20.438928000
6	-9.200560000	-8.518986000	-20.395395000
6	-10.611518000	-8.640197000	-20.406187000
6	-8.661576000	-7.209912000	-20.424212000
6	-9.488475000	-6.081613000	-20.428577000
6	-10.899229000	-6.201125000	-20.404878000
6	-11.439544000	-7.510318000	-20.411116000
1	-11.071392000	-9.641690000	-20.430115000
1	-7.569216000	-7.070919000	-20.383464000
1	-9.026843000	-5.081784000	-20.390819000
1	-12.532884000	-7.647613000	-20.438928000
6	-5.881929000	17.667712000	-14.976363000
6	-4.572759000	17.605842000	-15.515984000
6	-4.274534000	16.756127000	-16.588478000
6	-5.265789000	15.931333000	-17.174899000
6	-6.582510000	16.029933000	-16.661143000
6	-6.886622000	16.878503000	-15.590393000
1	-3.765460000	18.194303000	-15.049942000
1	-3.239177000	16.702693000	-16.964237000
1	-7.380841000	15.413422000	-17.106705000
1	-7.923710000	16.936877000	-15.221537000
6	22.947815000	8.550300000	0.358530000
6	22.288623000	8.426307000	-0.888808000
1	23.190544000	9.552330000	0.748355000
6	22.054958000	7.115994000	-1.371902000
1	21.516225000	6.975033000	-2.322775000
6	11.502359000	-7.419143000	20.504946000
6	10.950346000	-6.114953000	20.511282000
1	9.068533000	-5.011925000	20.533377000
1	12.597202000	-7.546622000	20.514353000
6	6.578055000	10.949208000	-20.313092000
6	7.541022000	11.662353000	-19.556200000
6	8.833265000	11.154868000	-19.372386000
6	9.227470000	9.916151000	-19.935485000
6	8.276750000	9.231250000	-20.731949000
6	6.984484000	9.734683000	-20.920731000
6	-1.012977000	-12.732637000	20.406825000
6	-0.645748000	-13.854572000	19.622478000
6	0.700321000	-14.188623000	19.428260000
6	1.742090000	-13.423806000	20.008380000
6	1.368354000	-12.332661000	20.831248000
6	0.024710000	-11.994900000	21.029863000

6	-2.484628000	-23.707907000	-1.562539000
6	-3.726034000	-23.547903000	-0.897662000
6	-2.491330000	-23.713622000	-2.980063000
6	-3.676857000	-23.512319000	-3.696011000
6	-4.912621000	-23.303918000	-3.035181000
6	-4.909831000	-23.349951000	-1.619344000
6	2.548638000	23.722639000	1.449479000
6	3.828309000	23.684643000	0.841040000
6	2.484567000	23.599814000	2.860004000
6	3.643303000	23.392773000	3.618664000
6	4.918783000	23.302826000	3.008931000
6	4.983012000	23.476908000	1.604291000
6	-6.664541000	10.896333000	20.406825000
6	-7.621092000	11.588145000	19.622478000
6	-7.070416000	9.689554000	21.029863000
6	-8.355978000	9.173034000	20.831248000
6	-9.299696000	9.836112000	20.008380000
6	-8.906435000	11.067199000	19.428260000
6	6.659646000	-10.897114000	-20.295813000
6	7.060027000	-9.683110000	-20.908258000
6	7.623219000	-11.598195000	-19.528469000
6	8.910696000	-11.079957000	-19.340486000
6	9.299047000	-9.842500000	-19.910268000
6	8.347610000	-9.169433000	-20.715978000
6	-14.902387000	-10.995799000	14.975750000
6	-13.847938000	-11.713695000	15.593790000
6	-13.151740000	-11.176518000	16.682755000
6	-13.475625000	-9.902336000	17.210889000
6	-14.559194000	-9.208098000	16.618739000
6	-15.258617000	-9.740213000	15.528446000
6	-16.005709000	-17.693966000	1.449479000
6	-15.881679000	-17.632259000	2.860004000
6	-17.018650000	-16.911055000	0.841040000
6	-17.830722000	-16.064277000	1.604291000
6	-17.676436000	-15.961194000	3.008931000
6	-16.697421000	-16.783671000	3.618664000
6	-6.608841000	-20.691517000	-10.028407000
6	-7.864468000	-20.569038000	-9.382462000
6	-8.069493000	-21.093517000	-8.100232000
6	-7.035946000	-21.767012000	-7.403849000
6	-5.798266000	-21.930143000	-8.073904000
6	-5.588007000	-21.408949000	-9.355770000
6	5.852535000	-17.570901000	14.975750000
6	6.861138000	-16.789902000	15.593790000
6	6.565389000	-15.961782000	16.682755000
6	5.253484000	-15.876071000	17.210889000
6	4.258384000	-16.692075000	16.618739000
6	4.548321000	-17.521699000	15.528446000
6	11.881926000	-20.690070000	1.449479000
6	10.824317000	-21.411502000	0.841040000
6	11.861566000	-20.553042000	2.860004000
6	10.802433000	-21.066631000	3.618664000

6	9.717678000	-21.743570000	3.008931000
6	9.768039000	-21.922159000	1.604291000
6	17.636558000	-12.679411000	-10.028407000
6	18.634331000	-11.930239000	-9.355770000
6	19.065042000	-12.291265000	-8.073904000
6	18.527432000	-13.417959000	-7.403849000
6	17.567517000	-14.192800000	-8.100232000
6	17.132064000	-13.835736000	-9.382462000
6	12.446047000	-2.872611000	-20.313092000
6	13.421860000	-3.568073000	-19.556200000
6	11.416558000	-3.634457000	-20.920731000
6	13.338539000	-4.953890000	-19.372386000
6	12.282265000	-5.711587000	-19.935485000
6	11.337097000	-5.019044000	-20.731949000
6	17.508833000	12.855210000	-10.028407000
6	18.452665000	12.018083000	-9.382462000
6	18.926816000	12.321885000	-8.100232000
6	18.486529000	13.474257000	-7.403849000
6	17.581110000	14.333723000	-8.073904000
6	17.104657000	14.035655000	-9.355770000
6	11.926913000	20.616724000	1.528388000
6	10.898975000	21.372274000	0.910557000
6	9.834396000	21.887372000	1.658703000
6	9.746198000	21.680787000	3.057616000
1	10.763154000	20.789767000	4.766058000
1	12.644318000	19.853064000	3.442538000
1	1.967442000	10.169567000	-20.383464000
1	7.251915000	12.602111000	-19.057820000
1	9.549136000	11.713180000	-18.746380000
1	8.557339000	8.272374000	-21.198586000
1	6.272373000	9.178738000	-21.552272000
1	-1.428534000	-14.438902000	19.111637000
1	0.957115000	-15.044135000	18.781524000
1	2.155138000	-11.727423000	21.311462000
1	-0.230520000	-11.143431000	21.681812000
1	-3.752289000	-23.522733000	0.204089000
1	-1.548821000	-23.880657000	-3.526692000
1	-3.647839000	-23.499571000	-4.798248000
1	-5.855149000	-23.190979000	-1.074203000
1	3.915208000	23.821593000	-0.249279000
1	1.502822000	23.609478000	3.361283000
1	3.558397000	23.260674000	4.710164000
1	5.963043000	23.429581000	1.101021000
1	-7.331265000	12.520988000	19.111637000
1	-6.363450000	9.150721000	21.681812000
1	-8.636749000	8.220926000	21.311462000
1	-9.617043000	11.608383000	18.781524000
1	6.347208000	-9.136580000	-21.547290000
1	7.337905000	-12.537034000	-19.026106000
1	9.627281000	-11.629077000	-18.707154000
1	8.624297000	-8.212013000	-21.187896000
1	-13.575290000	-12.711883000	15.213910000

1	-12.323777000	-11.749866000	17.131874000
1	-14.835905000	-8.213027000	17.005107000
1	-16.062689000	-9.149547000	15.059628000
1	-15.093111000	-18.217132000	3.361283000
1	-17.169451000	-16.970772000	-0.249279000
1	-18.595765000	-15.449941000	1.101021000
1	-16.551085000	-16.726707000	4.710164000
1	-8.673807000	-20.003259000	-9.872494000
1	-9.044828000	-20.948570000	-7.606540000
1	-4.978283000	-22.464537000	-7.565914000
1	-4.614443000	-21.559733000	-9.850283000
1	7.894724000	-16.839056000	15.213910000
1	7.366530000	-15.351517000	17.131874000
1	3.226506000	-16.647749000	17.005107000
1	3.738092000	-18.103891000	15.059628000
1	10.834511000	-21.573375000	-0.249279000
1	12.661494000	-19.983805000	3.361283000
1	10.793477000	-20.909854000	4.710164000
1	8.947359000	-22.459918000	1.101021000
1	19.078583000	-11.050920000	-9.850283000
1	19.826670000	-11.676552000	-7.565914000
1	17.128268000	-15.075606000	-7.606540000
1	16.343876000	-14.430628000	-9.872494000
1	14.226285000	-3.002714000	-19.057820000
1	10.667768000	-3.128996000	-21.552272000
1	14.090742000	-5.462196000	-18.746380000
1	10.511858000	-5.582209000	-21.198586000
1	18.774878000	11.084641000	-9.872494000
1	19.630679000	11.631333000	-7.606540000
1	17.231839000	15.248031000	-7.565914000
1	16.405656000	14.729889000	-9.850283000
1	10.939649000	21.558280000	-0.175169000
1	9.037504000	22.452794000	1.147723000
6	-23.315352000	-4.963124000	-1.562539000
6	-23.546795000	-3.733033000	-0.897662000
6	-23.724345000	-2.546005000	-1.619344000
6	-23.681426000	-2.529127000	-3.035181000
6	-23.497756000	-3.768807000	-3.696011000
6	-23.322859000	-4.958517000	-2.980063000
6	21.798571000	9.608849000	-1.643170000
6	21.747244000	9.607977000	-3.059781000
6	21.180461000	10.678559000	-3.761111000
6	20.643866000	11.802475000	-3.085760000
6	20.739817000	11.820265000	-1.672524000
6	21.304321000	10.751439000	-0.965676000
6	-11.757542000	-4.991611000	-20.313092000
6	-12.955774000	-5.002540000	-19.556200000
6	-13.702928000	-3.832416000	-19.372386000
6	-13.293747000	-2.598564000	-19.935485000
6	-12.122025000	-2.603287000	-20.731949000
6	-11.372470000	-3.770147000	-20.920731000
6	23.293289000	-4.972250000	1.528388000

6	23.115718000	-4.968449000	2.934533000
6	23.280114000	-3.793254000	3.678568000
6	23.631394000	-2.569453000	3.057616000
6	23.855123000	-2.589496000	1.658703000
6	23.694209000	-3.761145000	0.910557000
6	21.555815000	0.109867000	9.987507000
6	21.832421000	-1.121283000	9.342135000
6	22.397637000	-1.152447000	8.061223000
6	22.716958000	0.039685000	7.365760000
6	22.479082000	1.266757000	8.032857000
6	21.914686000	1.303892000	9.313157000
6	11.757382000	4.998691000	20.423160000
6	12.943444000	5.007031000	19.647326000
6	13.688381000	3.836666000	19.456977000
6	13.289020000	2.605107000	20.031997000
6	12.130447000	2.612671000	20.847429000
6	11.383119000	3.779921000	21.042855000
6	6.982184000	21.617375000	7.365760000
6	5.741660000	21.770327000	8.032857000
6	8.017293000	20.945292000	8.061223000
6	5.531936000	21.245030000	9.313157000
6	6.556624000	20.534749000	9.987507000
6	7.812993000	20.417371000	9.342135000
6	-11.925052000	20.640527000	-1.562539000
6	-11.922989000	20.649091000	-2.980063000
6	-10.845555000	21.183068000	-3.696011000
6	-9.723306000	21.740831000	-3.035181000
6	-9.752620000	21.776434000	-1.619344000
6	-10.826685000	21.240762000	-0.897662000
6	-21.773998000	9.754597000	1.449479000
6	-21.342421000	10.959895000	0.841040000
6	-20.788031000	11.993890000	1.604291000
6	-20.642317000	11.879010000	3.008931000
6	-21.122007000	10.693752000	3.618664000
6	-21.676983000	9.655707000	2.860004000
6	-21.721046000	-0.108649000	-10.028407000
6	-21.992572000	1.123371000	-9.382462000
6	-22.087909000	-1.301219000	-9.355770000
6	-22.554738000	1.156289000	-8.100232000
6	-22.875886000	-0.034794000	-7.403849000
6	-22.648568000	-1.262308000	-8.073904000
6	-6.815504000	20.624368000	-10.028407000
6	-8.063072000	20.604751000	-9.355770000
6	-8.199319000	21.149993000	-8.073904000
6	-7.102129000	21.745508000	-7.403849000
6	-5.870101000	21.808143000	-8.100232000
6	-5.727689000	21.263320000	-9.382462000
6	-12.422485000	-2.971203000	20.406825000
6	-11.400192000	-3.730129000	21.029863000
6	-11.306213000	-5.112384000	20.831248000
6	-12.228463000	-5.805011000	20.008380000
6	-13.277772000	-5.050570000	19.428260000

6	-13.376028000	-3.667156000	19.622478000
1	-23.530969000	-3.700285000	0.204089000
1	-23.865272000	-1.597829000	-1.074203000
1	-23.476665000	-3.792466000	-4.798248000
1	-23.190467000	-5.906512000	-3.526692000
1	22.162861000	8.752842000	-3.617565000
1	21.136649000	10.641610000	-4.862335000
1	20.330637000	12.680242000	-1.116627000
1	21.314180000	10.775283000	0.136334000
1	-13.274257000	-5.932754000	-19.057820000
1	-14.610248000	-3.863321000	-18.746380000
1	-11.785412000	-1.662613000	-21.198586000
1	-10.469583000	-3.738946000	-21.552272000
1	22.788695000	-5.890527000	3.442538000
1	23.098241000	-3.811976000	4.766058000
1	24.146618000	-1.656882000	1.147723000
1	23.883680000	-3.742350000	-0.175169000
1	21.549138000	-2.066751000	9.833040000
1	22.568993000	-2.124331000	7.569203000
1	22.731926000	2.212372000	7.525165000
1	21.751262000	2.275949000	9.806617000
1	13.252900000	5.934999000	19.139161000
1	14.585315000	3.865116000	18.816069000
1	11.802038000	1.673920000	21.323770000
1	10.490385000	3.751029000	21.688862000
1	4.920460000	22.303007000	7.525165000
1	8.994562000	20.807934000	7.569203000
1	4.556954000	21.389986000	9.806617000
1	8.624647000	19.855787000	9.833040000
1	-12.783675000	20.230232000	-3.526692000
1	-10.861538000	21.155699000	-4.798248000
1	-8.894400000	22.203466000	-1.074203000
1	-10.790650000	21.235831000	0.204089000
1	-21.445815000	11.084861000	-0.249279000
1	-20.440174000	12.911330000	1.101021000
1	-21.022610000	10.572181000	4.710164000
1	-21.989550000	8.724999000	3.361283000
1	-21.704584000	2.067934000	-9.872494000
1	-21.930465000	-2.273728000	-9.850283000
1	-22.718279000	2.128678000	-7.606540000
1	-22.903418000	-2.207295000	-7.565914000
1	-8.939330000	20.154492000	-9.850283000
1	-9.176808000	21.100353000	-7.565914000
1	-4.995841000	22.264165000	-7.606540000
1	-4.740363000	21.281313000	-9.872494000
1	-10.669267000	-3.224272000	21.681812000
1	-10.487468000	-5.673631000	21.311462000
1	-14.012058000	-5.559164000	18.781524000
1	-14.173653000	-3.103250000	19.111637000
6	-18.401732000	13.320587000	7.365760000
6	-17.442677000	14.097350000	8.061223000
6	-17.003726000	13.739912000	9.342135000

6	-17.503599000	12.581306000	9.987507000
6	-18.495762000	11.826259000	9.313157000
6	-18.930540000	12.188045000	8.032857000
6	11.796430000	-4.898000000	20.406825000
6	12.976934000	-4.895441000	19.622478000
6	13.710593000	-3.718481000	19.428260000
6	13.305134000	-2.491358000	20.008380000
6	12.151903000	-2.509620000	20.831248000
6	11.415464000	-3.683127000	21.029863000
6	-6.579375000	11.840105000	-19.935485000
6	-7.879281000	11.847978000	-19.372386000
6	-8.761253000	10.775803000	-19.556200000
6	-8.380585000	9.639594000	-20.313092000
6	-7.099910000	9.650822000	-20.920731000
6	-6.221784000	10.724271000	-20.731949000
1	-17.010049000	14.984342000	7.569203000
1	-16.218813000	14.338302000	9.833040000
1	-18.934910000	10.943790000	9.806617000
1	-19.690914000	11.571644000	7.525165000
1	13.290770000	-5.820482000	19.111637000
1	14.603588000	-3.738623000	18.781524000
1	11.819416000	-1.574315000	21.311462000
1	10.526798000	-3.662747000	21.681812000
1	-8.189051000	12.701339000	-18.746380000
1	-9.744355000	10.791247000	-19.057820000
1	-6.791228000	8.801768000	-21.552272000
1	-5.223132000	10.694817000	-21.198586000
